# Systematic Roadmap for Cancer Drug Screening Using Zebrafish Embryo Xenograft Cancer Models: Melanoma Cell Line as a Case Study

**DOI:** 10.3390/cancers13153705

**Published:** 2021-07-23

**Authors:** Patricia Letrado, Holly Mole, María Montoya, Irene Palacios, Jorge Barriuso, Adam Hurlstone, Roberto Díez-Martínez, Julen Oyarzabal

**Affiliations:** 1 Ikan Biotech SL, Centro Europeo de Empresas e Innovación de Navarra (CEIN), 31110 Noain, Spain; roberto.diez@ikanbiotech.com; 2 Center for Applied Medical Research (CIMA), University of Navarra, 31008 Pamplona, Spain; 3 Division of Cancer Sciences, School of Medical Sciences, The University of Manchester, Manchester M13 9PL, UK; holly.mole@manchester.ac.uk (H.M.); jorge.barriuso@manchester.ac.uk (J.B.); 4 Cellomics Unit, Spanish National Center for Cardiovascular Research (CNIC), 28029 Madrid, Spain; mmontoya68@gmail.com (M.M.); irene.palacios@cnic.es (I.P.); 5 The Christie NHS Foundation Trust, Manchester M20 4BX, UK; 6 Division of Infection, Immunology and Respiratory Medicine, School of Biological Science, The University of Manchester, Manchester M13 9PT, UK; adam.hurlstone@manchester.ac.uk

**Keywords:** zebrafish, roadmap, drug discovery, screening, xenograft, cancer

## Abstract

**Simple Summary:**

Currently, there is no consensus in the scientific literature regarding the zebrafish embryo xenotransplantation procedure for drug screening. Thus, this study sets systematic guidelines for maximizing the reproducibility of drug screening in zebrafish-embryo cancer xenograft models based on evaluating every step of the procedure in a real case scenario in which the chemical properties of the compounds are unknown or not optimal. It aims to be a stepping stone to bring the versatility of zebrafish embryos to drug screening for cancer. The present work helps our group to pursue the objective of establishing zebrafish embryos as a valuable alternative to mice models; and hopefully, will help other groups in this field to progress in the same direction.

**Abstract:**

Zebrafish embryo tumor transplant models are widely utilized in cancer research. Compared with traditional murine models, the small size and transparency of zebrafish embryos combined with large clutch sizes that increase statistical power and cheap husbandry make them a cost-effective and versatile tool for in vivo drug discovery. However, the lack of a comprehensive analysis of key factors impacting the successful use of these models impedes the establishment of basic guidelines for systematic screening campaigns. Thus, we explored the following crucial factors: (i) user-independent inclusion criteria, focusing on sample homogeneity; (ii) metric definition for data analysis; (iii) tumor engraftment criteria; (iv) image analysis versus quantification of human cancer cells using qPCR (RNA and gDNA); (v) tumor implantation sites; (vi) compound distribution (intratumoral administration versus alternative inoculation sites); and (vii) efficacy (intratumoral microinjection versus compound solution in media). Based on these analyses and corresponding assessments, we propose the first roadmap for systematic drug discovery screening in zebrafish xenograft cancer models using a melanoma cell line as a case study. This study aims to help the wider cancer research community to consider the adoption of this versatile model for cancer drug screening projects.

## 1. Introduction

Cancer research studies based on zebrafish models have recently flourished due to their versatility for studying cancer processes such as tumor growth and angiogenesis and to the probability of their becoming powerful tools for developing precision medicines [1]. One approach for inducing cancer in zebrafish is the xenotransplantation of human cancer cells [2]. Tumor cell behavior in zebrafish xenografts correlates with human cancers, and mice and zebrafish xenografts have similar growth kinetics, histology, and proliferation and apoptosis rates [3,4]. Moreover, zebrafish offer some advantages for drug discovery screening over murine models, such as ease of replication due to large clutch sizes, reduced husbandry expenses and embryo transparency, allowing for in vivo observation of cancer processes [5].

Engraftment success of implanted cancer cells is cell line-dependent, and innate immunity was recently shown to play a key role in this process in zebrafish [6]. Numerous previous studies employing zebrafish xenografts for compound screenings did not provide details regarding the engraftment process but rather focused on comparing control and treatment groups on the final day of experimentation [7]. However, tumor engraftment methods should be validated to ensure the reliability of drug efficacy assessments and to draw reliable conclusions.

Zebrafish tumor xenografts are usually analyzed by imaging techniques such as confocal microscopy, which provides tumor volume measurements, but this process is time-consuming and requires expensive equipment and skilled personnel, thus limiting its use in screening. Fluorescence stereomicroscopes are cost-effective, widely available in laboratories and commonly included in image readers, thereby enabling automatic image acquisition; however, only single focal-plane images are obtained, and some information is thus lacking. Alternative techniques have recently been applied to monitor zebrafish xenografts, such as the quantification of human cells inside xenografted embryos by the qPCR amplification of RNA or gDNA templates [8,9]. These novel strategies could overcome imaging drawbacks and speed up xenograft monitoring to further promote the use of this model in the drug discovery process.

Cancer cells can be implanted at different sites, with the yolk sac being one of the most extensively employed. Although other sites are employed, 52% of the scientific articles about zebrafish xenotransplantation up until 2021 used the yolk sac as the site of implantation (Table S1). In other studies, cells were microinjected into other regions, such as the perivitelline space (PVS; 25.67%) and duct of Cuvier (DoC; 7.75%), and into less common regions, such as the eye, brain and caudal vein (15.50%) [2,10]. After xenotransplantation, embryos are treated with several drugs as single agents or in combination, and drugs are classically administered to fish by dissolving the compound directly in their water because they can absorb solubilized compounds through the skin [5,11]. However, the physicochemical properties of each compound impact their solubility and permeability [12], and the U.S. Food and Drug Administration (FDA) proposed a Biopharmaceutical Classification System (BCS) according to these characteristics. Class I compounds are optimal and have high solubility and permeability [13]. However, new drugs tend to exhibit lower solubility, and only 10–20% belong to class I [14]. Thus, considering that screening campaigns are performed with a large variety of molecules by immersion (many of which have poor solubility and/or permeability), varying uptake into zebrafish causes uncertainty regarding the exact efficient concentrations, and the screening results may thus lead to misleading conclusions, ranging from false negatives to the equivocal prioritization of potential hit compounds [15]. An alternative drug administration strategy is direct inoculation via microinjection, as drugs and molecules were previously administered via injections into the retro-orbital space, brain, yolk sac and otic vesicles [16,17,18,19].

In summary, the use of the zebrafish xenotransplantation model for drug discovery is a complex multifactorial process. Currently, a wide range of approaches is taken, revealing the lack of an experimental consensus in the scientific literature. Herein, we explored the main steps of this procedure to set standard guidelines, which rely on metrics-based assessments, and support reliable screening campaigns. The steps explored using a melanoma cell line were as follows: (1) cancer cell implantation into different sites (pericardial space (PCS), dorsal PVS, ventral PVS and yolk) to identify the best site for tumor engraftment and growth enhancement; (2) establishment of user-independent inclusion criteria to ensure sample homogeneity; (3) determination of tumor engraftment using imaging as a monitoring technique; (4) imaging and qPCR comparisons to identify the most appropriate quantification approach; (5) gDNA and RNA assessment to identify the most suitable material for qPCR; (6) injection site evaluation for compound administration; and (7) comparison of compound administration routes (immersion and intratumoral injection) in efficacy assays using selinexor as an example.

## 2. Materials and Methods

### 2.1. Image Analysis

Imaging was performed by anesthetizing zebrafish embryos, embedding them into a drop of 3% methylcellulose and placing them in Shandon™ 12-well slides (Thermo Scientific, Waltham, MA, USA). Embryos were positioned in the same orientation every time to reduce variability between measurements. Image acquisition was performed with a Cytation 5 image reader with a 4× objective for the brightfield channel with the following parameters: LED intensity = 5, integration time = 63, and gain = 0. The parameters for the RFP channel were as follows; LED intensity = 5, integration time = 860, and gain = 0. The fluorescent tumor area (*TA*) was determined by Gene 5 software, and the threshold was set to 7000 to remove embryo autofluorescence.

### 2.2. RNA/DNA Genetic Extraction and Reverse Transcription

To extract both DNA and RNA from the same sample, a DNA/RNA Omega Bio-Tek kit (Omega Bio-Tek Inc, Norcross, Georgia) was used according to the manufacturer’s instructions. Then, the 260/280 ratio and yield were determined using a SpectroStar Nano spectrophotometer (BMG Labtech, Ortenberg, Germany) and MARS 3.33 software. cDNA from the RNA samples was obtained using Prime Script RT (Takara, Japan) prior to qPCR using a StepOne Plus Real-time PCR system (Applied Biosystems, Waltham, MA, USA).

### 2.3. Primers Quality Test

Quantitative PCR (qPCR) was performed on a StepOne Plus Real-time PCR system (Applied Biosystems, USA). cDNA qPCRs were carried out by adding 100 ng of sample and 10 ng of gDNA at the following PCR cycling parameters: 0.5 min at 95 °C, 0.08 min at 95 °C, and 0.5 min at 60 °C. To evaluate the primer quality, serial dilutions were made, and 40 cycles of qPCR were performed by adding a melting curve step. The following Alu gDNA primers were employed: Fwd-5′-GTC AGG AGA TCG AGA CCA TCC C and Rvs-5′-TCC TGC CTC AGC CTC CCA AG. The hprt1 primers for cDNA samples were as follows: Fwd-5′- TTG CTG ACC TGC TGG ATT AC and Rvs-5′- TAT GTC CCC TGT TGAC TGG T. The primers were considered to be efficient when the standard curve generated had a correlation coefficient (R2) ≥ 0.9, a slope ≥ −3.3, an efficiency between 90% and 120%, and only one peak in the melting curve.

### 2.4. qPCR Standard Curves

To quantify human cancer cells in xenografted zebrafish embryos, standard curves were generated using gDNA and RNA extracted from the same samples. Pools of 10 2-day-old embryos were mixed with different amounts of 888mel mCherry cells (ranging from 1.28 × 10^5^ to 0 cells as negative control). After RNA/DNA extraction, qPCR was performed using AluA primers for gDNA samples and hprt1 primers for RNA samples.

### 2.5. Zebrafish Maintenance and Egg Collection

AB Wild-type zebrafish (*Danio rerio*) were maintained and raised at Ikan Biotech fish facilities according to conditions described previously [20]. Adult fish were obtained from Carolina Biologicals/KIT (USA/Germany) and kept at 27–28.5 °C on a light–dark cycle. Three male and two female fish were kept separately overnight in breeding tanks provided with a grid to prevent egg predation. Spawning was stimulated by the onset of light, and offspring were collected in Petri dishes filled with E3 medium that was prepared according to Cold Spring Harbor protocols [20]. After 24 h of incubation at 28 °C, infertile or dead eggs were removed, the E3 medium was changed, and the viability and presence of malformations were evaluated. Ethical approval to employ zebrafish embryos for up to 4 dpi was obtained from the Universidad de Navarra´s Ethical Committee (056-20).

### 2.6. Cell line and Culture Methods

The melanoma cell line 888mel mCherry was generated at the University of Manchester and grown in DMEM (Biowest, Nuaillé, France) supplemented with 10% FBS Gibco reagent (Life Technologies, Carlsbad, CA, USA), 1% penicillin and streptomycin Gibco reagents (Life Technologies, Carlsbad, CA, USA) and 2.5 mL GlutaMAX Gibco reagent (Life Technologies, Carlsbad, CA, USA). The cells were maintained in a humidified atmosphere at 37 °C and 5% CO_2_ and checked routinely for mycoplasma using a MycoAlertTM Mycoplasma Detection Kit (Lonza, Basel, Switzerland) according to the manufacturer’s instructions.

### 2.7. Xenotransplantation and Injection Site Experiment

Using borosilicate capillaries calibrated using mineral oil and a graticule to inoculate 4 nL, 2-day-old zebrafish embryos anesthetized with 0.4 mg/mL tricaine were placed carefully in an agar plate and microinjected with approximately 1000 cells/4 nL. To identify the site of cancer cell implantation that enhances tumor engraftment and growth, 888mel mCherry cells were transplanted into several zebrafish embryo sites (dorsal PVS, ventral PVS, yolk and PCS), and the experiment was performed in a balanced incomplete block design [21]. Thus, on each microinjection day, the cells were implanted at 2 different locations, becoming a block, and the injection sites were randomly distributed in a 2 by 2 pattern until 3 independent replicates were established for each site. Following injection, the xenografted embryos were collected and left in a Petri dish for 2 h in E3 medium in the dark and at room temperature for recuperation. Embryos were again anesthetized in a Petri dish with tricaine and sorted using a fluorescence microscope (Nikon Europe BV, Amsterdam, The Netherlands). Embryos with cancer cells in the circulation were discarded. Finally, xenografted embryos were incubated for 24 h at 34 °C in the dark in a humidified atmosphere. At 1 dpi, the embryos were anaesthetized and sorted again using a fluorescence microscope, and only embryos with compact tumor masses that were homogeneous in size were selected. For dorsal PVS cell implantation, both embryo orientations were evaluated, as the cells could have implanted into the right or left flank. This orientation was also considered during image acquisition. Discarded embryos were euthanized, and selected embryos were kept in individual 96-well plates (Corning Inc., Corning, NY, USA) and incubated at 34 °C for the rest of the experiment. To compare the qPCR and imaging techniques, pools of 10 xenografted embryos at each location were imaged and kept at −80 °C for gDNA and RNA extraction and qPCR.

### 2.8. Engraftment Definition and Experimental Inclusion Criteria

We defined some criteria to homogenize the experimental data in our sample set. First, the median tumor area (*TA*) at 1-day post-injection (dpi) was calculated for each xenograft embryo experiment, and the threshold was determined using the Formula (1):

(1) $$Inclusion\;threshold\;\left(IT\right)=TA\;median\;at\;1\mathrm{dpi}-1.25\times interquartile\;range\;\left(IQR\right)$$

Later, the data were reanalyzed, and only the embryos that presented a higher *TA* than the inclusion threshold (*IT*) at 1 dpi, were included in the efficacy assessment, which was key for decision making. Experimental replicates were removed from the analysis when more than 25% of the xenografted embryos were discarded according to the inclusion criterion. Xenografted embryos were deemed to be engrafted when they possessed a *TA* on the final day of experimentation that was equal to or higher (≥) than that at 1 dpi. Then, the percentage of zebrafish (*ZF*) embryos with engrafted cells was calculated using the following Formula (2):

(2) $$ZF\;with\;engrafted\;cells\;\%=\frac{nº\;embryos\;with\;tumor\;engraftment\;on\;the\;final\;\mathrm{dpi}}{nº\;of\;total\;living\;embryos\;on\;the\;final\;\mathrm{dpi}}$$

#### 2.8.1. Biodistribution and Dissemination Assay

Alexa FluorTM 488 dextran 10,000 MW (Thermo Fisher Scientific, Waltham, MA, USA) was used for distribution evaluation. A stock solution (10 mg/mL) was prepared according to the manufacturer´s instructions, and borosilicate glass capillaries were then calibrated for microinjection of 4 nL. To assess toxicity and compound leakage, we performed an initial experiment in which the embryos were microinjected into the dorsal PVS, PCS, yolk sac, hindbrain, retro-orbital space and otic vesicle. In the first experiment, images were acquired at different time points using Cytation 5 image reader, and the embryos were kept until 1 dpi. The dorsal PVS and hindbrain locations were discarded, and the experiment was performed once again. Then, the microinjected embryos were fixed with 4% paraformaldehyde at 10-min post-injection (mpi), 60 mpi and 120 mpi. Confocal image acquisition was carried out using Opera imaging device (Perkin Elmer) at the Spanish National Cardiovascular Research Centre (CNIC). Finally, analysis was performed with Fiji software [22]. A macroscript was developed by image analysts to manually draw the areas of interest around the dorsal PVS and PCS. Metrics obtained from image acquisition were: Area = number of pixels from the region of interest; Mean = mean intensity in the region of interest (total accumulation corrected with the number of pixels), Int Den = intensity accumulation in the region of interest. Dextran was quantified as the Mean to correct the fluorescence intensity within the delimited area and reduce the influence of the embryo orientation. Each image was manually segmented 6 independent times, 3 from the same confocal sections and 3 others by independently selecting three other confocal sections. Furthermore, embryo autofluorescence was normalized to that in the control group (non-microinjected embryos) by Z-score correction. The Z-score corrections with values from the negative control group (non-microinjected embryos) were determined as follows (3); where x is measurement, µneg ctrl is the negative control measurement and σneg ctrl, the standard deviation of the negative control:

(3) $$Z\;\mathrm{score}\;\left(x\right)=\frac{x-\;µ\mathrm{neg}\;\mathrm{ctrl}}{\sigma \mathrm{neg}\;\mathrm{ctrl}}\;$$

Finally, intrasubject delineation variability was evaluated using the Dice similarity coefficient and the 3 independent manual segmentations [23]. The Dice similarity coefficient is a commonly used segmentation evaluation metric based on a spatial overlap correspondence. This coefficient ranges from 0 to 1, and a higher value indicates a higher correlation between segmentations. Segmentations were considered successful if the Dice coefficient was higher than 0.60. A total of 131 regions were segmented three times each by the same expert observer. The three repetitions were made from the same confocal section that was selected and fixed prior to delineating the regions of interest. For every triplet of repetitions (S1, S2, S3), three measurements were computed (S1&S2, S1&S3, S2&S3), considering that the Dice similarity coefficient satisfies the commutative property.

#### 2.8.2. Drug

Selinexor (KPT-330) was purchased from SelleckChem (SelleckChem, Houston, TX, USA) in a stock solution of 10 mM in DMSO. This stock was kept according to the datasheet instructions, and the drug was freshly prepared daily prior to experimentation.

#### 2.8.3. In Vitro Cell Viability Assay (MTS)

The CellTiter 96^®^ Aqueous One Solution Cell Proliferation assay (Promega, Madison, WI, USA) was employed to test chemotherapy drugs. Further, 1000 888mel-mCherry cells per well were distributed into 96-well plates and incubated at 37 °C and 5% CO_2_ in a humid environment for 48 h. The drug was added at different concentrations (10 µM, 1 µM and 0.1 µM) to fresh medium, and the cells were incubated for another 48 h under the same conditions. Afterwards, 20 µL of MTS [3-(4,5-dimethylthiazol-2-yl)-5-(3-carboxymethoxyphenyl)-2H-tetrazolium] was added to 200 µL of fresh medium per well, and after 2 h of incubation, the absorbance at 490 nm was measured with a Cytation 5 image reader.

#### 2.8.4. Maximum Tolerated Dose (MTD) Assay

Three-day-old zebrafish embryos were placed into 96-well plates with 200 µL of E3 medium containing serial dilutions of selinexor (5 µM to 0.05 µM). Each experimental group consisted of 8 individuals and a control group with the same DMSO percentage as the maximum drug concentration tested. Embryos were incubated at 34 °C in a humidified atmosphere. Every 24 h, embryo viability was checked; the medium was removed, and the medium was replaced with fresh medium containing the drug or vehicle. The survival rates are represented as Kaplan–Meier curves using GraphPad Prism 8 software.

#### 2.8.5. In Vivo Drug Efficacy Assays

We aimed to perform in vivo efficacy assays using zebrafish embryo xenografts (Figure S1). After cancer cell xenotransplantation, sorting and imaging, engrafted zebrafish embryos were treated with different concentrations of selinexor administered via immersion or direct intratumoral microinjection. Drug at varying concentrations was prepared for injection with different percentages of DMSO, saline solution and 10% Tween 20 (Sigma Aldrich, San Luis, MO, USA) to enable drug solubility. To calculate the drug concentrations, the total volume of zebrafish embryos at 3 dpf (270 nL) was employed [24]. In total, 24 embryos were employed in each experimental group, with each experiment having 3 controls. One control group without implanted cells was used to control the viability of the clutch, while another control group of embryos with xenografted cancer cells implanted in the PCS but without treatment was used to evaluate the transplantation technique and a control group of xenografted embryos with cancer cells implanted into the PCS and treated with the drug vehicle were utilized. Prior to drug microinjection, borosilicate glass capillaries were calibrated as above. Zebrafish embryos were anesthetized with tricaine and placed into agar plates, wherein the drug was administered by intratumoral microinjection into the PCS. Later, the zebrafish embryos were kept in recovery medium for 1 h before being placed individually in 96-well plates in 200 μL of E3 medium at 34 °C in the dark and incubated in a humid environment for 24 h. This drug administration process was carried out for 2 consecutive days until 3 dpi or for 3 consecutive days until 4 dpi. On the final day of the experiment, images were acquired again as previously described. To treat the xenografted embryos with drugs by immersion, sorting and imaging were performed at 1 dpi, and the zebrafish xenografted embryos were placed individually into 96-well plates containing 200 μL of E3 medium supplemented with the dissolved drug. The medium was removed every 24 h and replaced with fresh E3 medium containing the drug or vehicle until the final day of the experiment.

#### 2.8.6. Metrics Definition for Efficacy Assays

The Relative tumor growth (*RTG*) and Difference in tumor growth (*DTG*) employed in the in vivo drug efficacy assays were calculated using the *TA* measured by imaging and by these Equations (4) and (5):

(4) $$RTG\;\left(\%\right)=\frac{TA\;final\;\mathrm{dpi}-TA\;1\mathrm{dpi}}{TA\;1\mathrm{dpi}}\times 100$$

(5) $$DTG\;\left(\%\right)=Tumor\;growth\;vehicle\;\left(\%\right)-Tumor\;growth\;treated\;\left(\%\right)$$

Comparisons in efficacy assays were analyzed by normalizing the *TA* at 4 dpi to that at 1 dpi for each embryo and defining the absolute relative change and relative change as follows (6) and (7):

(6) $$Normalized\;tumor\;area\;\left(NTA\right)=\frac{TA\;at\;4\;\mathrm{dpi}}{TA\;at\;1\;\mathrm{dpi}}$$

(7) Absolute differnce in efficacy=NTA vehicle−NTA treated

An *absolute difference in efficacy* value of 0 indicated no efficacy, as there was no difference between the *NTA* in the xenografted embryos treated with the drug and the *NTA* in the xenografted embryos treated with the vehicle.

#### 2.8.7. Statistical Methods

Analyses were carried out using the statistical software STATA 12 or GraphPad Prism 8. Different points from qPCR standard curves were analyzed by one-way ANOVA using GraphPad Prism 8 software. Survival curves of zebrafish embryos were analyzed using STATA 12 statistical software by the Kaplan–Meier method, and curves from different experimental groups were compared using the log-rank test. Regarding the injection sites assay, significant *TA* differences were measured at different time points with STATA 12 software. The fact that the experiment was designed as a balanced incomplete block was considered in the statistical analysis. A mixed model was employed to treat missing values [25,26]. Thus, a mixed factorial ANOVA was employed and then used to compare groups with the anovalator STATA command. Correlations were statistically analyzed using GraphPad Prism 8 software. First, the normality distribution of the data was analyzed by the Shapiro–Wilks test. Then, correlations were analyzed by the Pearson correlation coefficient for normally distributed data and by the Spearman correlation coefficient for non-normally distributed data. The results of imaging versus qPCR assays that used pools of 10 embryos and the Alexa Fluor 488 dextran dissemination assays were analyzed by STATA 12 software using a factorial 3 × 4 ANOVA followed by multiple 2 by 2 comparisons. Finally, GraphPad Prism 8 was used for the drug efficacy assays. Outliers were removed by the Rout method (Q = 1%), and the normality of the data distribution was evaluated using the Shapiro–Wilks test. Groups were compared by an unpaired *t*-test if the data showed a normal distribution and by the Mann–Whitney test if the data did not. In contrast, to analyze the *TAs* in the same group from 1 dpi to the final day of the experiment, a paired *t*-test was employed for data that showed a normal distribution, and a Wilcoxon matched-pairs signed-rank test was used as the nonparametric method. To analyze the absolute and relative differences among different administration routes, normality was first evaluated as previously described. If the data did not have a normal distribution, the Kruskal–Wallis test with Dunn´s multiple comparison test was performed for comparisons of three groups. In contrast, the differences between two groups were analyzed with the Mann–Whitney test for non-normally distributed data.

## 3. Results

### 3.1. Monitoring Approaches

To quantify *TA* by imaging, we acquired images of individual embryos as detailed in Materials and Methods. Sorting at 1 dpi was performed to select well-injected embryos with homogeneous tumor sizes. Although skilled personnel performed this procedure, it was user-dependent. Thus, we established user-independent inclusion criteria to homogenize the sample set (Section 2).

Then, we evaluated whether qPCR could be used to overcome the previously mentioned limitations of stereomicroscopy. Therefore, we constructed standard curves using RNA and gDNA from a mixture of 10 embryos and a known number of cancer cells to quantify the exact number of 888mel mCherry cells injected into the xenografted embryos. This provided a tool to identify the exact number of cells from a sample of xenografted embryos by inverse interpolation. Once primer efficiency was validated (Figure S2), standard curves for the human housekeeping genes hprt1 (RNA) and AluA (gDNA) from the same samples were obtained (Figure S3). Approximately 1000 cancer cells were to be microinjected into zebrafish embryos for xenotransplantation. Analysis of significant differences between points on the standard curves was performed to establish the appropriate dynamic range for assessing the therapeutic effect of compounds. The RNA standard curve showed more than 1.5 log fold difference between points, meaning that the dynamic range is higher for RNA; and therefore is less sensitive than gDNA (0.75 log fold difference between points) for evaluating the antitumoral effects of potential tested compounds. In summary, although both techniques enabled tumor behavior monitoring in xenografted embryos, gDNA is more reliable than RNA to quantify the exact number of human cells present in xenografted zebrafish embryos.

### 3.2. Injection Site Assay

**Figure 1:**
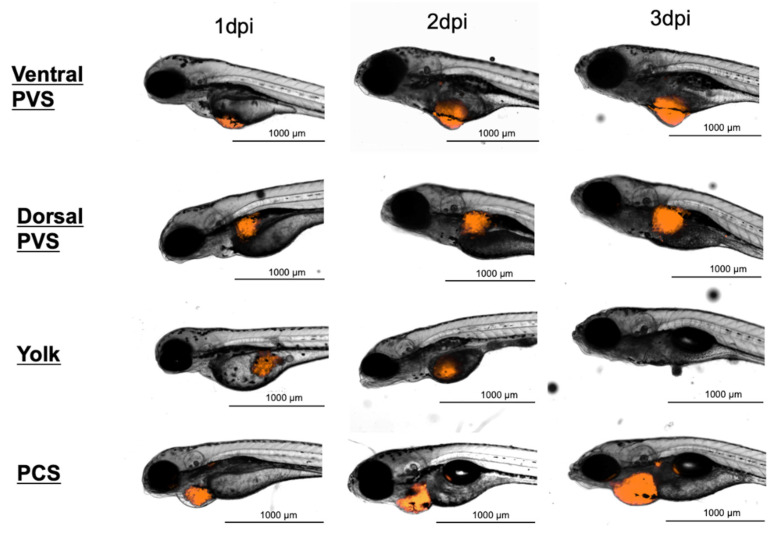
Representative serial images at three time points of zebrafish embryos transplanted at 2-day-old with approximately 1000 888mel mCherry cells at different injection sites. dpi, days post-injection; PVS, perivitelline space; PCS, pericardial space.

**Figure 2:**
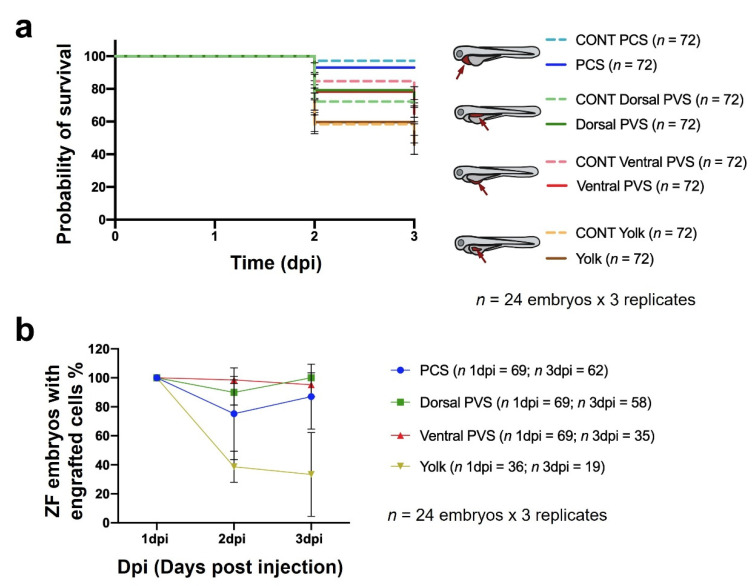
Impacts of different sites of cell implantation on embryo survival (**a**) and the percentage of zebrafish embryos containing engrafted 888mel mCherry cells. (**b**) Controls (CONT) refer to embryos microinjected with only the vehicle in which cells were diluted for microinjection (PBS + 2% PVP 60). The data are represented by Kaplan–Meier survival curves, and the results of statistical analyses for (**a**) are detailed in Table S5. Only zebrafish exhibiting a higher *TA* at 1 dpi than a pre-defined threshold were included in the analysis. Embryos were considered to present engrafted cells when the *TA* at 3 dpi was ≥ than the *TA* at 1 dpi. Results of the statistical analyses for (**b**) are shown in Tables S6.

To elucidate the best site for cancer cell implantation, 888mel mCherry cells were microinjected into four different sites (Figure 1). Embryos microinjected with cells or vehicles into the pericardial space (PCS) showed the highest survival rates at 93.06% and 97.22%, respectively, and those injected into the dorsal perivitelline space (PVS) also exhibited elevated percentages of survival (75% for cell implantation and 68.06% for vehicle injection). Cells microinjected into the ventral PVS led to a survival rate of 64.78%, while vehicle inoculation was 76.39%. However, the lowest survival rates were obtained for cells or vehicles microinjected into the yolk sac (45.83% and 52.78%) (Figure 2a).

**Figure 3:**
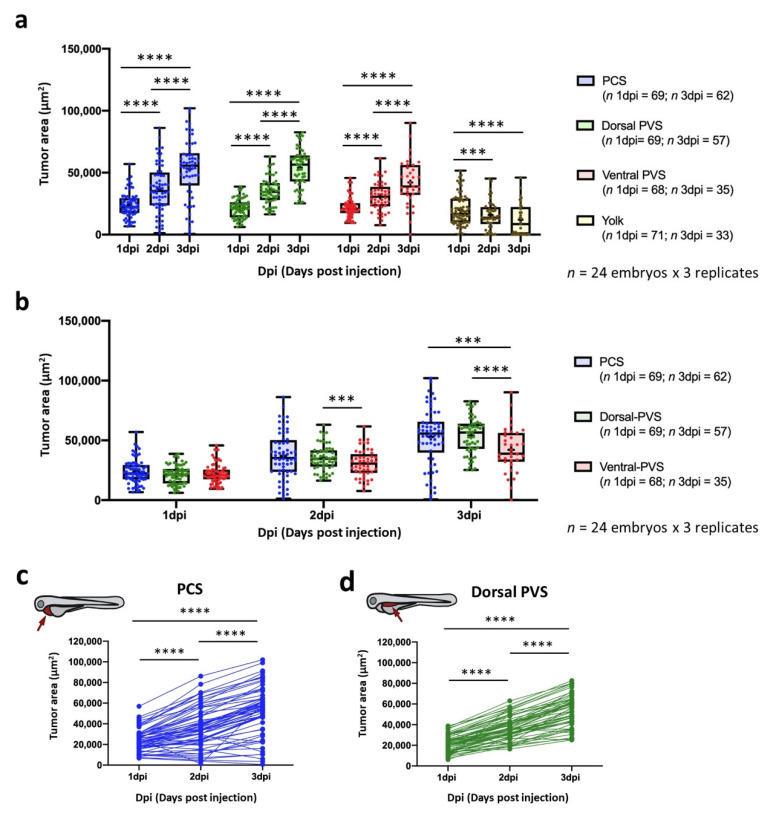
Impact of site of cell implantation on tumor area ascertained by imaging. (**a**) Comparison of the tumor areas of engrafted embryos at three time points grouped by the sites of injection. (**b**) Comparison of the tumor areas of engrafted embryos microinjected into different injection sites grouped by time points. Data are presented as box-leaf plots, where the box indicates IQR, line the median value and leafs the 5–95 percentile range. (**c**) Change in tumor area tracked for individual embryos implanted into the PCS and dorsal PVS. Only zebrafish possessing a tumor area higher than a pre-defined threshold were considered for the analysis (Table S2). Each dot represents an individual embryo. The experiment was designed as a balanced incomplete block assay. Mixed factorial ANOVA was performed with the *anovalator* STATA command (*** = *p*-value < 0.001; **** = *p*-value < 0.0001).

In addition, the xenografted embryos were individually imaged to quantify their *TAs* from 1 dpi to 3 dpi. From this point forward, we applied the previously defined inclusion criteria (Section 2), and consequently, only zebrafish embryos that exhibited a minimum *TA* at 1 dpi were included in the data analysis. The total number of zebrafish embryos originally microinjected and total number of zebrafish embryos considered after inclusion criteria application is shown in Table S2. As also explained above, embryos that displayed an equal or higher (≥) *TA* on the final day of the experiment compared to that *TA* at 1 dpi were considered to exhibit engrafted cells. Then, we quantified the engraftment percentage at each implantation site considering the total number of embryos that were xenografted at 1 dpi and the total number of living embryos with engrafted cells at 3 dpi. The dorsal PVS (100%, SD = 0%) and PCS (93.33%, SD = 11.55%) regions showed the highest engraftment rates, followed by the ventral PVS (87.83%, SD = 11.26%). Conversely, the yolk sac was associated with the lowest rate of engrafted embryos (26.92%; SD = 25.22%) (Figure 2b). After application of the inclusion criterion and individual *TA* monitoring, we detected tumor growth from one day to the next at all injection sites except the yolk sac, where a significant decrease was observed (Figure 3a). Thus, the yolk was identified as the worst site for implantation. Variability shown in *TAs* at 3 dpi after the application of the inclusion criteria results from the individual intrinsic tumor behavior. Therefore, tumor engraftment was not assessed individually but taking into account the pool of xenografted embryos (e.g., 1 dpi vs. 2 dpi, in Figure 3a). Among the remaining locations, significant differences were observed in only the *TAs* of embryos engrafted at the dorsal PVS and ventral PVS regions at 2 dpi (*p*-value = 0.006) (Figure 2b), and the differences at 3 dpi were even more marked. The numbers of embryos engrafted in the PCS and dorsal PVS regions were not significantly different (*p*-value = 0.114) but were different from that in the ventral PVS (*p*-value = 0.001; *p*-value < 0.001). Furthermore, at 3 dpi, the cells implanted into the dorsal PVS and PCS showed the highest *TAs* and RTG from 1 dpi to 3 dpi (dorsal PVS 172.77%, SD = 82.18% and PCS 122.39%, SD = 77.26%). In contrast, cells injected into the ventral PVS had an RTG of 108.32% (SD = 98.89%) (Figure 3b). The *TA* tendencies of individual xenografted embryos in the PCS and dorsal PVS are shown in Figure 3c (and for the remaining injection sites in Figure S4). In summary, we considered the PCS to be the best site for 888mel mCherry cell implantation, as it was one of the two best sites that enhanced tumor growth and allowed the highest survival of xenografted embryos.

**Figure 4:**
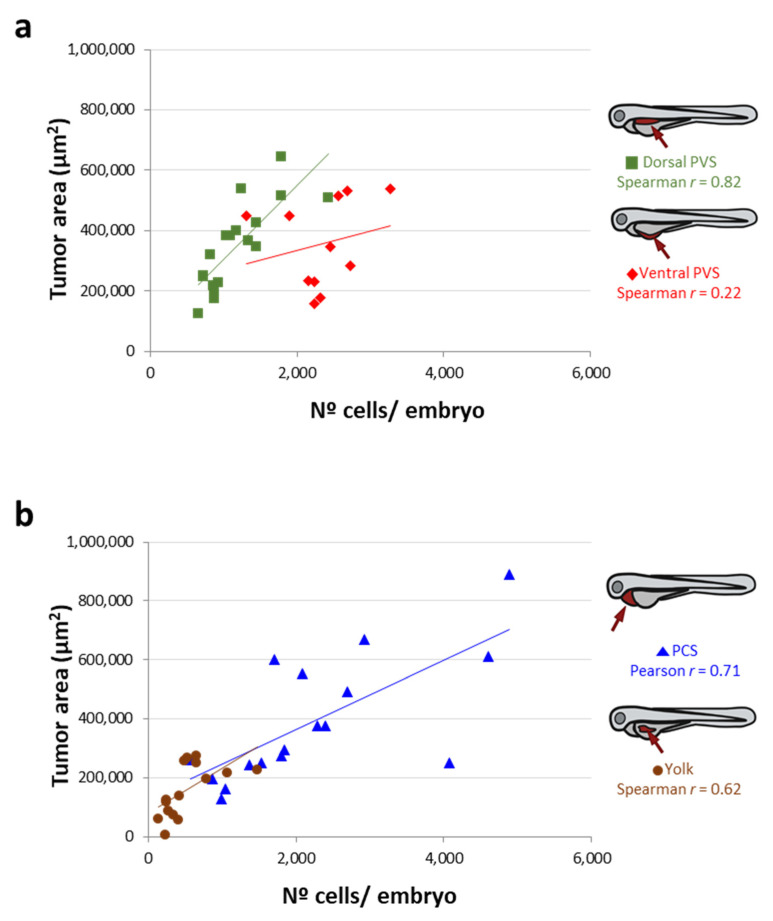
Correlation between the tumor area measured by imaging and the n° of cells/embryo quantified by qPCR (after an inverse interpolation using standard curves) according to each site of injection. (**a**) Correlation between the tumor area (from the 10 embryos that constitute a pool) and the n° of cells determined using gDNA (human Alu sequences) as the PCR template for 888mel mcherry cells implanted into the dorsal or ventral PVS. (**b**) As for (**a**) but cells implanted into the PCS or yolk sac.

To compare tumor behavior monitoring techniques, the same pools of 10 engrafted embryos were analyzed by imaging and qPCR using RNA or gDNA as the template. We determined the correlation between the number of 888mel mCherry cells quantified by qPCR (and the inverse interpolation on the standard curves) with the *TA* measured by imaging of each pool. Using gDNA, the dorsal PVS injection site yielded the best correlation between both monitoring techniques (Spearman r = 0.80), followed by the PCS (Pearson r = 0.71). In contrast, when cells were implanted into the ventral PVS (Pearson r = 0.22) and yolk sac (Spearman r = 0.62), the correlations were lower (Figure 4). In general, according to Spearman/Pearson analyses, the use of RNA rather than gDNA as the template for qPCR leads to worse correlations (Figure S5). In summary, these results correspond with those previously obtained from the standard curves. gDNA was better than RNA for the qPCR tracking of cancer cells. Furthermore, the most accurate technique for monitoring tumor behavior was shown to be dependent on the site of cancer cell implantation (Figure S6). Cancer cells injected into the PCS or dorsal PVS were correctly monitored by both techniques. In contrast, the readouts of cells implanted into the ventral PVS were flawed when acquired by imaging techniques other than confocal microscopy. Thus, in this particular scenario (implantation in the ventral PVS), qPCR might be the most appropriate method for monitoring tumor growth.

### 3.3. Compound Administration and Biodistribution Assay

**Figure 5:**
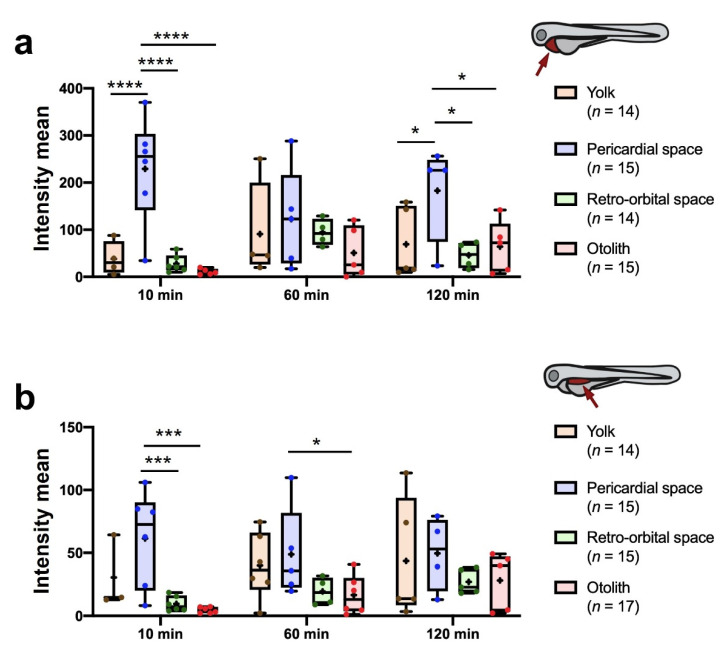
Quantitation of fluorescence intensity in the PCS (**a**) and dorsal PVS (**b**) at time points following inoculation of 4 nL of 10 mg/mL Alexa Fluor 488 dextran at the indicated sites (see supplementary methods for calculation of the relative intensity). Data are presented as box-leaf plots, where the box indicates IQR, line the median value and leafs the 5–95 percentile range. Statistical analysis was performed by 3 × 4 factorial ANOVA followed by multiple comparisons (* = *p*-value < 0.05; *** = *p*-value < 0.001; **** = *p*-value < 0.0001).

We aimed to identify the best site for the inoculation of test compounds that allows for a better biodistribution to the PCS and dorsal PVS (previously determined to be the best locations for cell implantation). To track the administered compound, we used Alexa Fluor 488 dextran, which was inoculated into six different embryo locations (Figure S7). Prior experiments showed that this compound leaked from the embryo when it was injected into the dorsal PVS, as no fluorescence signal was observed, and the embryo died at 1 dpi when it was delivered into the hindbrain. In contrast, it remained inside the body when it was inoculated into the remaining locations, and the embryos survived until 1 dpi. Hence, the yolk and dorsal PVS were discarded, and the experiment was carried out again to monitor the fluorescent signal in the dorsal PVS and PCS by manual segmentation (Figure 5). The results were comparable, as the same images were employed for both quantifications. Although the relative intensities of this compound were higher in the PCS segments, the relative intensity was higher after inoculation into PCS and yolk from 10 mpi to 120 mpi in both regions. The Dice similarity coefficient, calculated to evaluate the intra-user delineation variability of both manual ROI selections (PVS and PCS), was higher in the PCS (mean = 0.81; SD = 0.08) than in the PVS (mean = 0.60; SD = 0.16) (Figure S8). However, it was acceptable in both regions, indicating that the manual segmentation was performed consistently. In summary, our results identified PCS as the best location for compound inoculation because it allows higher rates of dissemination throughout the embryo body to reach the PCS and dorsal PVS.

### 3.4. In Vivo Efficacy Assays

As previously described, compound administration is a crucial step in efficacy assays. Screening compounds have different or unknown physicochemical properties that can influence solubility and/or permeability, two factors that are key for biopharmaceutical classification. Because class I compounds are optimal (high permeability and solubility) according to the BCS [26,27,28], we selected a class II compound, selinexor, which is practically insoluble in water, to reproduce a real scenario and to reduce false negatives [29,30]. First, the in vitro antitumoral effect of selinexor on 888mel mCherry cells was assessed with a cell viability assay which revealed a 61.9% reduction in viability at 1 µM (Figure S9). Then, we performed drug efficacy assays of xenografted embryos in which selinexor was administered by immersion or microinjection. Tumor engraftment was verified in every assay by observing *TA* growth from 1 dpi to 3 dpi in the control group (vehicle-treated) and calculating the RTGs (values shown in the figures). To this effect, 6.1 ng of selinexor (50 µM) or vehicle was inoculated into xenografted zebrafish embryos by intratumoral injection on two consecutive days from 1 dpi to 3 dpi. At 3 dpi, the *TAs* of embryos treated with selinexor were significantly lower than those of embryos treated with the vehicle (*p*-value = 0.0206) (Figure S10). The detected DTG percentage between the two groups was 63.7%. These results showed that tumor establishment can be accomplished at 3 dpi, that drug administration by microinjection is innocuous, and that the in vivo efficacy of selinexor can be detected by imaging techniques.

**Figure 6:**
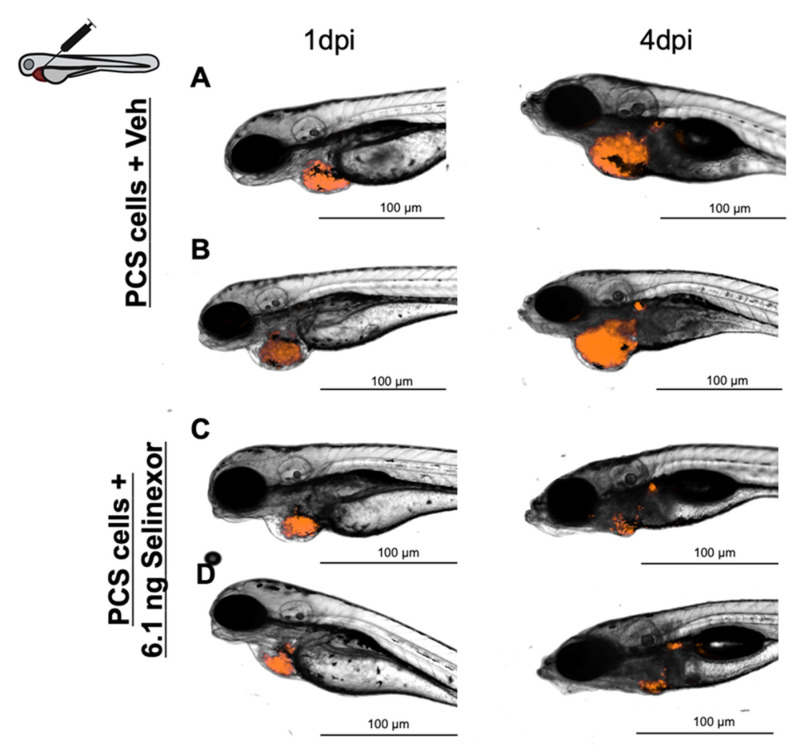
Representative images of embryos engrafted with ~1000 888mel mCherry cells into the PCS and administered 6.1 ng Selinexor by intratumoral injection (**A**,**B**) or vehicle (56.25% DMSO + 10% Tween 20 + saline solution) (**C**,**D**).

**Figure 7:**
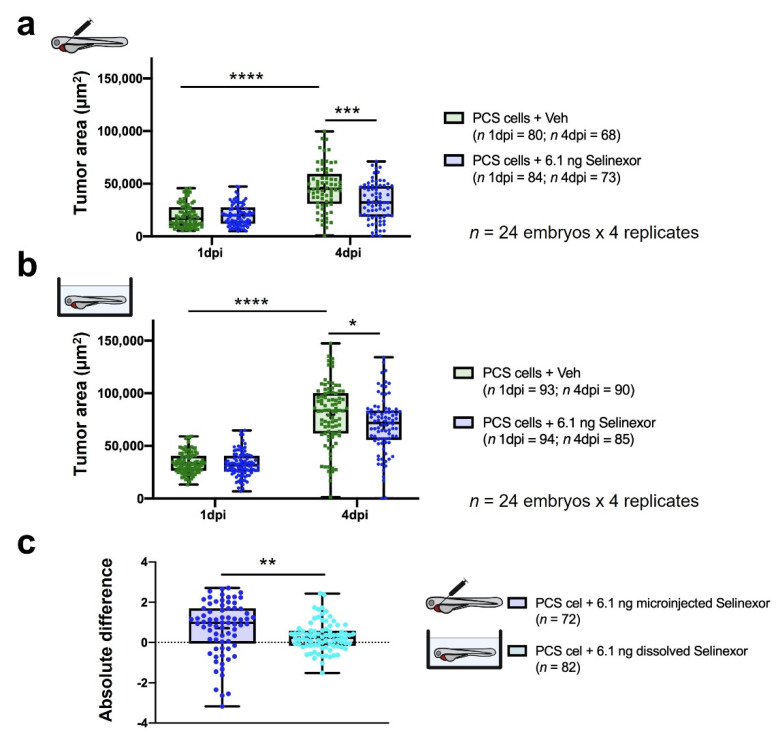
In vivo efficacy of 6.1 ng of selinexor against PCS xenografts comprising 888mel mCherry cells contrasting direct intratumoral injection vs. immersion. (**a**) *Tumor areas* (*TA*) of engrafted embryos treated with selinexor or vehicle (56.25% DMSO + 10% Tween 20 + saline solution) administered by intratumoral microinjection. (**b**) As (a) but treated with selinexor or vehicle (E3 medium + 0.014% DMSO) administered by immersion. (**c**) Absolute difference in the efficacy of 6.1 ng of selinexor administered by intratumoral injection or immersion between treatment and control groups determined for embryos that survived until 4 dpi. Only zebrafish that showed a tumor area higher than a pre-defined threshold were considered for the analyses. Data are presented as box-leaf plots, where the box indicates IQR, line the median value and leafs the 5–95 percentile range. Each dot represents an individual embryo. To determine significance, an unpaired *t*-test or the Wilcoxon matched-pairs signed rank test was performed. For absolute differences, data were analyzed by a Mann–Whitney test. (* = *p*-value < 0.05; ** = *p*-value < 0.01; *** = *p*-value < 0.001; **** = *p*-value < 0.0001).

We tried to increase the DTG between the experimental groups (tumor growth window) to increase the sensitivity window and thereby yield accurate conclusions. Hence, selinexor was microinjected on three consecutive days, extending the assay to 4 dpi (Figure 6). DTG percentage between both experimental groups was higher than initial results, 77.11%. versus 63.7% (4 dpi and 3 dpi respectively) Furthermore, larger differences at 4 dpi were detected in the *TA* after treatment extension (*p*-value = 0.0001 versus *p*-value = 0.0206 at 3 dpi) (Figure 7a). We used 24 embryos per experimental group in the efficacy assays and we performed at least 3 independent replicates. However, to evaluate the minimum number of replicates required, we performed a statistical analysis: 24 embryos per 2 replicates versus 24 embryos per 3 replicates (Tables S3 and S4). These results showed that 6.1 ng Selinexor (lowest concentration tested) by intra-tumoral microinjection until 3 dpi, required at least 3 replicates to detect significant differences. However, extending the drug administration until 4 dpi 2 replicates was enough to observe it. Then, tumor engraftment was assessed after dissolving the same amount of selinexor into the fish water. Although the *TA* measured at 4 dpi in drug-treated embryos was lower than that in vehicle-treated embryos (*p*-value = 0.0217), the DTG was lower (12.3%) between these experimental groups (Figure 7b). However, the efficacy was inferior to that obtained after drug microinjection. Vehicle-treated embryos by microinjection showed an RTG of 177.47% (SD = 138.08%) and 142.89% (SD = 72.56%) by immersion, showing tumor engraftment. To compare both administration approaches, the absolute differences in efficacy were calculated. Considering 0 to indicate no drug efficacy (Materials and Methods), we obtained absolute differences in efficacy of 0.70 and 0.29 after microinjection and immersion, respectively (Figure 7c).

**Figure 8:**
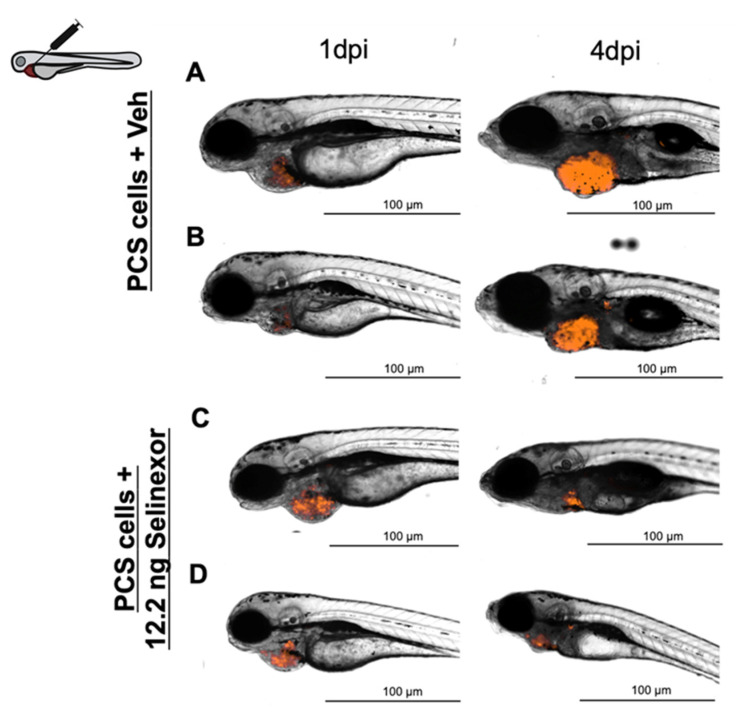
Representative images of embryos engrafted with ~1000 888mel mCherry cells into the PCS and administered 12.2 ng Selinexor by intratumoral injection (**A**,**B**) or vehicle (67.5% DMSO + 10% Tween 20 + saline solution) (**C**,**D**).

**Figure 9:**
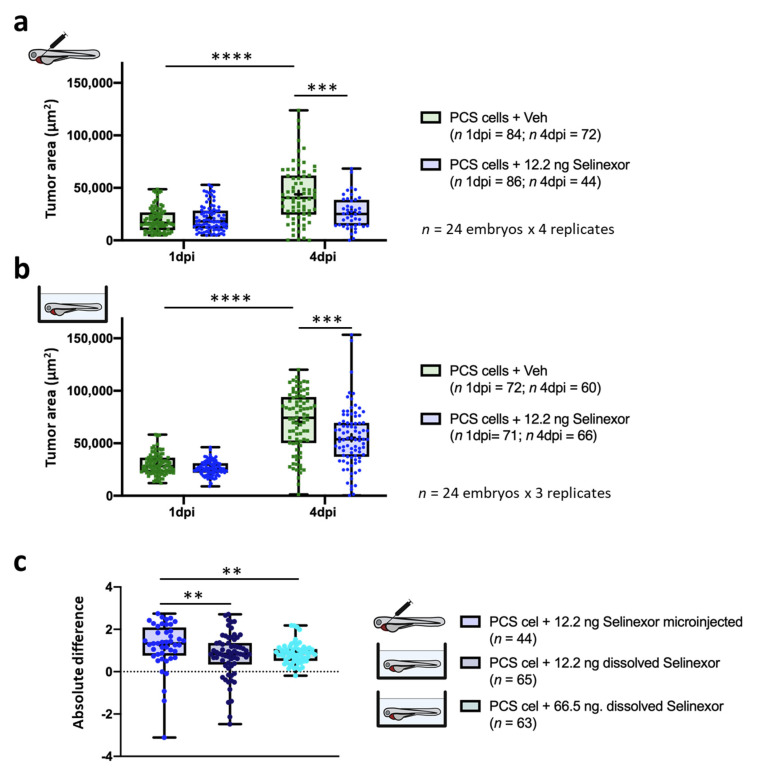
In vivo efficacy of 12.2 ng of selinexor against PCS xenografts comprising 888mel mCherry cells contrasting direct intratumoral injection vs. immersion. (**a**) *Tumor areas* (*TA*) of engrafted embryos treated with selinexor or vehicle (67.5% DMSO + 10% Tween 20 + saline solution) administered by intratumoral microinjection. (**b**) As (a) but treated with selinexor or vehicle (E3 medium + 0.01% DMSO) administered by immersion. (**c**) Absolute difference in the efficacy of 12.2 ng of selinexor administered by intratumoral injection or immersion and of 66.5 ng of selinexor in solution between treatment and control groups determined for embryos that survived until 4 dpi. Only zebrafish that showed a tumor area higher than a pre-defined threshold were considered for the analyses. Data are presented as box-leaf plots, where the box indicates IQR, line the median value and leafs the 5–95 percentile range. Each dot represents an individual embryo. To determine significance, a Mann–Whitney test, paired *t*-test or Wilcoxon matched-pairs signed rank test was performed. For absolute differences, data were analysed by a Kruskal–Wallis test with Dunn’s multiple comparisons test (** = *p*-value < 0.01; *** = *p*-value < 0.001; **** = *p*-value < 0.0001).

To determine whether the response was dose-dependent, we repeated the same experiments by increasing the selinexor concentration to 12.2 ng (100 µM) (Figure 8). At 4 dpi, the drug-treated embryos exhibited significantly lower *TAs* than the control group embryos (*p*-value = 0.0002). Furthermore, the DTG between the experimental groups was 122.46% (Figure 9a). At 4 dpi, the zebrafish embryos treated with the same quantity of drug administered via immersion (0.137 µM) exhibited significantly lower *TAs* than the control embryos (*p*-value = 0.0002) and showed a DTG of 78.1% (Figure 9b). The MTD of selinexor was determined to be 66.5 ng (0.75 µM), and the efficacy assay was carried out (Figures S11 and S12). The absolute differences in selinexor efficacy between the control and treatment groups after microinjection or immersion were 1.22 and 0.77, respectively, and the absolute difference in efficacy after treatment with 66.5 ng of selinexor in solution was determined to be 0.86 (Figure 9c). Thus, selinexor showed a dose–response and a higher antitumoral effect when administered by microinjection.

The treatment impacts on xenograft embryo survival were also analyzed. Embryos treated with 6.1 ng of selinexor exhibited high survival rates regardless of the delivery route (dissolved in media or injected) or the number of administrations; in fact, the survival rate remained above 85% in all of these scenarios (Figures S13 and S14). These results revealed that drug treatment by microinjection was not harmful. Specifically, embryos treated in this manner showed high survival rates, and the rates were decreased only by drug toxicity caused by higher compound concentrations inside the embryo after injection.

## 4. Discussion

Currently, there is no consensus in the scientific literature regarding the compound screening process employing the zebrafish embryo xenograft model. Herein, we aimed to explore the following steps of the procedure to set out a systematic roadmap for drug discovery:

*Experimental criteria and metrics.* To homogenize the sample set at 1dpi and select appropriate embryos for efficacy assessment, we propose a user-independent inclusion criterion to be applied in xenograft imaging monitoring. Thus, inclusion criteria were defined based on the inclusion threshold (IT) assay-dependent metric (Materials and Methods). According to this standard, only xenografted embryos with a *TA* on the final day of the experiment higher than the assay-dependent established IT were considered for the analysis. We also defined other metrics (RTG and DTG) for tumor growth evaluation and comparison between experimental groups in efficacy assays.

*Tumor engraftment assessment.* Verification of tumor engraftment provides certainty that the implanted cancer cells remain inside the zebrafish embryos and guarantees the correct interpretation of the obtained results. We highlight the importance of ensuring engraftment in xenotransplantation, especially in drug efficacy assays. Xenografted embryos were considered to contain engrafted cells if the *TA* at the end of the experiment was equal to or higher (≥) than the *TA* at 1 dpi.

*Monitoring*. Our results showed that human cancer cell xenotransplantation into zebrafish embryos can be detected and accurately quantified using both RNA and gDNA qPCR, consistent with previous studies [8,9]. However, the gDNA was more stable and easier to extract and exhibited higher homogeneity and sensitivity, as described in the Results section, than the RNA according to the statistical outcomes as determined by the standard curves and qPCR-imaging correlations. This outcome coincides with that of Rainero et al., who proved that gDNA qPCR was statistically more reliable than mRNA analysis for detecting leukemic cells [31]. According to our results, qPCR is more accurate than imaging for detecting implanted cells in areas in which three dimensions play a critical role, e.g., the ventral PVS. Thus, in this case, gDNA qPCR can overcome the lack of information yielded by steromicroscopy. However, according to our results qPCR resulted in a less sensitive technique to monitor tumors in xenografted embryos. This is due to the fact that this technique is not able to detect the tumor growth of an individual zebrafish and therefore, pools of embryos are required, which thereby reduces the sample size. Thus, fluorescence imaging has higher statistical power, as the analysis is performed on individual embryos, which also provides detailed tumor growth tracking (Figure 3c). Moreover, as described below, the PCS was herein identified as the best cancer cell implantation site, which was accurately monitored by both techniques. Thus, fluorescence imaging was herein selected as the technique for monitoring tumor growth.

*Site of cancer cell implantation.* One of the most important factors affecting engraftment potential is the site of cell implantation [32]. The yolk sac has traditionally been the most employed location because it provides a larger site for housing transplanted cells [10] (Supplementary Table S1); however, we herein demonstrated the yolk to be the worst site for implantation, as it had the lowest rates of survival, tumor engraftment and growth. This low rate (54.83%) may have been due to microinjection causing some complications, leading to the spillage of cancer cells or yolk content [33]. Furthermore, this procedure could be hazardous due to the proximity of the injection site to the DoC, the common cardinal vein [34]. Furthermore, only 26.92% of the xenografted embryos in the yolk contained engrafted cells at 3 dpi, and the *TA* measured by imaging was decreased in only this experimental group (32.79%). Previous studies compared the yolk sac with other locations and yielded similar results [3,35]. According to our results, the dorsal PVS had the largest DTG percentage from 1 dpi to 3 dpi (172.77% of tumor growth). However, this region was not selected because (i) it requires time-consuming sorting, which should be carefully performed (Materials and Methods), and (ii) it has a lower embryo survival rate (75%) than the PCS. Hence, we identified the PCS as the best location for cancer cell xenotransplantation, as it exhibited the highest embryo viability (93.06%) and high *TA* growth (122.39%) as quantified by imaging.

*Drug administration routes.* Compound screenings in zebrafish embryos have traditionally been performed by immersion. However, compound uptake depends on the physicochemical properties of each molecule [12]; as previously described, this factor may cause misleading conclusions [15]. To overcome the limitations of this strategy, we explored compound microinjection. Using fluorescent dextran, we confirmed that no leakage occurred at the injection site, thereby overcoming the described limitations of this approach [36]. The relative amounts of this compound in the dorsal PVS and PCS (best regions for cell implantation) were higher after inoculation into the PCS, which provides a closed space to contain liquid and is separated from the embryonic circulation by only the cell layer that constitutes the heart. Some previous studies applied PCS injection as a strategy to deliver drugs into the embryo bloodstream [37].

*Drug efficacy assays*. We selected selinexor (class II, insoluble compound), which has antitumoral activity against melanoma, for the in vivo efficacy assays [29,30,38]. The efficacy of selinexor in zebrafish embryos containing xenografted melanoma cells administered in the PCS displayed a dose-dependent response, as the effect of the drug was enhanced by extending the experiment to 4 dpi or by increasing the drug concentration. This extension of the experimental setup requires ethical approval but provides a clear added value of a higher tumor growth window; thus, higher sensitivity and more accurate conclusions are ideal for efficacy assessment and decision making, precluding false negatives and/or misleading compound prioritizations. In addition, the same amount of selinexor showed higher antitumoral efficacy when it was delivered by direct intratumoral inoculation rather than by immersion (absolute difference of 1.22 versus 0.77 for embryos treated with selinexor (12.2 ng) and vehicle, respectively).

Our results suggest that microinjection is a reliable and safe approach to test the antitumoral efficacy of any compound; and thus, this proposed roadmap might lead to a general approach to reliably screen libraries of molecules with unknown physicochemical properties. It may be used to screen not only BSC class I compounds, which have optimal permeability and solubility, but also those from BSC classes II-IV, thereby reducing false negatives and yielding more accurate efficacy metrics.

## 5. Conclusions

**Figure 10:**
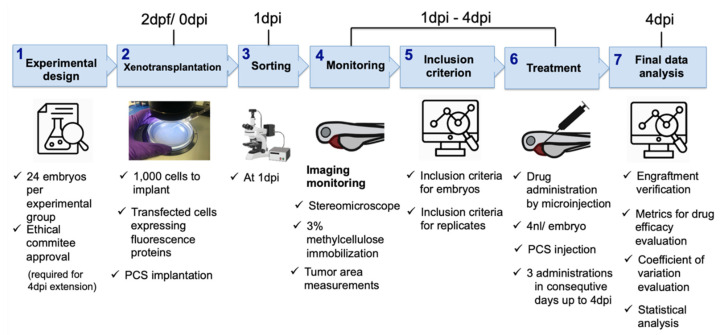
Systematic roadmap for establishing zebrafish embryo xenotransplantation drug efficacy assays.

We herein explored the significant steps in the embryo xenograft assay using melanoma 888mel mCherry cells and selinexor, and we propose the following protocol as a guideline for the standardisation of a zebrafish cancer model in the drug discovery process (Figure 10):

(1) Experimental design. According to our results, twenty-four embryos per experimental group and three independent replicates were enough to see statistically significant results on the final day of the experiment and increased sensitivity to detect smaller antitumoral effects precluding false negatives. Our experimental setup was extended to 4 dpi, which had a clear added value and had a statistically significant impact on the efficacy assessment window and the DTG metric; therefore, ethical approval was required.
(2) Cancer cell xenotransplantation. A total of 1000 cells was sufficient to detect tumor engraftment by imaging, and our results discourage the use of the yolk as the site of implantation. Conversely, the PCS was identified as the best site, as it yielded higher rates of cancer cell engraftment and was less harmful than the other sites, as determined by the engrafted embryos showing higher survival rates.
(3) Sorting. At 1 dpi, sorting should be carefully performed using a fluorescence stereomicroscope to select properly microinjected embryos that exhibit cells at only the correct location and homogeneous tumor masses.
(4) Tumor monitoring. As reported above, gDNA qPCR is a less time-consuming technique that provides some advantages for only ventral PVS implantation. However, due to its detrimental impact on statistical power (requires pools of embryos) and due to the PCS being selected as the site of tumor implantation, fluorescence imaging is the proposed tumor monitoring technique.
(5) User-independent inclusion criteria. We aimed to propose a systematic screening roadmap, including a user-independent decision-making process, to minimize variability and maximize reproducibility. Therefore, we defined assay-based user-independent criteria, including the inclusion threshold (IT), to the efficacy assessment of only embryos that presented with a *TA* at 1 dpi that was higher than the IT, which was key for decision making.
(6) Treatment. According to our results, compound administration by direct intratumoral inoculation is the best approach to treat engrafted embryos and for reliable efficacy assessments. As the experimental setup was extended to 4 dpi, compounds were administered for three consecutive days.
(7) Final data analysis. Only embryos meeting the inclusion criteria (IT) were considered for efficacy assessment and decision making.

The proposed protocol is a systematic roadmap for drug discovery screening using the zebrafish xenograft cancer model to maximize its reliability and reproducibility. However, some challenges remain to be addressed. (a) The microinjection technique is an arduous and time-consuming process that requires trained staff. Microinjection automation is currently being investigated, and this accomplishment could involve the harnessing of this model in high-throughput screening campaigns. (b) Due to the small size of embryos, analytical methods and equipment with high sensitivity to perform pharmacokinetic and pharmacodynamics analyses could provide accurate data regarding the in vivo effects of the screened compounds. Furthermore, more stable cell lines (including tumor cells from patients) and compounds from other BSC classes should be investigated; these studies are currently ongoing in our laboratory.

This study aims to be a stepping stone to bring the versatility of zebrafish embryos to drug screening for cancer and establish zebrafish embryos as a valuable alternative to mice models.

## Data Availability

The data presented in this study are available in the manuscript and Supplementary Materials. Any further information is available from the authors.

## Supplementary Materials

The following are available online at https://www.mdpi.com/article/10.3390/cancers13153705/s1; Figure S1: Xenograft assay with drug treatment administrated by intra- tumoral microinjection or immersion until to 4 dpi; Figure S2: Human primer efficiency tests., Figure S3: qPCR standard curves of 888mel mcherry cells; Figure S4: Tumor area in engrafted embryos with cells implanted into ventral PVS and yolk sac; Figure S5: Correlation between imaging and RNA qPCR according to each site of injection; Figure S6: Summary of the best technique to employ in xenograft monitoring depending on the site of cancer cell implantation; Figure S7: Representative images Alexa Fluor 488 dextran biodistribution assay; Figure S8: Dice coefficient calculated from manually segmentation in the biodistribution assay; Figure S9: MTS assay; Figure S10: 6.1 ng of injected Selinexor efficacy in xenografted embryos until 3 dpi; Figure S11: MTD assay; Figure S12: 66.5 ng of dissolved selinexor in xenografted embryos until 4 dpi; Figure S13: 6.1 ng of selinexor administered by different routes on the survival of engrafted embryos until 3 dpi; Figure S14: Impact of 6.1 ng Selinexor treatment by different administration routes on the survival of engrafted embryos over 3 consecutive days up to 4 dpi; Table S1: Reported studies of zebrafish xenografts assays; Table S2: Evaluation of the minimum number of replicates required for 3 dpi experiments; Table S3: Evaluation of the minimum number of replicates required for 4 dpi experiments; Table S4: Statistical analyses of Kaplan–Meier curves from the injection site experiment; Table S5; Statistical analyses of percentage embryo engraftment from the injection site experiment. Table S6, Statistical analyses of percentage embryo engraftment of cancer cells from each implantation site performed by one-way ANOVA (* = *p*-value < 0.05; ** = *p*-value < 0.01; *** = *p*-value < 0.001; **** = *p*-value < 0.0001).
