# Supplementary material for: Systematic Roadmap for Cancer Drug Screening Using Zebrafish Embryo Xenograft Cancer Models: Melanoma Cell Line as a Case Study
Source: Cancers (Basel). 2021 Jul 23;13(15):3705. doi: 10.3390/cancers13153705 (PMC8345186; doi:10.3390/cancers13153705)
Supplement: Supplementary file 1 [file cancers-13-03705-s001.zip › cancers-1287815-supplementary.pdf]

Systematic roadmap for cancer drug screening using zebrafish embryo xenograft cancer models: melanoma cell line as a case study

Patricia Letrado [1,2], Holly Mole [3], María Montoya [4], Irene Palacios [4], Jorge Barriuso [3,5], Adam Hurlstone [6], Roberto Díez-Martínez [1,*], Julen Oyarzabal [2,*]

[1] Ikan Biotech SL, Centro Europeo de Empresas e Innovación de Navarra (CEIN), Noain 31110, Spain.
[2] Small Molecule Discovery Platform, Molecular Therapeutics Program, Center for Applied Medical Research (CIMA), University of Navarra, Pamplona 31008, Spain.
[3] Division of Cancer Sciences, School of Medical Sciences, The University of Manchester, Manchester, M13 9PL, United Kingdom
[4] Cellomics Unit, Spanish National Center for Cardiovascular Research (CNIC), Madrid 28029, Spain.
[5] The Christie NHS Foundation Trust, Manchester M20 4BX, United Kingdom.
[6]. Division of Infection, Immunology and Respiratory Medicine, School of Biological Science, The University of Manchester, Manchester M13 9PT, United Kingdom.

E-mail addresses: patricia.letrado@ikanbiotech.com and julenoyarzabal@external.unav.es

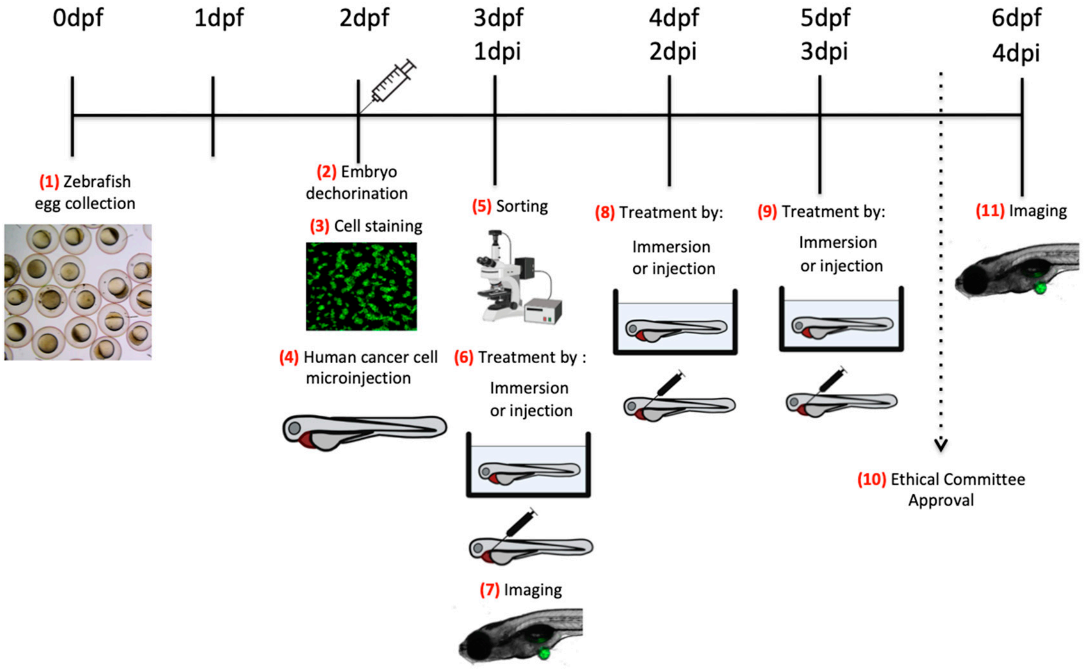

Figure S1. Xenograft assay with drug treatment administrated by intra- tumoral microinjection or immersion 3 consecutive days up to 4 dpi.

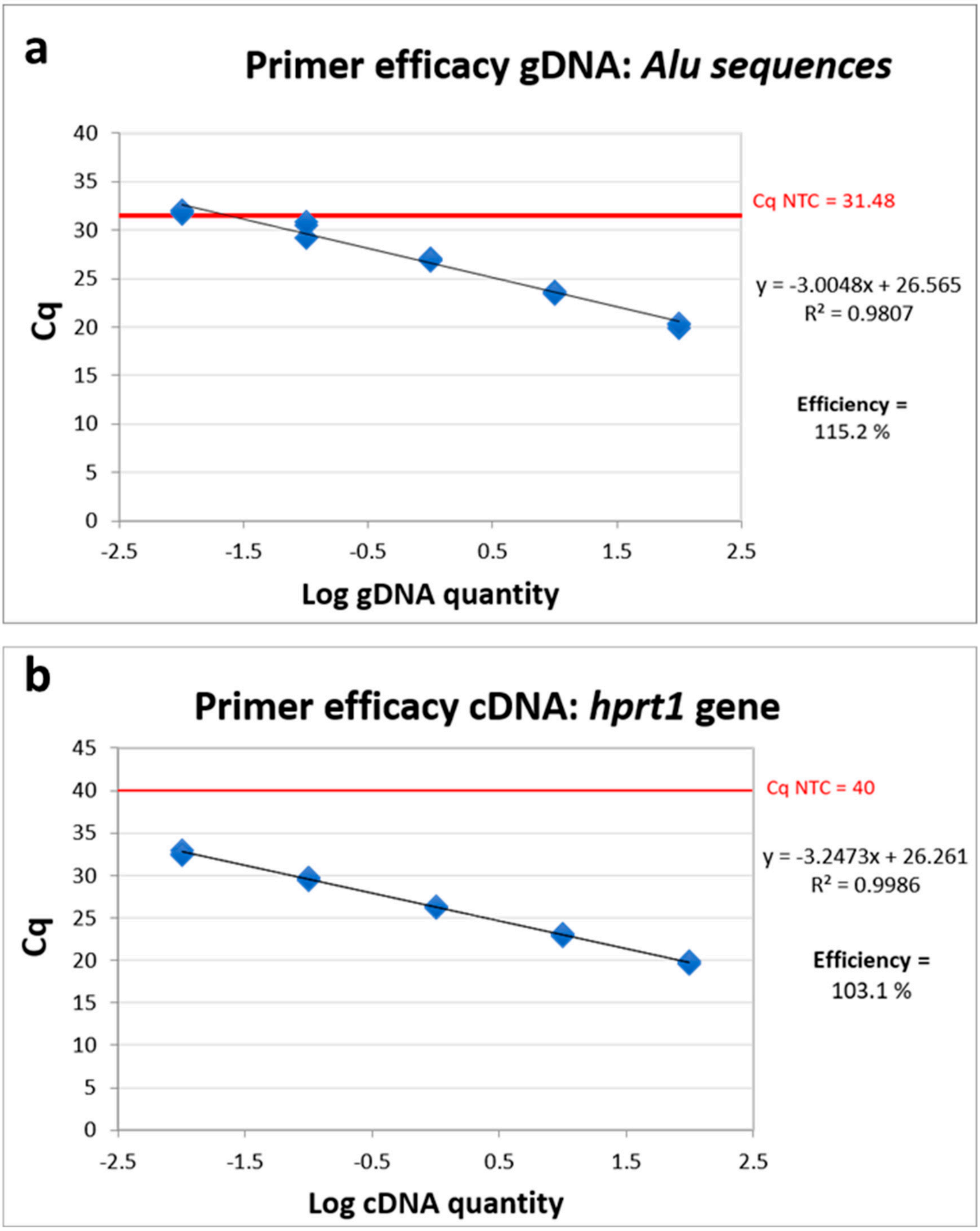

Figure S2. Human primer efficiency tests. **(a)** AluA primers tested on gDNA. (b) hprt1 primers tested on cDNA. NTC is negative control in which only qPCR master mix is added without template material.

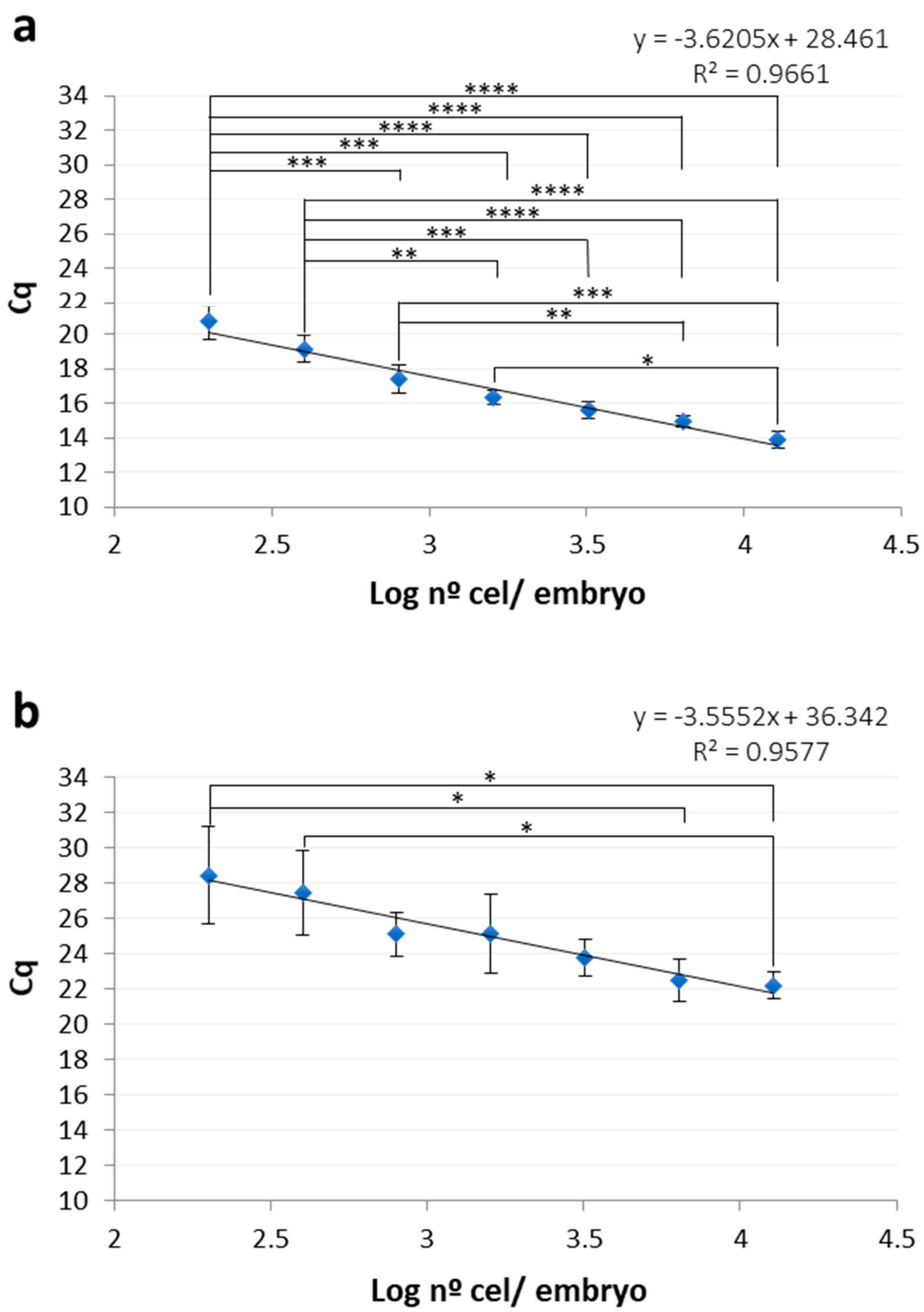

Figure S3. Standard curves obtained by mixing 10 embryos with different numbers of 888mel mCherry cells and performing qPCR. **(a)** Standard curve for AluA primers. **(b)** Standard curve for hprt1 primers. Error bars represent SD and statistical analysis was performed by one-way ANOVA (* = p-value < 0.05; ** = p-value < 0.01; *** = p-value < 0.001; **** = p-value < 0.0001).

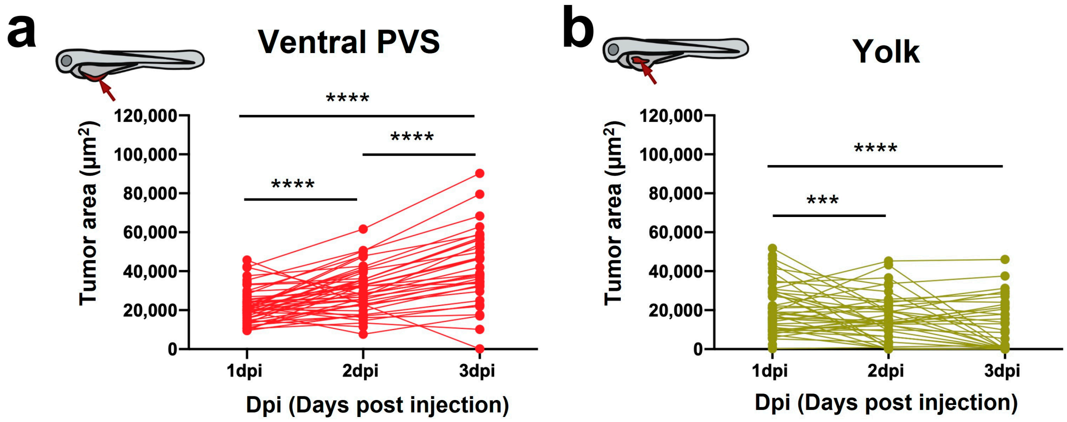

Figure S4. Tumor area in engrafted embryos with 888mel mCherry cells implanted at different injection sites. **(a)** Tumor area tracked in individual embryos engrafted in ventral PVS. **(b)** As for (a) but embryos engrafted in the yolk sac. Only engrafted embryos displaying a tumor area higher than the pre-defined threshold were included for the analysis. Each dot represents an individual engrafted embryo. Statistical significance evaluated using Mann-Whitney and Wilcoxon matched-pairs signed rank tests (*** = p-value < 0.001; **** = p-value < 0.0001).

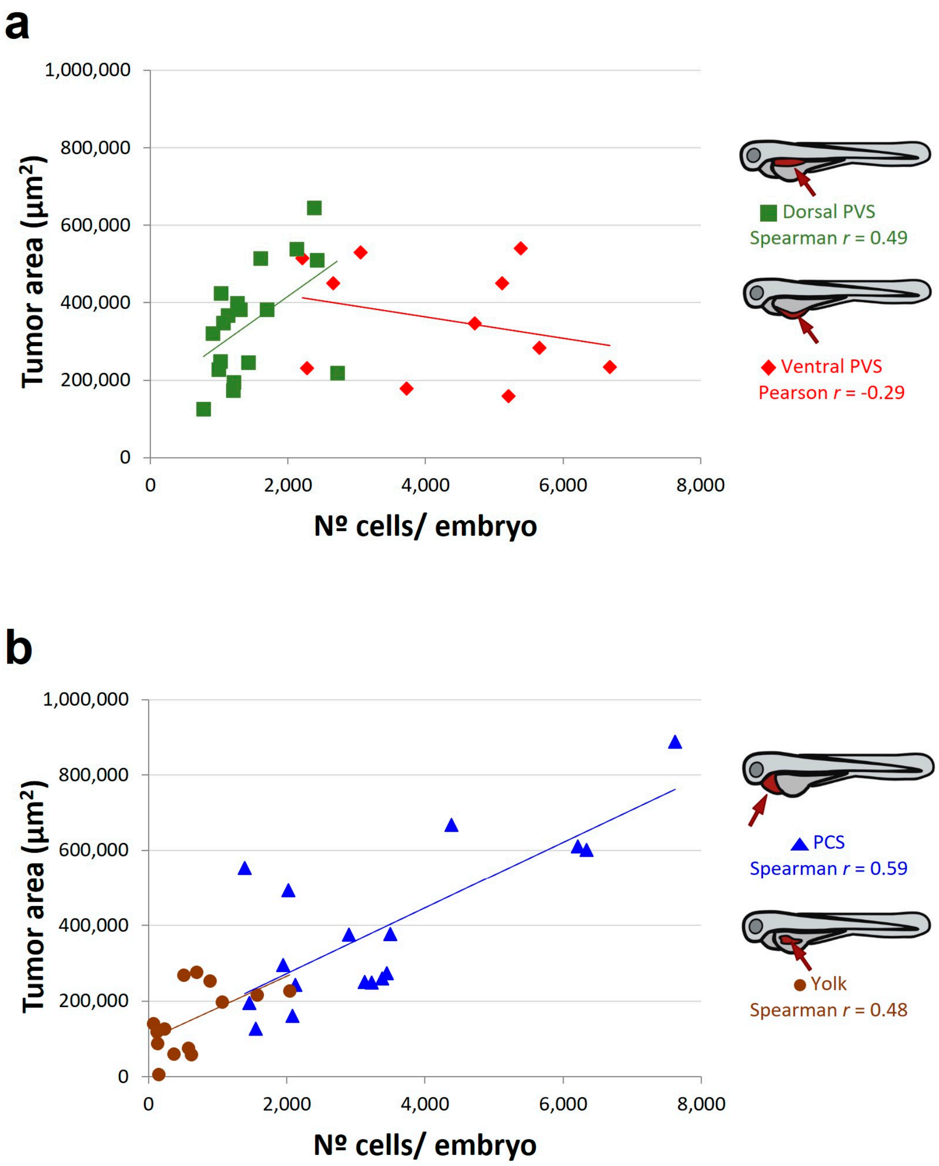

Figure S5. Correlation between the tumor area measured by imaging and the nº of cells/embryo quantified by qPCR (after an inverse interpolation using standard curves) according to each site of injection. **(a)** Correlation between the tumor area and the nº of cells determined using cDNA as the PCR template and hprt1 primers for 888mel mcherry cells implanted into the ventral or dorsal PVS. (**b**) As for (a) but cells implanted into the PCS or yolk sac.

	Site of injection	Monitoring technique	
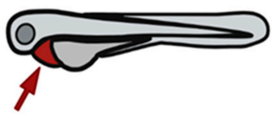	PCS	Imaging	qPCR
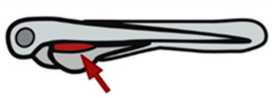	Dorsal PVS	Imaging	qPCR
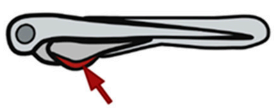	Ventral PVS	Imaging	qPCR
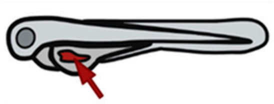	Yolk	Imaging	qPCR

Figure S6. Summary of the best technique to employ in xenograft monitoring depending on the site of cancer cell implantation. Green = reliable; red = unreliable.

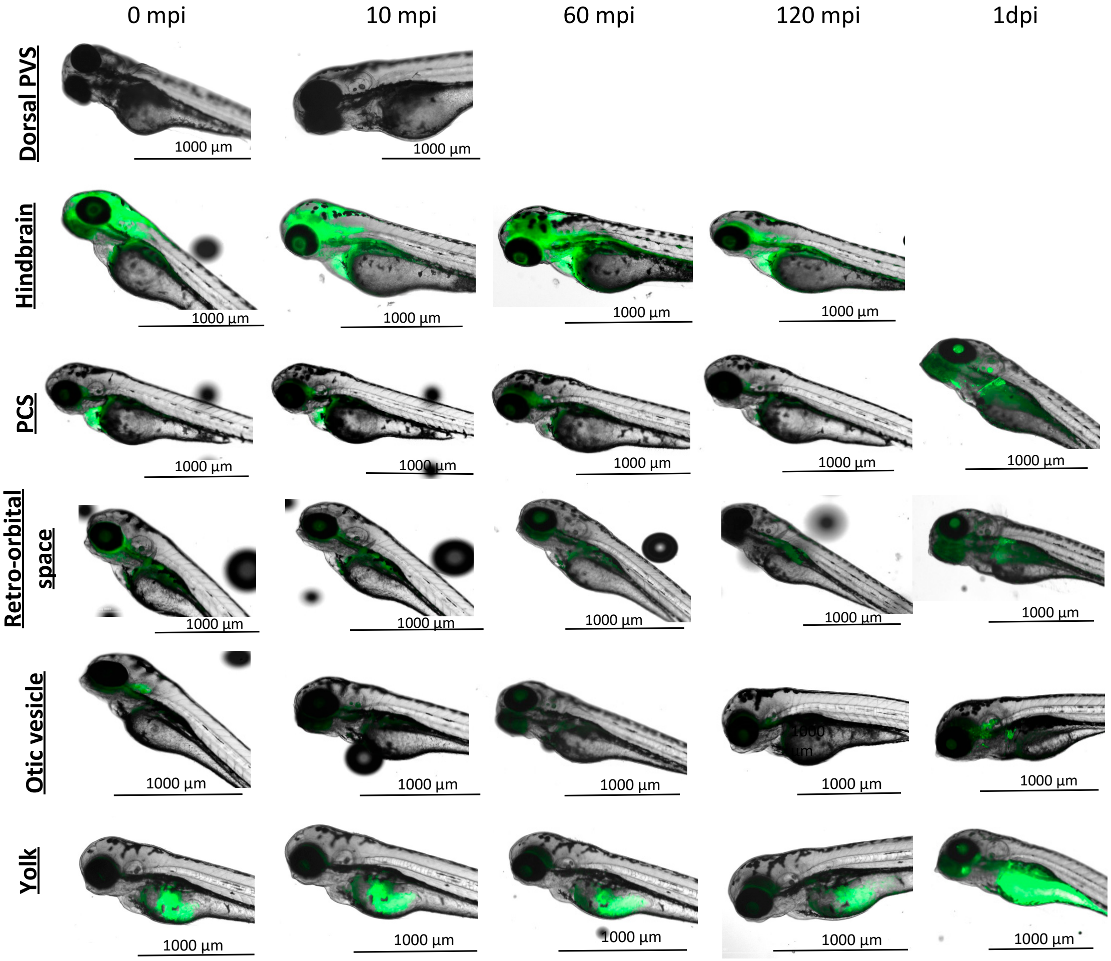

Figure S7. Representative images of 3-day old zebrafish embryos injected with Alexa Fluor 488 dextran into different locations at 0 mpi (minutes post injection), 10 mpi, 60 mpi, 120 mpi and 1 dpi.

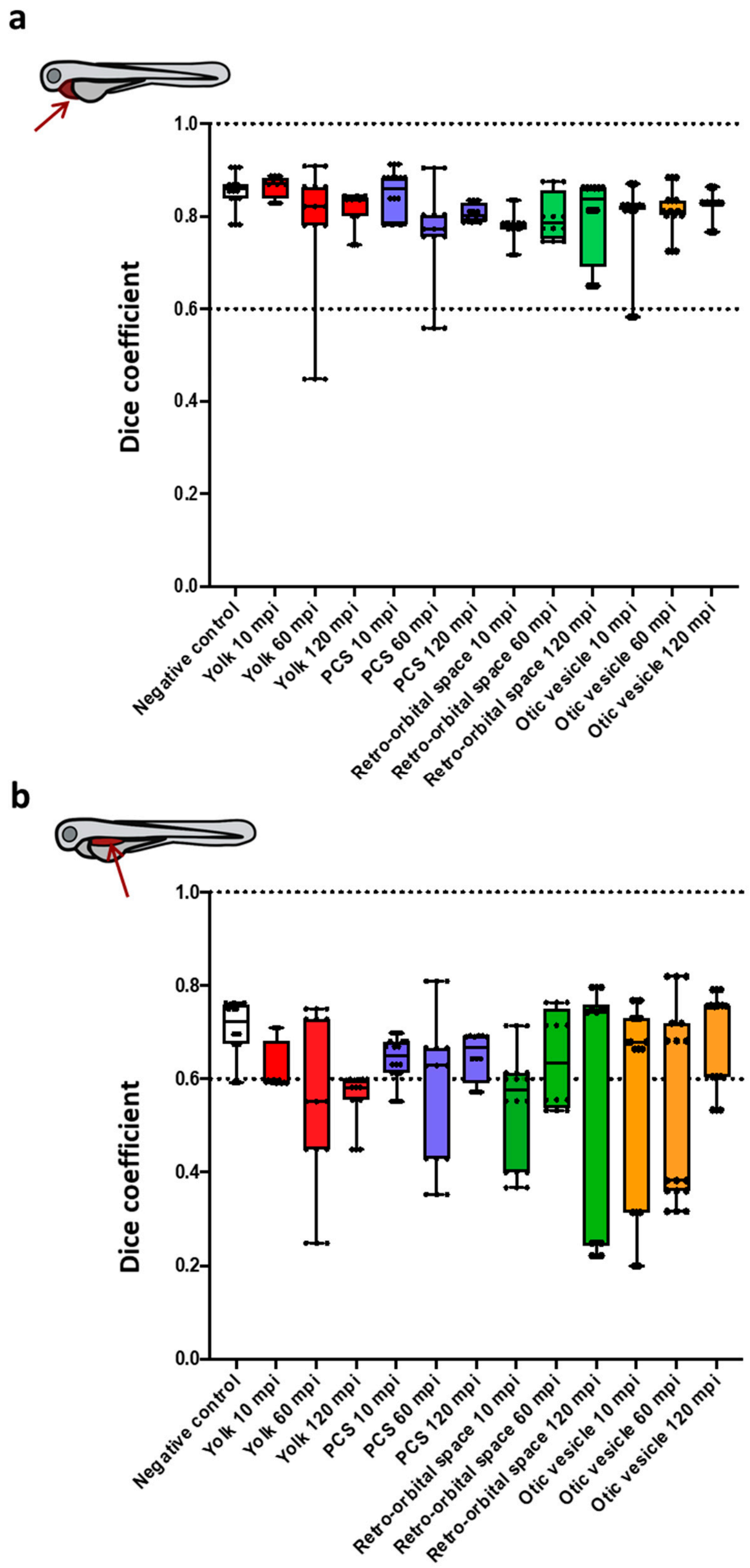

Figure S8. Dice coefficient calculated from embryos inoculated with Alexa Fluor 488 into different locations and manually segmented in Dorsal PVS (a) and PCS (b). Segmentations were considered successful if Dice coefficient was higher than 0.60 (Supplementary Materials and Methods).

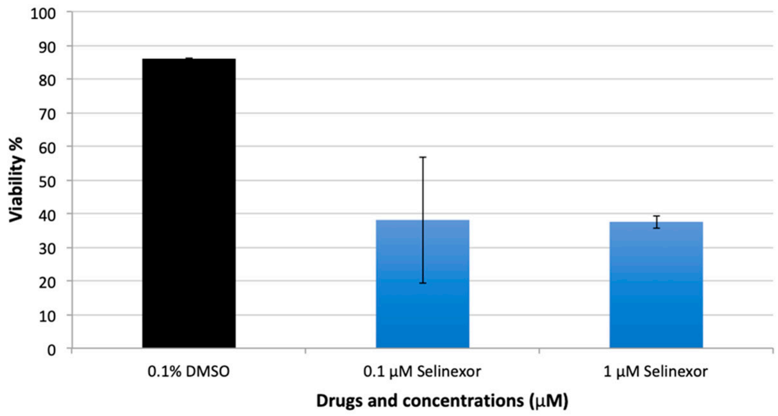

Figure S9. Viability of 888mel mCherry cells cultured with vehicle (0.1% DMSO) or 2 concentrations of anti-cancer drug Selinexor determined by MTS assay. Graph represents mean and SD error bars.

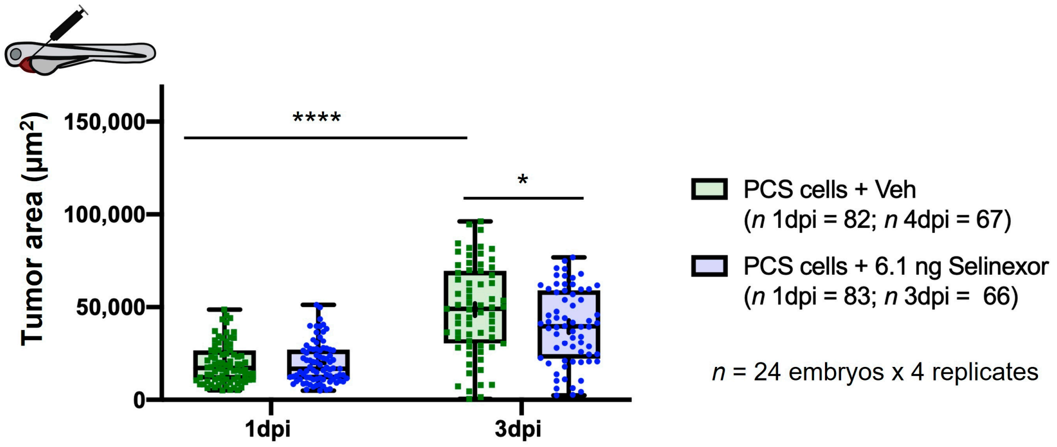

Figure S10. Efficacy of 6.1 ng Selinexor in zebrafish embryos xenografted with 888mel mCherry cells into PCS and administrated by intra-tumoral injection during 2 consecutive days until 3dpi. Vehicle = 56.25% DMSO + 10% Tween 20 + saline solution. Data are presented as box-leaf plots, where the box indicates IQR, line the median value, + the mean value, and leafs the 5-95 percentile range. Each dot signifies an engrafted embryo and only embryos where the tumor area exceeded a pre-defined threshold were included for analysis. Vehicle-treated embryos by intratumoral injection showed a RTG of 181.62% (SD=167.19%), showing tumor engraftment. Statistical significance was evaluated using an unpaired t –test or Wilcoxon matched-pairs signed rank test (* = p-value < 0.05; **** = p-value < 0.0001).

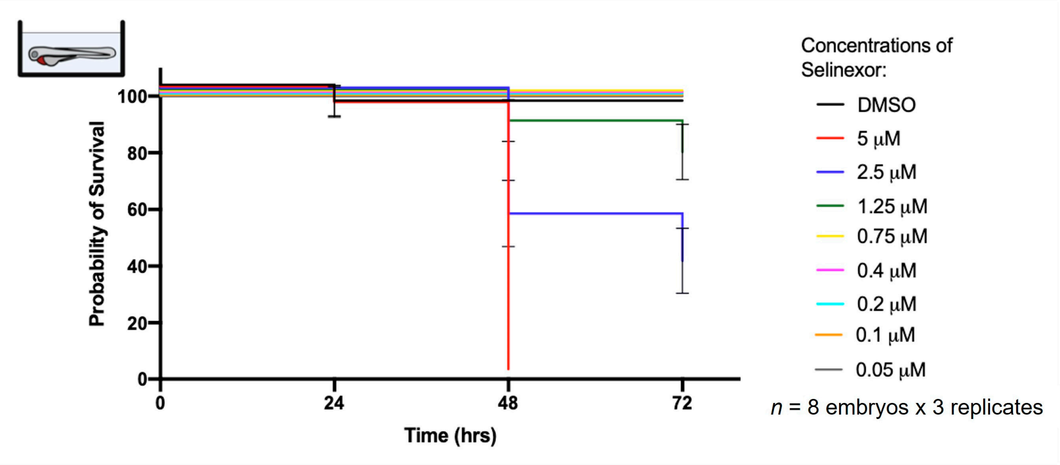

Figure S11. Maximum tolerated dose assays of Selinexor dissolved into the zebrafish medium. Survival data plotted as Kaplan-Meier plots.

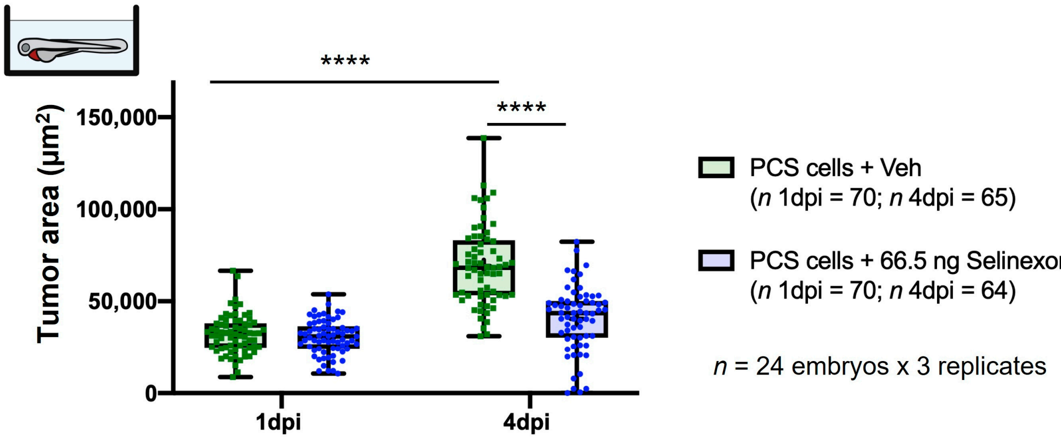

Figure S12. Efficacy of 66.5 ng Selinexor in zebrafish embryos engrafted with ~ 1,000 888mel mCherry cells into the PCS and treated over 3 consecutive days until 4 dpi by immersion (Vehicle = 0.01% DMSO + E3 medium). Data are presented as box-leaf plots, where the box indicates IQR, line the median value, + the mean value, and leafs the 5-95 percentile range. Each dot signifies an engrafted embryo and only embryos where the tumor area exceeded a pre-defined threshold were included for analysis. Vehicle-treated embryos by microinjection showed a RTG of 129.11% (SD= 67.44%) showing tumor engraftment. At 4 dpi, TA in xenografted embryos treated with Selinexor decreased significantly comparing to the embryos treated with the vehicle (p-value<0,0001). The difference in tumor growth between experimental groups was 96.51%. Statistical significance evaluated using paired and un-paired t test (**** = p-value < 0.0001).

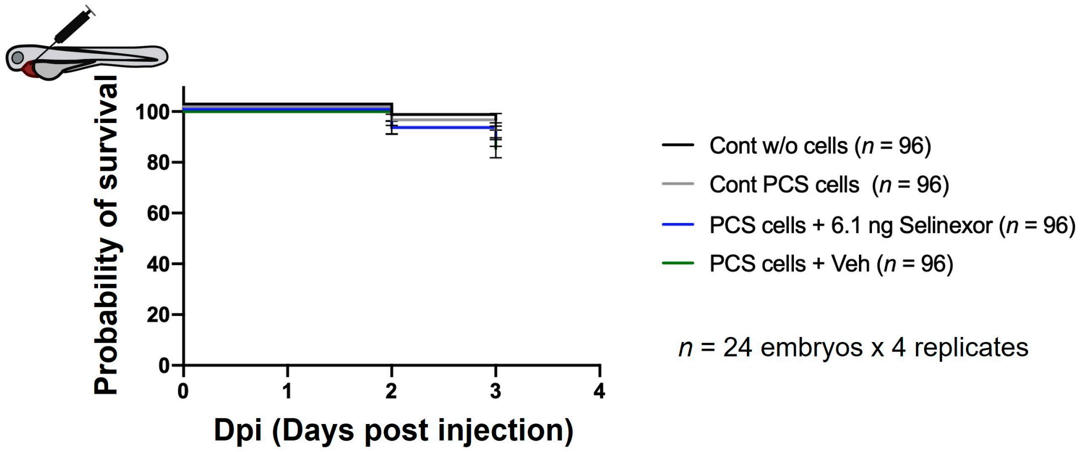

Figure S13. Impact of 6.1 ng Selinexor administered by different routes on the survival of engrafted embryos for 2 consecutive days up to 3 dpi. Vehicle = 56.25% DMSO + 10% Tween 20 + saline solution. Data presented as Kaplan-Meier survival curves.

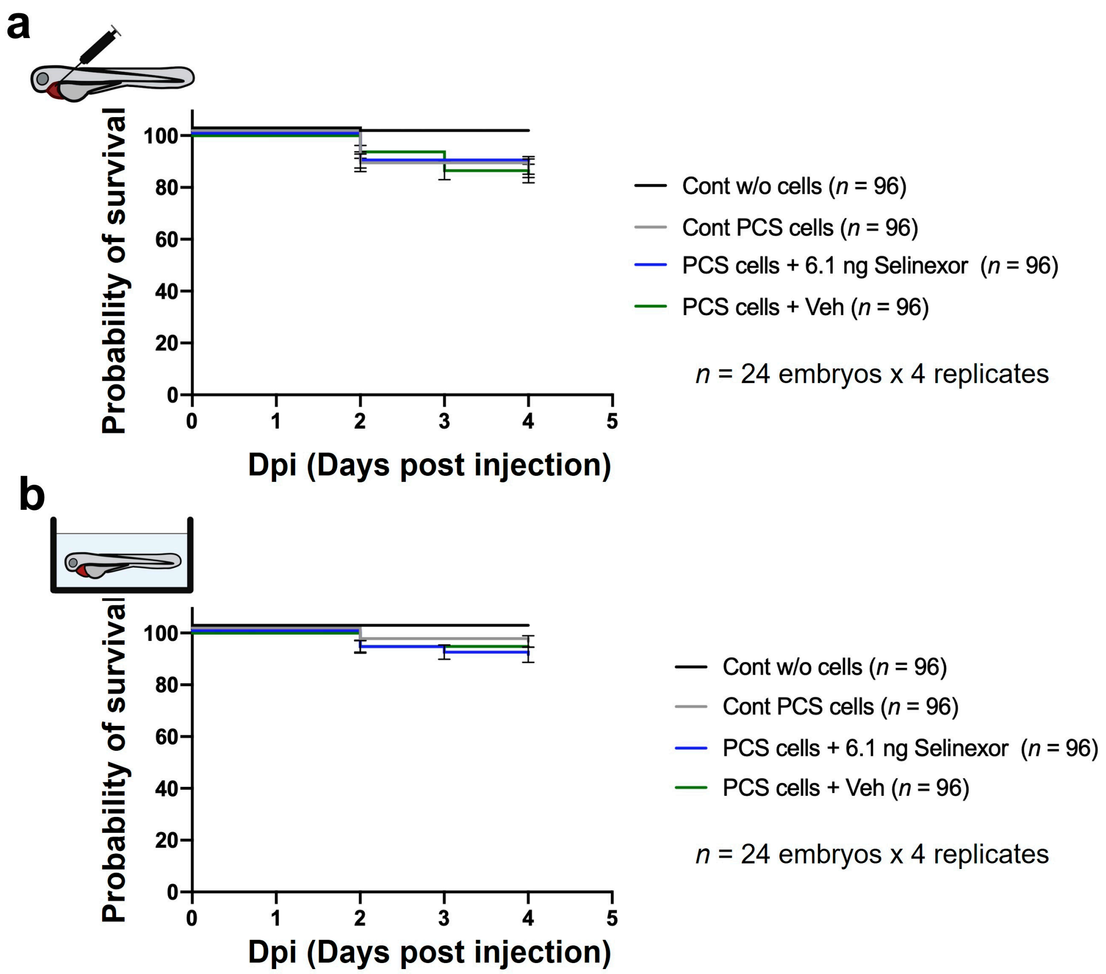

Figure S14. Impact of 6.1 ng Selinexor treatment by different administration routes on the survival of engrafted embryos over 3 consecutive days up to 4 dpi. **(a)** Survival after Selinexor or vehicle (56.25% DMSO + 10% Tween 20 + saline solution) administration by intratumoral injection. **(b)** Survival after Selinexor or vehicle (0.01% DMSO + E3 medium) administration by immersion. Data presented as Kaplan-Meier survival curves.

Table S1. Review of the 388 publications published until 2021 regarding the zebrafish xenotransplantation model.

Tumor type	Site of cell implantation	References
Breast cancer	PVS	(1)
Ovarian cancer	DoC	(2)
Brain cancer	Brain	(3)
Pancreatic cancer	PVS	(4)
Squamous cell carcinoma	PVS	(5)
Colorectal cancer	Yolk	(6)
Breast cancer<br>Colorectal cancer	PVS	(7)
Neuroblastoma	Brain	(8)
Malignant melanoma	DoC	(9)
Glioblastoma	Brain	(10)
Leukemia	Yolk	(11)
Leukemia	Yolk	(12)
Retinoblastoma	Eye	(13)
Breast cancer	DoC	(14)
Heparocarcinoma	Yolk	(15)
Ovarian cancer	Yolk	(16)
Pancreatic cancer	PVS	(17)
Prostate cancer	Yolk	(18)
Melanoma	PVS	(19)
Ewing sarcoma	Yolk	(20)
Brain tumor	Brain	(21)
Prostate cancer	Sinus venosus, subcutaneus	(22)
Hepatocarcinoma	PVS	(23)
Colorectal cancer	PVS	(24)
Leukemia	Yolk	(25)
Breast cancer	Yolk	(26)
Colon cancer	PVS	(27)
Prostate cancer	Yolk	(28)
Melanoma<br>Breast cancer<br>Heparocarcinoma	PVS	(29)
Melanoma	PVS	(30)
Prostate cancer	Yolk	(31)
Colorectal cancer	Yolk	(32)
Breast cancer	Yolk	(33)
Gioblastoma	Brain	(34)
Glioblastoma	Brain	(35)
Conjuntival melanoma	DoC	(36)
Melanoma	Yolk	(37)
Thyroid carcinoma	Yolk	(38)

Oral squomaous	PCS	(39)
Melanoma	Yolk	(40)
Colorectal	Yolk	(41)
Melanoma	PCS	(42)
Retinoblastoma	Retina	(43)
Adenoid cystic carcinoma	PVS	(44)
Lung cancer	Yolk	(45)
Gastric cancer	PVS	(46)
Lung cancer	Yolk	(47)
Prostate cancer	DoC	(48)
Fibrosarcoma	Yolk	(49)
Lung cancer	Yolk	(50)
Oral cancer	Yolk	(51)
Breast cancer	Yolk	(52)
Leukemia	Yolk	(53)
Ovarian	Yolk	(54)
Colon cancer	PVS	(55)
Breast cancer	Yolk	(56)
Melanoma	Blastula	(57)
Liver	Yolk	(58)
Conjuntival melanoma	DoC<br>Eye	(59)
Colorectal cancer	Yolk	(60)
Hepatocarcinoma	Yolk	(61)
Pancratic cancer	Yolk	(62)
Melanoma	Yolk	(63)
Colorectal cancer	PCS	(64)
Breast cancer	PVS	(65)
Thyroid carcinoma	PVS	(66)
Pancreatic cancer	Yolk	(67)
Breast cancer	DoC	(68)
Cervical caner	Cardinal vein	(69)
Pancreatic cancer	Yolk	(70)
Brain tumor	Brain	(71)
Breast cancer	Yolk	(72)
Glioblastoma	Yolk	(73)
Breast cancer	PVS	(74)
Colorectal cancer	PVS	(75)
Neuroblastoma<br>Prostate cancer	Yolk	(76)
Uveal melanoma	Yolk	(77)
Melanoma	Yolk	(78)
Melanoma	Yolk	(79)
Melanoma	Yolk	(80)
Leukemia	PCS	(81)

Glioblastoma	Brain	(82)
Glioblastoma	PVS	(83)
Gastric cancer	PVS	(84)
Neuroendocrine tumors	PVS	(85)
Neuroendocrine tumors	PVS	(86)
Leukemia	Yolk<br>PCS	(87)
Glioma	Yolk	(88)
Breast cancer<br>Prostate cancer	Yolk	(89)
Prostate cancer	Yolk	(90)
Adenocortical carcinoma	Yolk	(91)
Cervical cancer	Yolk	(92)
Colorectal cancer	PVS	(93)
Breat cancer	PVS	(94)
Ovarian cancer	Yolk	(95)
Breast cancer	Yolk	(96)
Pancreatic cancer	Yolk	(97)
Pancreatic cancer	PVS	(98)
Breast cancer	Yolk	(99)
Colorectal cancer<br>Melanoma<br>Pancreatic cancer	Yolk	(100)
Glioblastoma multiforme	Optic tectum	(101)
Glioblastoma	Brain	(102)
Leukemia	Yolk<br>PCS	(103)
Breast cancer	Yolk	(104)
Breast cancer	Yolk	(105)
Breast cancer<br>Prostate cancer	DoC	(106)
Melanoma	PVS	(107)
Colorectal	Yolk	(108)
Prostate cancer<br>Melanoma<br>Lung cancer	DoC	(109)
Colorectal cancer	PVS	(110)
Gastric cancer	Yolk	(111)
Breast cancer	Yolk	(112)
Head and neck squamous cell carcinoma	PVS	(113)
Breast cancer	PVS	(114)
Glioblastoma	Yolk	(115)
Lung cancer	Yolk	(116)
Breast cancer	Yolk	(117)
Melanoma	Circulation	(118)
Hepatocarcinoma	Yolk	(119)

Osteosarcoma	Yolk	(120)
Breast cancer	Yolk	(121)
Breast cancer<br>Colorectal cancer<br>Glioblastoma	PVS	(122)
Leukemia	Yok	(123)
Lung cancer	Yolk	(124)
Osteosarcoma	Yolk	(125)
Osteosarcoma	Yolk	(126)
Colorectal	Yolk	(127)
Retinoblastoma	Intravitreal	(128)
Squamous cancer	Yok	(129)
Colon cancer<br>Oral squamous cell carcinoma	Yolk	(130)
Colorectal cancer	Yolk	(131)
Hepatocarcinoma	Yolk	(132)
Hepatocarcinoma	Yolk	(133)
Head and neck squamous cell carcinoma	PVS	(134)
Lung cancer<br>Hepatoma	Yolk	(135)
Malignat peripheral nerve sheat tumor	PCS	(136)
Pancreatic cancer	DoC	(137)
Breast cancer	PVS	(138)
Hepatocarcinoma		(139)
Breast cancer	DoC	(140)
Glioblastoma	Yolk	(141)
Glioblastoma multiforme	Yolk<br>Hindbrain	(142)
Glioma	Yolk<br>Hindbrain	(143)
Lung cancer	Yolk	(144)
Ovarian cancer	Yolk	(145)
Melanoma	Yolk	(146)
Breast cancer<br>Fibrosarcoma<br>Ovarian cancer	PVS	(147)
Schaucowma	Yolk	(148)
Lung cancer	Yolk	(149)
Ovarian cancer	Yolk	(150)
Leukemia	Yolk	(151)
Lung cancer	Yolk	(152)
Lung cancer	Yolk	(153)
Breast cancer	PVS	(154)
Breast cancer	Yolk	(155)
Breast cancer	Yolk	(156)

Breast cancer	Yolk	(157)
Leukemia	Yolk	(158)
Lung cancer	Yolk	(159)
Heparocarcinoma	PVS	(160)
Lung cancer	Brain	(161)
Bone marrow	Yolk	(162)
Melanoma	DoC	(163)
Mieloma	PVS	(164)
Prostate cancer	Yolk	(165)
Cholangiocarcinoma	Yolk	(166)
Heparocarcinoma	Yolk	(167)
Breast cancer	PVS	(168)
Lung cancer	Yolk	(169)
Liver	PVS	(170)
Breast cancer	DoC	(171)
Squomaus oral carcinoma	PVS	(172)
Lung cancer	Yolk	(173)
Leukemia	Yolk	(174)
Pancreatic cancer	Yolk	(175)
Melanoma	Yolk	(176)
Lymphoma	Yolk	(177)
Breast cancer	DoC	(178)
Breast cancer	Yolk	(179)
Lung cancer		(180)
Bone Marrow cancer	Yolk	(181)
Lung cancer	PVS	(182)
Mouse cells	PVS	(183)
Prostate cancer	Yolk	(184)
Breast cancer	DoC	(185)
Squamous cell carcinoma	Yolk	(186)
Colorectal cancer	Yolk	(187)
Breast cancer	Yolk	(188)
Prostate cancer	Yolk	(189)
Breast cancer	PVS	(190)
Breast cancer	DoC	(191)
Hepatocarcinoma	DoC	(192)
Breast cancer<br>Cervical carcinoma<br>Oral squamous cell carcinoma	PVS	(193)
Glioblastoma	Yolk	(194)
Breast cancer<br>Ovarian cancer	PVS	(195)
Prostatic cancer	Yolk	(196)
Oral squamous cell carcinoma	PVS	(197)

Colorectal	PVS	(198)
Stem cells	DoC	(199)
Breast cancer	Yolk	(200)
Lung cancer	Yolk	(201)
Breast cancer	PVS	(202)
Melanoma	PVS	(203)
Pancratic cancer	Yolk	(204)
Breast cancer	Yolk	(205)
Leukemia	Circulation	(206)
Ewing sarcoma	PVS	(207)
Breast cancer	Circulation	(208)
Lung cancer	Yolk	(209)
Cervical carcinoma	PVS	(210)
Melanoma	PCS	(211)
Cervical carcinoma	Yolk	(212)
Muscle cells	Intramuscular	(213)
Breat cancer	Yolk	(214)
Colorectal cancer<br>Melanoma	Yolk	(215)
Breast cancer	Yolk	(216)
Hepatocarcinoma	PVS	(217)
Colorectal	PVS	(218)
Leukemia	Cardial vein	(219)
Glioblastoma	Other	(220)
Cervical carcinoma	PVS	(221)
Breast cancer	DoC	(222)
Leukemia	Yolk	(223)
Glioblastoma multiforme	Brain	(224)
Breast cancer	DoC<br>PVS	(225)
Breast cancer	DoC	(226)
Squomaus oral carcinoma	Yolk	(227)
Melanoma	Yolk	(228)
Colorectal cancer	Yolk	(229)
Mieloma	DoC	(230)
Fibrosarcoma	PVS	(231)
Mieloma	Heart	(232)
Colorectal cancer	PVS	(233)
Colorectal cancer	PVS	(234)
Lung cancer	PVS	(235)
Breast cancer	Yolk	(236)
Lung cancer	Yolk	(237)
Brain tumor	Yolk	(238)
Melanoma	PVS	(239)
Colorectal cancer	DoC	(240)

Breast cancer	PCS	(241)
Breast cancer	DoC	(242)
Neuroblastoma	Yolk	(243)
Breast cancer	Yolk	(244)
Gastric cancer	Yolk	(245)
Lung cancer	PVS	(246)
Hepatcarcinoma	Yolk	(247)
Breast cancer	Yolk	(248)
Lung cancer	PVS	(249)
Uveal melanoma	Yolk<br>Eye	(250)
Melanoma	PCS	(251)
Melanoma	PVS	(252)
Leukemia	DoC	(253)
Breast cancer<br>Fibrosarcoma<br>Melanoma	Peritoneal	(254)
Fibrosarcoma<br>Breast cancer<br>Colorectal cancer	Cardinal vein	(255)
Prostate cancer	Yolk	(256)
Lung cancer	Yolk	(257)
Breast cancer	Yolk	(258)
Lymphoma	Yolk	(259)
Hepatocarcinoma	Yolk	(260)
Breast cancer<br>Colon cancer<br>Pancreatic cancer<br>Prostate cancer	PVS	(261)
Heparocarcinoma	PVS	(262)
Gliblastoma	Brain	(263)
Mamary tumor	Yolk<br>Doc	(264)
Breast cancer	Yolk	(265)
Hepatocellular carcinoma	Yolk	(266)
Melanoma	Yolk	(267)
Colorectal cancer	PVS	(268)
Heparocarcinoma	Yolk	(269)
Gastric cancer	Yolk	(270)
Gastric cancer	PVS	(271)
Colorectal cancer	Yolk	(272)
Breast cancer	DoC	(273)
Breast cancer<br>Ewing Sarcoma<br>Pancreatic cancer	Circulation	(274)
Pancreatic cancer<br>Stomach cancer<br>Colon cancer	Yolk	(275)

Melanoma	Yolk	(276)
Ewing sarcoma	Eye<br>Yolk	(277)
Uveal melanoma	Yolk	(278)
Breast cancer	PVS	(279)
Colorectal cancer	PVS	(280)
Glioblastoma	DoC	(281)
Breast cancer	PVS	(282)
Breast cancer	PVS	(283)
Breast cancer	Yolk	(284)
Melanoma	Yolk	(285)
Leukemia	Yolk	(286)
Glioblastoma multiforme	Brain	(287)
Glioblastoma	Yolk<br>Brain	(288)
Pancreatic cancer	Yolk	(289)
Squamous cancer	DoC	(290)
Prostate cancer	Yolk	(291)
Ovarian cancer	Yolk	(292)
Leukemia	Yolk	(293)
Myeloma	PVS	(294)
Breast cancer	Yolk	(295)
Breast cancer	PVS	(296)
Pancreatic cancer	Yolk	(297)
Ewing sarcoma	Yolk	(298)
Breast cancer	Vein	(299)
Lung cancer	PVS	(300)
Breast cancer	PVS	(301)
Squomaus oral carcinoma	PVS	(302)
Lung cancer	Yolk	(303)
Heparocarcinoma	Yolk	(304)
Lung cancer	PVS	(305)
Melanoma	PVS	(306)
Glioblastoma	Brain	(307)
Breast cancer	PVS	(308)
Hepatocarcinoma	Yolk	(309)
Sarcoma	Yolk	(310)
Pancreatic cancer	Yolk	(311)
Glioblastoma	Brain	(312)
Glioblastoma	Brain	(313)
Oral squamous cell carcinoma	Yolk	(314)
Brain tumor	Brain	(315)
Pancreatic cancer	Otolito	(316)
Lung cancer	Yolk	(317)
Neuroblastoma	PVS	(318)

Melanoma	Circulation<br>PVS	(319)
Gastric cancer	Yolk	(320)
Breast cancer	PVS	(321)
Breast cancer	Yolk	(322)
Hepatocarcinoma	Yolk	(323)
Nasopharyngeal carcinoma	Yolk	(324)
Gastric cancer	Yolk	(325)
Breast cancer	PVS	(326)
Breast cancer	PVS	(327)
Prostate cancer	Sinus venosus	(328)
Gastric cancer	PVS	(329)
Hepatocarcinoma	Yolk	(330)
Colorectal cancer	Yolk	(331)
Lung cancer	PVS	(332)
Rhandomiosarcoma	Yolk	(333)
Glioma	Yolk	(334)
Glioma	Yolk	(335)
Glioma	Yolk	(336)
Cervical carcinoma	DoC	(337)
Heparocarcinoma	Circulation	(338)
Breast cancer	Circulation	(339)
Breast cancer	Yolk	(340)
Breast cancer	Yolk	(341)
Breast cancer	Yolk	(342)
Leukemia	Yolk	(343)
Sarcoma	Yolk	(344)
Pancreatic cancer	Yolk	(345)
Oral cancer	Yolk	(346)
Glioblastoma	Yolk	(347)
Glioma	Otic tectum	(348)
Melanoma	PVS	(349)
Breast cancer	Yolk	(350)
Glioblastoma	Brain	(351)
Nasopharyngeal carcinoma	Yolk	(352)
Melanoma	PVS	(353)
Prostate cancer	Yolk	(354)
Leukemia	Yolk	(355)
Leukemia	Yolk	(356)
Gastric cancer	PVS	(357)
Breast cancer	Yolk	(358)
Melanoma	PVS	(359)
Gastric cancer	Yolk	(360)
Lung cancer	Yolk	(361)

Gastric cancer	PVS	(362)
Leukemia	Yolk	(363)
Glioma	Yolk	(364)
Colon<br>Melanoma	PVS	(365)
Breast cancer	PVS	(366)
Melanoma	PVS	(367)
Breast cancer	PVS	(368)
Hepatocarcinoma	Yolk	(369)
Melanoma	PVS	(370)
Pancreatic cancer	Yolk	(371)
Leukemia	Yolk	(372)
Melanoma	PVS	(373)
Breast cancer<br>Lung cancer<br>Pancratic cancer<br>Stomach cancer	Yolk	(374)

Table S2. Total number of xenografted embryos before and after the application of the defined inclusion criteria.

Site of injection	Total number of xenografted embryos	Final number of embryos considered for the analysis
PCS	72	69
Dorsal PVS	72	69
Ventral PVS	72	68
Yolk	71	71

Table S3. Evaluation of the minimum number of replicates required. Data from the 6.1 ng selinexor administered by intratumoral injection until 3 dpi efficacy experiment was employed. R = replicate, veh = vehicle (* = p-value < 0.05; ** = p-value < 0.01; *** = p-value < 0.001; **** = p-value < 0.0001).

		selinexor 1dpi vs selinexor 3dpi	veh 1dpi vs veh 3dpi	selinexor 1dpi vs veh 1dpi	selinexor 3dpi vs veh 3dpi
4 replicates	Statistical analysis	Wilcoxon rank test	Wilcoxon rank test	Mann-Whitney test	Unpaired t test
	p-value	0.00000	0.00000	0.4184	0.0001
		****	****	ns	***
R1 R2	Statistical analysis	Wilcoxon rank test	Wilcoxon rank test	Unpaired t test	Mann-Whitney test
	p-value	0.00000	0.00000	0.7806	0.0256
		****	****	ns	*
R1 R3	Statistical analysis	Paired t test	Paired t test	Unpaired t test	Unpaired t test
	p-value	0.00000	0.00000	0.7188	0.0623
		****	****	ns	ns
R1 R4	Statistical analysis	Wilcoxon rank test	Paired t test	Mann-Whitney test	Unpaired t test
	p-value	0.00000	0.00000	0.597	0.003
		****	****	ns	**
R2 R3	Statistical analysis	Wilcoxon rank test	Paired t test	Mann-Whitney test	Unpaired t test
	p-value	0.00000	0.00000	0.5485	0.5517
		****	****	ns	ns
R2-R4	Statistical analysis	Wilcoxon rank test	Wilcoxon rank test	Mann-Whitney test	Mann Witney
	p-value	0.00000	0.00000	0.6986	0.1592
		****	****	ns	ns
R3-R4	Statistical analysis	Wilcoxon rank test	Wilcoxon rank test	Mann-Whitney test	Unpaired t test
	p-value	0.00000	0.00000	0.472	0.1142
		****	****	ns	ns
R1-R2-R3	Statistical analysis	Wilcoxon rank test	Wilcoxon rank test	Mann-Whitney test	Mann Witney
	p-value	0.00000	0.00000	0.6944	0.0915
		****	****	ns	ns
R1-R2-R4	Statistical analysis	Wilcoxon rank test	Wilcoxon rank test	Mann-Whitney test	Unpaired t test
	p-value	0.00000	0.00000	0.6847	0.0108
		****	****	ns	*
R1-R3-R4	Statistical analysis	Wilcoxon rank test	Wilcoxon rank test	Mann-Whitney test	Unpaired t test
	p-value	0.00000	0.00000	0.7918	0.0131
		****	****	ns	*
R2-R3-R4	Statistical analysis	Wilcoxon rank test	Wilcoxon rank test	Mann-Whitney test	Unpaired t test
	p-value	0.00000	0.00000	0.7985	0.0917
		****	****	ns	ns

Table S4. Evaluation of the minimum number of replicates required. Data from the 6.1 ng selinexor administered by inratumoral injection until 4 dpi efficacy experiment was employed. R = replicate, veh = vehicle (* = p-value < 0.05; ** = p-value < 0.01; *** = p-value < 0.001; **** = p-value < 0.0001).

		selinexor 1dpi vs selinexor 4dpi	veh 1dpi vs veh 4dpi	selinexor 1dpi vs veh 1dpi	selinexor 4dpi vs veh 4dpi
4 repeticiones	Statistical analysis	Wilcoxon rank test	Wilcoxon rank test	Mann-Whitney test	Unpaired t test
	p-value	0.0000	0.00000	0.4184	0.0000
		****	****	ns	****
R1 R2	Statistical analysis	Wilcoxon rank test	Wilcoxon rank test	Mann-Whitney test	Unpaired t test
	p-value	0.0000	0.0000	0.2115	0.1078
		****	****	ns	ns
R1 R3	Statistical analysis	Paired t test	Wilcoxon rank test	Mann-Whitney test	Unpaired t test
	p-value	0.0000	0.0000	0.1445	0.0981
		****	****	ns	ns
R1 R4	Statistical analysis	Paired t test	Wilcoxon rank test	Mann-Whitney test	Unpaired t test
	p-value	0.0009	0.0000	0.5098	0.0369
		***	****	ns	*
R2 R3	Statistical analysis	Wilcoxon rank test	Wilcoxon rank test	Mann-Whitney test	Unpaired t test
	p-value	0.0000	0.0000	0.4197	0.0013
		****	****	ns	*
R2-R4	Statistical analysis	Paired t test	Wilcoxon rank test	Mann-Whitney test	Unpaired t test
	p-value	0.0008	0.0000	0.9716	0.0003
		***	****	ns	**
R3-R4	Statistical analysis	Paired t test	Paired t test	Unpaired t test	Unpaired t test
	p-value	0.0005	0.0000	0.716	0.0001
		***	****	ns	***
R1-R2-R3	Statistical analysis	Wilcoxon rank test	Wilcoxon rank test	Mann-Whitney test	Unpaired t test
	p-value	0.0000	0.0000	0.154	0.0075
		****	****	ns	**
R1-R2-R4	Statistical analysis	Wilcoxon rank test	Wilcoxon rank test	Mann-Whitney test	Unpaired t test
	p-value	0.0000	0.0000	0.4408	0.0024
		****	****	ns	**
R1-R3-R4	Statistical analysis	Paired t test	Wilcoxon rank test	Mann-Whitney test	Unpaired t test
	p-value	0.0000	0.0000	0.5426	0.002
		****	****	ns	**
R2-R3-R4	Statistical analysis	Wilcoxon rank test	Wilcoxon rank test	Mann-Whitney test	Unpaired t test
	p-value	0.0000	0.0000	0.9235	0.0000
		****	****	ns	****

Table S5. Statistical analyses of Kaplan-Meier curves of embryos engrafted with approximately 1,000 888mel mCherry cells into different injection sites performed by a log rank test (* = p-value < 0.05; ** = p-value < 0.01; *** = p-value < 0.001; **** = p-value < 0.0001).

	PCS	PCS cont	Dorsal-PVS	Dorsal-PVS cont	Ventral-PVS	Ventral-PVS cont	Yolk
PCS							
PCS cont	ns						
Dorsal-PVS	*	**					
Dorsal-PVS cont	**	****	ns				
Ventral-PVS	****	****	**	ns			
Ventral-PVS cont	*	**	ns	ns	**		
Yolk	****	****	****	**	ns	****	
Yolk cont	****	****	**	*	ns	**	ns

Tables S6. Statistical analyses of percentage embryo engraftment of cancer cells from each implantation site performed by one-way ANOVA (* = p-value < 0.05; ** = p-value < 0.01; *** = p-value < 0.001; **** = p-value < 0.0001).

2dpi	PCS	Dorsal-PVS	Ventral-PVS	Yolk
PCS				
Dorsal-PVS	ns			
Ventral - PVS	ns	ns		
Yolk	**	**	**	

a

3dpi	PCS	Dorsal-PVS	Ventral-PVS	Yolk
PCS				
Dorsal-PVS	ns			
Ventral - PVS	ns	ns		
Yolk	**	**	**	

b

References

1. Abrahamsson A, Rodriguez GV, Dabrosin C. Fulvestrant-mediated attenuation of the innate immune response decreases ERþ breast cancer growth in vivo more effectively than tamoxifen. *Cancer Res* **2021**;80:4487–99.
2. Abreu M, Cabezas-Sainz P, Alonso-Alconada L, Ferreirós A, Mondelo-Macía P, Lago-Lestón RM, *et al*. Circulating Tumor Cells Characterization Revealed TIMP1 as a Potential Therapeutic Target in Ovarian Cancer. *Cells* **2020**;9:1–19.
3. Al-Akhrass H, Conway JRW, Poulsen ASA, Paatero I, Kaivola J, Padzik A, *et al.* A feed-forward loop between SorLA and HER3 determines heregulin response and neratinib resistance. *Oncogene* **2021**;40:1300–17.
4. Al-Ismaeel Q, Neal CP, Al-Mahmoodi H, Almutairi Z, Al-Shamarti I, Straatman K, *et al*. ZEB1 and IL-6/11-STAT3 signalling cooperate to define invasive potential of pancreatic cancer cells via differential regulation of the expression of S100 proteins. *Br J Cancer* **2019**;121:65–75.
5. Al-Samadi A, Tuomainen K, Kivimäki A, Salem A, Al-Kubati S, Hyytiäinen A, *et al.* PCR-based zebrafish model for personalised medicine in head and neck cancer. *J Transl Med* **2019**;17:235.
6. Alburquerque-gonzález B, Bernabé-garcía Á, Bernabé-garcía M, Ruiz-sanz J, López-calderón FF, Gonnelli L, *et al.* The fda-approved antiviral raltegravir inhibits fascin1-dependent invasion of colorectal tumor cells in vitro and in vivo. *Cancers (Basel)* **2021**;13:1–22.
7. Rebelo de Almeida C, Mendes RV, Pezzarossa A, Gago J, Carvalho C, Alves A, *et al*. Zebrafish xenografts as a fast screening platform for bevacizumab cancer therapy. *Commun Biol* **2020**;3:1–13.
8. Almstedt E, Elgendy R, Hekmati N, Rosén E, Wärn C, Olsen TK, *et al.* Integrative discovery of treatments for high-risk neuroblastoma. *Nat Commun* **2020**;11:1–15.
9. Andreiuk B, Reisch A, Lindecker M, Follain G, Peyriéras N, Goetz JG, *et al.* Fluorescent Polymer Nanoparticles for Cell Barcoding In Vitro and In Vivo. *Small* **2017**;13:1–13.
10. Angom RS, Mondal SK, Wang F, Madamsetty VS, Wang E, Dutta SK, *et al*. Ablation of neuropilin-1 improves the therapeutic response in conventional drug-resistant glioblastoma multiforme. *Oncogene* **2020**;39:7114–26.
11. Arriazu E, Vicente C, Pippa R, Peris I, Martínez-Balsalobre E, García-Ramírez P, *et al*. A new regulatory mechanism of protein phosphatase 2A activity via SET in acute myeloid leukemia. *Blood Cancer J* **2020**;10:3.
12. Arumuggam N, Melong N, Too CKL, Berman JN, Rupasinghe HPV. Phloridzin docosahexaenoate, a novel flavonoid derivative, suppresses growth and induces apoptosis in T-cell acute lymphoblastic leukemia cells. *Am J Cancer Res* **2017**;7:2452–64.
13. Asnaghi L, White DT, Yoon L, Price A, Lee GY, Sahoo A, *et al.* Downregulation of Nodal inhibits metastatic progression in retinoblastoma. *Acta Neuropathol*

Commun **2019**;7:137.
14. Asokan N, Daetwyler S, Bernas SN, Schmied C, Vogler S, Lambert K, *et al.* Long-term in vivo imaging reveals tumor-specific dissemination and captures host tumor interaction in zebrafish xenografts. *Sci Rep* **2020**;10.
15. Avci ME, Keskus AG, Targen S, Isilak ME, Ozturk M, Atalay RC, *et al.* Development of a novel zebrafish xenograft model in ache mutants using liver cancer cell lines. *Sci Rep* **2018**;8:1–14.
16. Bae H, Lee JY, Yang C, Song G, Lim W. Fucoidan Derived from Fucus vesiculosus Inhibits the Development of Human Ovarian Cancer via the Disturbance of Calcium Homeostasis, Endoplasmic Reticulum Stress, and Angiogenesis. *Mar Drugs* **2020**;18:1–23.
17. Bagowski CP, You Y, Scheffler H, Vlecken DH, Schmitz DJ, Ott I. Naphthalimide gold(i) phosphine complexes as anticancer metallodrugs. *Dalt Trans* **2009**;10799.
18. Bakht MK, Lovnicki JM, Tubman J, Stringer KF, Chiaramonte J, Reynolds MR, *et al.* Differential expression of glucose transporters and hexokinases in prostate cancer with a neuroendocrine gene signature: A mechanistic perspective for 18 F-FDG imaging of PSMA-suppressed tumors. *J Nucl Med* **2020**;61:904–10.
19. Baltrunaite K, Craig MP, Palencia Desai S, Chaturvedi P, Pandey RN, Hegde RS, *et al.* ETS transcription factors Etv2 and Fli1b are required for tumor angiogenesis. *Angiogenesis* **2017**;20:307–23.
20. Ban J, Aryee DNT, Fourtouna A, van der Ent W, Kauer M, Niedan S, *et al.* Suppression of Deacetylase SIRT1 Mediates Tumor-Suppressive NOTCH Response and Offers a Novel Treatment Option in Metastatic Ewing Sarcoma. *Cancer Res* **2014**;74:6578–88.
21. Banasavadi-Siddegowda YK, Welker AM, An M, Yang X, Zhou W, Shi G, *et al.* PRMT5 as a druggable target for glioblastoma therapy. *Neuro Oncol* **2018**;20:753–63.
22. Bansal N, Davis S, Tereshchenko I, Budak-alpdogan T, Zhong H, Stein MN, *et al.* Enrichment of human prostate cancer cells with tumor initiating properties in mouse and zebrafish xenografts by differential adhesion. *Prostate* **2014**;74:187–200.
23. Bartucci M, Hussein MS, Huselid E, Flaherty K, Patrizii M, Laddha S V., *et al.* Synthesis and Characterization of Novel BMI1 Inhibitors Targeting Cellular Self-Renewal in Hepatocellular Carcinoma. *Target Oncol* **2017**;12:449–62.
24. Basti A, Fior R, Yalçin M, Póvoa V, Astaburuaga R, Li Y, et al. The core-clock gene nr1d1 impacts cell motility in vitro and invasiveness in a zebrafish xenograft colon cancer model. *Cancers (Basel)* **2020**;12:1–17.
25. Bentley VL, Veinotte CJ, Corkery DP, Pinder JB, Leblanc MA, Bedard K, et al. Focused chemical genomics using zebrafish xenotransplantation as a pre-clinical therapeutic platform for T-cell acute lymphoblastic leukemia. *Haematologica.* **2015**;100:70–6.
26. Bessou M, Lopez J, Gadet R, Deygas M, Popgeorgiev N, Poncet D, *et al.* The apoptosis inhibitor Bcl-xL controls breast cancer cell migration through mitochondria-dependent reactive oxygen species production. *Oncogene* **2020**;39:3056–74.
27. Bissey PA, Mathot P, Guix C, Jasmin M, Goddard I, Costechareyre C, *et al.* Blocking SHH/Patched interaction triggers tumor growth inhibition through

patched-induced apoptosis. *Cancer Res* **2020**;80:1970–80.
28. Bouaouiche S, Magadoux L, Dondaine L, Reveneau S, Isambert N, Bettaieb A, *et al.* Glyceryl trinitrate-induced cytotoxicity of docetaxel-resistantprostatic cancer cells is associated with differential regulation of clusterin. *Int J Oncol* **2019**;54:1446–56.
29. Britto DD, Wyroba B, Chen W, Lockwood RA, Tran KB, Shepherd PR, *et al.* Macrophages enhance Vegfa-driven angiogenesis in an embryonic zebrafish tumour xenograft model. *Dis Model Mech* **2018**;11:1–14.
30. Britto DD, Hall CJ, Astin JW. *In vivo* imaging and quantitation of the host angiogenic response in zebrafish tumor xenografts. *J Vis Exp* **2019**;150: e59849.
31. Butler MS, Roshan-Moniri M, Hsing M, Lau D, Kim A, Yen P, *et al.* Discovery and characterization of small molecules targeting the DNA-binding ETS domain of ERG in prostate cancer. *Oncotarget* **2017**;8:42438–54.
32. Cabezas-Sainz P, Guerra-Varela J, Carreira MJ, Mariscal J, Roel M, Rubiolo JA, *et al.* Improving zebrafish embryo xenotransplantation conditions by increasing incubation temperature and establishing a proliferation index with ZFtool. *BMC Cancer* **2018**;18(1):3.
33. Cabezas-Sainz P, Coppel C, Pensado-López A, Fernandez P, Muinelo-Romay L, López-López R, *et al.* Morphological abnormalities and gene expression changes caused by high incubation temperatures in zebrafish xenografts with human cancer cells. *Genes (Basel)* **2021**;12:1–12.
34. Cam M, Charan M, Welker AM, Dravid P, Studebaker AW, Leonard JR, *et al.* Δnp73/ETS2 complex drives glioblastoma pathogenesis - Targeting downstream mediators by rebastinib prolongs survival in preclinical models of glioblastoma. *Neuro Oncol* **2020**;22:345–56.
35. Canella A, Welker AM, Yoo JY, Xu J, Abas FS, Kesanakurti D, *et al.* Efficacy of Onalespib, a Long-Acting Second-Generation HSP90 Inhibitor, as a Single Agent and in Combination with Temozolomide against Malignant Gliomas. *Clin Cancer Res* **2017**;23:6215–26.
36. Cao J, Pontes KCS, Heijkants RC, Brouwer NJ, Groenewoud A, Jordanova ES, *et al*. Overexpression of EZH2 in conjunctival melanoma offers a new therapeutic target. *J Pathol* **2018**;245:433–44.
37. Cao HH, Liu DY, Lai YC, Chen YY, Yu LZ, Shao M, *et al.* Inhibition of the STAT3 Signaling Pathway Contributes to the Anti-Melanoma Activities of Shikonin. *Front Pharmacol* **2020**;11:748.
38. Carra S, Gaudenzi G, Dicitore A, Saronni D, Cantone MC, Plebani A, *et al*. Vandetanib versus cabozantinib in medullary thyroid carcinoma: A focus on anti-angiogenic effects in zebrafish model. *Int J Mol Sci* **2021**;22:1–17.
39. Carter RJ, Milani M, Butterworth M, Alotibi A, Harper N, Yedida G, *et al.* Exploring the potential of BH3 mimetic therapy in squamous cell carcinoma of the head and neck. *Cell Death Dis* **2019**;10:912.
40. Cecchini A, Raffa V, Canfarotta F, Signore G, Piletsky S, Macdonald MP, *et al.* In Vivo Recognition of Human Vascular Endothelial Growth Factor by Molecularly Imprinted Polymers. *Nano Lett* **2017**;17:2307–12.
41. Chang TC, Wei PL, Makondi PT, Chen WT, Huang CY, Chang YJ. Bromelain inhibits the ability of colorectal cancer cells to proliferate via activation of ROS production and autophagy. **PLoS One** 2019;14(1):e020274.
42. Chapman A, del Ama LF, Ferguson J, Kamarashev J, Wellbrock C, Hurlstone A.

Heterogeneous tumor subpopulations cooperate to drive invasion. *Cell Rep* **2014**;8:688–95.
43. Chen X, Wang J, Cao Z, Hosaka K, Jensen L, Yang H, *et al.* Invasiveness and metastasis of retinoblastoma in an orthotopic zebrafish tumor model. *Sci Rep* **2015**;5:10351.
44. Chen C, Choudhury S, Wangsa D, Lescott CJ, Wilkins DJ, Sripadhan P, *et al.* A multiplex preclinical model for adenoid cystic carcinoma of the salivary gland identifies regorafenib as a potential therapeutic drug. *Sci Rep* **2017**;7(1):11410.
45. Chen X, Fu Y, Xu H, Teng P, Xie Q, Zhang Y, *et al*. SOX5 predicts poor prognosis in lung adenocarcinoma and promotes tumor metastasis through epithelial-mesenchymal transition. *Oncotarget* **2018**;9:10891–904.
46. Chen P, Luo X, Che Z, Zhang W, Liu F, Hou D, *et al*. Targeting of the C-Jun/BCL-XL/P21 Axis Accelerates the Switch from Senescence to Apoptosis Upon ROC1 Knockdown in Gastric Cancer Cells. *Cell Physiol. Biochem* **2018**; 48(3):1123–38.
47. Chen HC, Rui W, You SY, Liu XW, Huang J, Chen HY. Evaluation of the anti-cervical cancer effect of a prodrug ：CBZ-AAN-DOX with hypoxic cell culture and tumor-bearing zebrafish models. *Exp Cell Res* **2020**;391(1):111980.
48. Chen L, De Menna M, Groenewoud A, Thalmann GN, Kruithof-de Julio M, Snaar-Jagalska BE. A NF-κB-Activin A signaling axis enhances prostate cancer metastasis. *Oncogene* **2020**;39:1634–51.
49. Cheng J, Gu Y-J, Wang Y, Cheng SH, Wong W-T. Nanotherapeutics in angiogenesis: synthesis and in vivo assessment of drug efficacy and biocompatibility in zebrafish embryos. *Int J Nanomedicine* **2011**;6:2007–21.
50. Chiu C-C, Chou H-L, Chen B-H, Chang K-F, Tseng C-H, Fong Y, *et al.* BPIQ, a novel synthetic quinoline derivative, inhibits growth and induces mitochondrial apoptosis of lung cancer cells in vitro and in zebrafish xenograft model. *BMC Cancer* **2015**;15:962.
51. Chou CK, Huang HW, Yang CF, Dahms HU, Liang SS, Wang TN, *et al*. Reduced camptothecin sensitivity of estrogen receptor-positive human breast cancer cells following exposure to di(2-ethylhexyl)phthalate (DEHP) is associated with DNA methylation changes. *Environ Toxicol* 2019;34:401–14.
52. Chung WP, Huang WL, Liao WA, Huang WL, Liu YY, Su WC. Development of the CK-MB-1 trastuzumab-resistant HER2-positive breast cancer cell line and xenograft animal models. *Cancer Med* **2021**;10:2370–9.
53. Corkery DP, Dellaire G, Berman JN. Leukaemia xenotransplantation in zebrafish - chemotherapy response assay in vivo. *Br J Haematol* **2011**;153:786–9.
54. Corkery DP, Clarke LE, Gebremeskel S, Salsman J, Pinder J, Le Page C, *et al.* Loss of PRP4K drives anoikis resistance in part by dysregulation of epidermal growth factor receptor endosomal trafficking. Oncogene. *Nature* **2018**;37:174–84.
55. Pitsillides CM, Runnels JM, Spencer JA, Zhi L, Wu MX, Lin CP. Cell labeling approaches for fluorescence-based in vivo flow cytometry. *Cytometry A* **2011**;79(10):758–65.
56. Czarnomysy R, Radomska D, Muszynska A, Hermanowicz JM, Prokop I, Bielawska A, *et al*. Evaluation of the Anticancer Activities of Novel Transition Metal Complexes with Berenil and Nitroimidazole. *Molecules*

2020;25(12):2860.
57. Das I, Gad H, Bräutigam L, Pudelko L, Tuominen R, Höiom V, *et al*. AXL and CAV-1 play a role for MTH1 inhibitor TH1579 sensitivity in cutaneous malignant melanoma. *Cell Death Differ* **2020**;27:2081–98.
58. Dawood M, Hegazy MEF, Elbadawi M, Fleischer E, Klinger A, Bringmann G, *et al*. Vitamin K3 chloro derivative (VKT-2) inhibits HDAC6, activates autophagy and apoptosis, and inhibits aggresome formation in hepatocellular carcinoma cells. *Biochem Pharmacol* 2020;180:114176.
59. Pontes KC de S, Groenewoud A, Cao J, Ataide LMS, Snaar-Jagalska E, Jager MJ. Evaluation of (fli:GFP) casper zebrafish embryos as a model for human conjunctival melanoma. *Investig Ophthalmol Vis Sci* **2017**;58:6065–71.
60. Delasoie J, Pavic A, Voutier N, Vojnovic S, Crochet A, Nikodinovic-Runic J, *et al*. Identification of novel potent and non-toxic anticancer, anti-angiogenic and antimetastatic rhenium complexes against colorectal carcinoma. *Eur J Med Chem* **2020**;204.
61. Deng LJ, Lei YH, Quan JY, Li BJ, Zhang DM, Tian HY, *et al.* 1β–OH–arenobufagin induces mitochondrial apoptosis in hepatocellular carcinoma through the suppression of mTOR signaling pathway. *J Ethnopharmacol* **2021**;266:113443.
62. Di Franco G, Usai A, Funel N, Palmeri M, Rosamaria Montesanti IE, Bianchini M, *et al.* Use of zebrafish embryos as avatar of patients with pancreatic cancer: A new xenotransplantation model towards personalized medicine. *World J Gastroenterol* **2020**;26:2792–809.
63. di Leo N, Battaglini M, Berger L, Giannaccini M, Dente L, Hampel S, *et al.* A catechin nanoformulation inhibits WM266 melanoma cell proliferation, migration and associated neo-angiogenesis. *Eur J Pharm Biopharm* **2017**;114:1–10.
64. Diamantopoulou Z, White G, Fadlullah MZH, Dreger M, Pickering K, Maltas J, et al. TIAM1 Antagonizes TAZ/YAP Both in the Destruction Complex in the Cytoplasm and in the Nucleus to Inhibit Invasion of Intestinal Epithelial Cells. *Cancer Cell* **2017**;31:621–34.
65. Dib S, Aggad D, Mauriello Jimenez C, Lakrafi A, Hery G, Nguyen C, *et al.* Porphyrin-based bridged silsesquioxane nanoparticles for targeted two-photon photodynamic therapy of zebrafish xenografted with human tumor. *Cancer Rep*2019;2(5):e1186.
66. Dicitore A, Cantone MC, Gaudenzi G, Saronni D, Carra S, Borghi MO, *et al*. Efficacy of a novel second-generation somatostatin-dopamine chimera (TBR-065) in human medullary thyroid cancer: a preclinical study. *Neuroendocrinology* **2020**:Online ahead of print.
67. Domenichini A, Casari I, Simpson P V., Desai NM, Chen L, Dustin C, *et al*. Rhenium N-heterocyclic carbene complexes block growth of aggressive cancers by inhibiting FGFR- and SRC-mediated signalling. *J Exp Clin Cancer Res* **2020**;39(1):276.
68. Drabsch Y, He S, Zhang L, Snaar-Jagalska BE, ten Dijke P. Transforming growth factor-β signalling controls human breast cancer metastasis in a zebrafish xenograft model. *Breast Cancer Res* **2013**;15:R106.
69. Du Q, Wang W, Liu T, Shang C, Huang J, Liao Y, *et al*. High Expression of Integrin α3 Predicts Poor Prognosis and Promotes Tumor Metastasis and Angiogenesis by Activating the c-Src/Extracellular Signal-Regulated Protein

Kinase/Focal Adhesion Kinase Signaling Pathway in Cervical Cancer. *Front Oncol* **2020**;10:36.
70. Dumartin L, Whiteman HJ, Weeks ME, Hariharan D, Dmitrovic B, Iacobuzio-Donahue CA, *et al.* AGR2 is a novel surface antigen that promotes the dissemination of pancreatic cancer cells through regulation of cathepsins B and D. *Cancer Res* **2011**;71:7091–102.
71. Eden CJ, Ju B, Murugesan M, Phoenix T. Orthotopic models of pediatric brain tumors in zebrafish. *Oncogene* **2015**;34:1736–42.
72. Eguiara A, Holgado O, Beloqui I, Abalde L, Sanchez Y, Callol C, *et al.* Xenografts in zebrafish embryos as a rapid functional assay for breast cancer stem-like cell identification. *Cell Cycle* **2011**;10:3751–7.
73. Fan Y, Xue W, Schachner M, Zhao W. Honokiol eliminates glioma/glioblastoma stem cell-like cells via JAK-STAT3 signaling and inhibits tumor progression by targeting epidermal growth factor receptor. *Cancers (Basel)* 2019;11(1):22.
74. Fenizia C, Bottino C, Corbetta S, Fittipaldi R, Floris P, Gaudenzi G, *et al.* SMYD3 promotes the epithelial-mesenchymal transition in breast cancer. *Nucleic Acids Res* **2019**;47:1278–93.
75. Fior R, Póvoa V, Mendes R V., Carvalho T, Gomes A, Figueiredo N, *et al.* Single-cell functional and chemosensitive profiling of combinatorial colorectal therapy in zebrafish xenografts. *Proc Natl Acad Sci U S A* **2017**;114:E8234–43.
76. Florean C, Schnekenburger M, Lee J-Y, Kim KR, Mazumder A, Song S, *et al.* Discovery and characterization of Isofistularin-3, a marine brominated alkaloid, as a new DNA demethylating agent inducing cell cycle arrest and sensitization to TRAIL in cancer cells. *Oncotarget* **2016**;7:24027–49.
77. Fornabaio G, Barnhill RL, Lugassy C, Bentolila LA, Cassoux N, Roman-Roman S, *et al.* Angiotropism and extravascular migratory metastasis in cutaneous and uveal melanoma progression in a zebrafish model. *Sci Rep* **2018**;8:1–12.
78. Franich AA, Živković MD, Ilić-Tomić T, Đorđević IS, Nikodinović-Runić J, Pavić A, *et al*. New minor groove covering DNA binding mode of dinuclear Pt(II) complexes with various pyridine-linked bridging ligands and dual anticancer-antiangiogenic activities. *J Biol Inorg Chem* **2020**;25:395–409.
79. Fu A, Peh YM, Ngan W, Wei N, Luo KQ. Rapid identification of antimicrometastases drugs using integrated model systems with two dimensional monolayer, three dimensional spheroids, and zebrafish xenotransplantation tumors. *Biotechnol Bioeng* **2018**;115:2828–43.
80. Gabellini C, Gómez-Abenza E, Ibáñez-Molero S, Tupone MG, Pérez-Oliva AB, de Oliveira S, *et al*. Interleukin 8 mediates bcl-xL-induced enhancement of human melanoma cell dissemination and angiogenesis in a zebrafish xenograft model. *Int J Cancer* **2018**;142:584–96.
81. Gacha-Garay MJ, Niño-Joya AF, Bolaños NI, Abenoza L, Quintero G, Ibarra H, *et al*. Pilot study of an integrative new tool for studying clinical outcome discrimination in acute leukemia. *Front Oncol* **2019**;9:245.
82. Gamble JT, Reed-Harris Y, Barton CL, La Du J, Tanguay R, Greenwood JA. Quantification of glioblastoma progression in zebrafish xenografts: Adhesion to laminin alpha 5 promotes glioblastoma microtumor formation and inhibits cell invasion. *Biochem Biophys Res Commun* **2018**;506:833–9.
83. Gao X, Yu T, Xu G, Guo G, Liu X, Hu X, *et al.* Enhancing the anti-glioma therapy of doxorubicin by honokiol with biodegradable self-assembling micelles

through multiple evaluations. *Sci Rep* **2017**;7:43501.
84. Gao Z, Deng G, Li Y, Huang H, Sun X, Shi H, *et al*. Actinidia chinensis Planch prevents proliferation and migration of gastric cancer associated with apoptosis, ferroptosis activation and mesenchymal phenotype suppression. *Biomed Pharmacother* **2020**;126:110092.
85. Gaudenzi G, Albertelli M, Dicitore A, Würth R, Gatto F, Barbieri F, *et al.* Patient-derived xenograft in zebrafish embryos: a new platform for translational research in neuroendocrine tumors. *Endocrine* **2017**;57:214–9.
86. Gaudenzi G, Vitale G. Transplantable zebrafish models of neuroendocrine tumors. *Ann Endocrinol (Paris)* **2019**;80:149–52.
87. Gauert A, Olk N, Pimentel-Gutiérrez H, Astrahantseff K, Jensen LD, Cao Y, *et al*. Fast, in vivo model for drug-response prediction in patients with b-cell precursor acute lymphoblastic leukemia. *Cancers (Basel)* **2020**;12:1–13.
88. Geiger GA, Fu W, Kao GD. Temozolomide-mediated radiosensitization of human glioma cells in a zebrafish embryonic system. *Cancer Res* **2008**;68:3396–404.
89. Ghotra VPS, He S, de Bont H, van der Ent W, Spaink HP, van de Water B, *et al.* Automated whole animal bio-imaging assay for human cancer dissemination. *PLoS One* **2012**;7:1–13.
90. Ghotra VPS, He S, van der Horst G, Nijhoff S, de Bont H, Lekkerkerker A, *et al.* SYK Is a Candidate Kinase Target for the Treatment of Advanced Prostate Cancer. *Cancer Res* **2015**;75:230–40.
91. Gianoncelli A, Guarienti M, Fragni M, Bertuzzi M, Rossini E, Abate A, *et al.* Adrenocortical Carcinoma Xenograft in Zebrafish Embryos as a Model To Study the In Vivo Cytotoxicity of Abiraterone Acetate. *Endocrinology* **2019**;160:2620–9.
92. Giunco S, Zangrossi M, Pozzolo FD, Celeghin A, Ballin G, Petrara MR, *et al.* Anti-proliferative and pro-apoptotic effects of short-term inhibition of telomerase in vivo and in human malignant b cells xenografted in zebrafish. *Cancers (Basel)* **2020**;12:1–16.
93. Gnosa S, Capodanno A, Murthy RV, Jensen LDE, Sun XF. AEG-1 knockdown in colon cancer cell lines inhibits radiation-enhanced migration and invasion in vitro and in a novel in vivo zebrafish model. *Oncotarget* **2016**;7:81634–44.
94. Goel S, Gupta N, Walcott BP, Snuderl M, Kesler CT, Kirkpatrick ND, *et al.* Effects of Vascular-Endothelial Protein Tyrosine Phosphatase Inhibition on Breast Cancer Vasculature and Metastatic Progression. *JNCI J Natl Cancer Inst* **2013**;105:1188–201.
95. Gonzalez-Torres C, Gaytan-Cervantes J, Vazquez-Santillan K, Mandujano-Tinoco EA, Ceballos-Cancino G, Garcia-Venzor A, *et al*. NF-κB Participates in the Stem Cell Phenotype of Ovarian Cancer Cells. *Arch Med Res* **2017**;48:343–51.
96. Greco MR, Bon E, Rubino R, Guerra L, Bernabe-Garcia M, Cannone S, *et al.* Phosphorylation of NHERF1 S279 and S301 differentially regulates breast cancer cell phenotype and metastatic organotropism. *Biochim Biophys Acta Mol Basis Dis* **2019**;1865:26–37.
97. Grolez GP, Hammadi M, Barras A, Gordienko D, Slomianny C, Völkel P, *et al.* Encapsulation of a TRPM8 Agonist, WS12, in Lipid Nanocapsules Potentiates PC3 Prostate Cancer Cell Migration Inhibition through Channel Activation. *Sci Rep* **2019**;9(1):7926.

98. Guo M, Wei H, Hu J, Sun S, Long J, Wang X. U0126 inhibits pancreatic cancer progression via the KRAS signaling pathway in a zebrafish xenotransplantation model. *Oncol Rep.* **2015**;34(2):699-706.
99. Guo Y, Fan Y, Pei X. Fangjihuangqi Decoction inhibits MDA-MB-231 cell invasion in vitro and decreases tumor growth and metastasis in triple-negative breast cancer xenografts tumor zebrafish model. *Cancer Med* **2020**;9:2564–78.
100. Haldi M, Ton C, Seng WL, McGrath P. Human melanoma cells transplanted into zebrafish proliferate, migrate, produce melanin, form masses and stimulate angiogenesis in zebrafish. *Angiogenesis* **2006**;9:139–51.
101. Hamilton L, Astell KR, Velikova G, Sieger D. A Zebrafish Live Imaging Model Reveals Differential Responses of Microglia Toward Glioblastoma Cells In Vivo. *Zebrafish* **2016**;13:523–34.
102. Hammarström LGJ, Harmel RK, Granath M, Ringom R, Gravenfors Y, Färnegårdh K, *et al.* The Oncolytic Efficacy and in Vivo Pharmacokinetics of [2-(4-Chlorophenyl)quinolin-4-yl](piperidine-2-yl)methanol (Vacquinol-1) Are Governed by Distinct Stereochemical Features. *J Med Chem* **2016**;59:8577–92.
103. Haney MG, Moore LH, Blackburn JS. Drug screening of primary patient derived tumor xenografts in zebrafish. *J Vis Exp* **2020**:158.
104. Hanna SJ, McCoy-Simandle K, Leung E, Genna A, Condeelis J, Cox D. Tunneling nanotubes, a novel mode of tumor cell-macrophage communication in tumor cell invasion. *J Cell Sci* **2019**;132(3):jcs223321.
105. Harfouche R, Basu S, Soni S, Hentschel DM, Mashelkar RA, Sengupta S. Nanoparticle-mediated targeting of phosphatidylinositol-3-kinase signaling inhibits angiogenesis. *Angiogenesis* **2009**;12:325–38.
106. He S, Lamers GE, Beenakker J-WM, Cui C, Ghotra VP, Danen EH, *et al.* Neutrophil-mediated experimental metastasis is enhanced by VEGFR inhibition in a zebrafish xenograft model. *J Pathol* **2012**;227:431–45.
107. He X, Yin X, Wu J, Wickström SL, Duo Y, Du Q, *et al.* Visualization of human T lymphocyte-mediated eradication of cancer cells in vivo. *Proc Natl Acad Sci U S A* **2020**;117:22910–9.
108. Hermanowicz JM, Szymanowska A, Sieklucka B, Czarnomysy R, Pawlak K, Bielawska A, *et al.* Exploration of novel heterofused 1,2,4-triazine derivative in colorectal cancer. *J Enzyme Inhib Med Chem* **2021**;36:535–48.
109. Hill D, Chen L, Snaar-Jagalska E, Chaudhry B. Embryonic zebrafish xenograft assay of human cancer metastasis. *F1000Research* **2018**;7:1682.
110. Hollenbach M, Stoll SJ, Jörgens K, Seufferlein T, Kroll J. Different Regulation of Physiological and Tumor Angiogenesis in Zebrafish by Protein Kinase D1 (PKD1). *PLoS One* **2013**;8:e68033.
111. Hong ZP, Wang LG, Wang HJ, Ye WF, Wang XZ. Wogonin exacerbates the cytotoxic effect of oxaliplatin by inducing nitrosative stress and autophagy in human gastric cancer cells. *Phytomedicine* **2018**;39:168–75.
112. Hsieh T-H, Hsu C-Y, Tsai C-F, Chiu C-C, Liang S-S, Wang T-N, *et al.* A novel cell-penetrating peptide suppresses breast tumorigenesis by inhibiting β-catenin/LEF-1 signaling. *Sci Rep* **2016**;6:19156.
113. Hsu DSS, Hwang WL, Yuh CH, Chu CH, Ho YH, Chen PB, *et al.* Lymphotoxin-β interacts with methylated EGFR to mediate acquired resistance to cetuximab in head and neck cancer. *Clin Cancer Res* **2017**;23:4388–401.

114. Hsu WJ, Lin MH, Kuo TC, Chou CM, Mi FL, Cheng CH, *et al.* Fucoidan from Laminaria japonica exerts antitumor effects on angiogenesis and micrometastasis in triple-negative breast cancer cells. *Int J Biol Macromol* **2020**;149:600–8.
115. Hu Y, Zhang M, Tian N, Li D, Wu F, Hu P, *et al*. The antibiotic clofoctol suppresses glioma stem cell proliferation by activating KLF13. *J Clin Invest* **2019**;129:3072–85.
116. Huang HW, Bow YD, Wang CY, Chen YC, Fu PR, Chang KF, *et al*. DFIQ, a novel quinoline derivative, shows anticancer potential by inducing apoptosis and autophagy in nsclc cell and in vivo zebrafish xenograft models. *Cancers (Basel)* **2020**;12(5):1348.
117. Hung AC, Lo S, Hou M-F, Lee Y-C, Tsai C-H, Chen Y-Y, *et al*. Extracellular Visfatin-Promoted Malignant Behavior in Breast Cancer Is Mediated Through c-Abl and STAT3 Activation. *Clin Cancer Res* **2016**;22:4478–90.
118. Ikonomopoulou MP, Fernandez-Rojo MA, Pineda SS, Cabezas-Sainz P, Winnen B, Morales RAV, *et al*. Gomesin inhibits melanoma growth by manipulating key signaling cascades that control cell death and proliferation. *Sci Rep* 2018;8(1):11519.
119. Iscan E, Ekin U, Yildiz G, Oz O, Keles U, Suner A, *et al*. TAp73β can promote hepatocellular carcinoma dedifferentiation. *Cancers (Basel)* **2021**;13:1–19.
120. Jakhar R, Luijten MNH, Wong AXF, Cheng B, Guo K, Neo SP, *et al*. Autophagy governs protumorigenic effects of mitotic slippage-induced senescence. Mol *Cancer Res* **2018**;16:1625–40.
121. Jelassi B, Anchelin M, Chamouton J, Cayuela ML, Clarysse L, Li J, *et al.* Anthraquinone emodin inhibits human cancer cell invasiveness by antagonizing P2X7 receptors. *Carcinogenesis* **2013**;34:1487–96.
122. Jerónimo A, Rodrigues G, Vilas-Boas F, Martins GG, Bagulho A, Real C. Hydrogen peroxide regulates angiogenesis-related factors in tumor cells. *Biochem Cell Biol* **2017**;95:679–85.
123. Ji S, Lee J-Y, Schrör J, Mazumder A, Jang DM, Chateauvieux S, *et al.* The dialkyl resorcinol stemphol disrupts calcium homeostasis to trigger programmed immunogenic necrosis in cancer. *Cancer Lett* **2018**;416:109–23.
124. Jin Y, Wei L, Jiang Q, Song X, Teng C, Fan C, *et al*. Comparison of efficacy and toxicity of bevacizumab, endostar and apatinib in transgenic and human lung cancer xenograftzebrafish model. *Sci Rep* **2018**;8(1):15837.
125. Jin W, Zhou L, Yan B, Yan L, Liu F, Tong P, *et al*. Theabrownin triggers DNA damage to suppress human osteosarcoma U2OS cells by activating p53 signalling pathway. *J Cell Mol Med* **2018**;22:4423–36.
126. Jin W, Gu C, Zhou L, Yang X, Gui M, Zhang J, *et al*. Theabrownin inhibits the cytoskeleton-dependent cell cycle, migration and invasion of human osteosarcoma cells through NF-κB pathway-related mechanisms. *Oncol Rep* **2020**;44:2621–33.
127. Jinendiran S, Teng W, Dahms HU, Liu W, Ponnusamy VK, Chiu CCC, *et al*. Induction of mitochondria-mediated apoptosis and suppression of tumor growth in zebrafish xenograft model by cyclic dipeptides identified from Exiguobacterium acetylicum. *Sci Rep* **2020**;10(1):13721.
128. Jo DH, Son D, Na Y, Jang M, Choi JH, Kim JH, *et al*. Orthotopic transplantation of retinoblastoma cells into vitreous cavity of zebrafish for screening of anticancer drugs. *Mol Cancer* **2013**;12:71.

129. Jung D-W, Kim J, Che ZM, Oh E-S, Kim G, Eom SH, *et al*. A Triazine Compound S06 Inhibits Proinvasive Crosstalk between Carcinoma Cells and Stromal Fibroblasts via Binding to Heat Shock Protein 90. *Chem Biol* **2011**;18:1581–90.
130. Jung DW, Oh ES, Park SH, Chang YT, Kim CH, Choi SY, *et al*. A novel zebrafish human tumor xenograft model validated for anti-cancer drug screening. *Mol Biosyst* **2012**;8:1930–9.
131. Jung D-W, Kim W-H, Park S-H, Lee J, Kim J, Su D, *et al.* A Unique Small Molecule Inhibitor of Enolase Clarifies Its Role in Fundamental Biological Processes. *ACS Chem Biol* **2013**;8:1271–82.
132. Kabakci Z, Käppeli S, Cantù C, Jensen LD, König C, Toggweiler J, *et al.* Pharmacophore-guided discovery of CDC25 inhibitors causing cell cycle arrest and tumor regression. *Sci Rep* **2019**;9(1):1335.
133. Karkampouna S, van der Helm D, Gray PC, Chen L, Klima I, Grosjean J, *et al*. CRIPTO promotes an aggressive tumour phenotype and resistance to treatment in hepatocellular carcinoma. *J Pathol* **2018**;245:297–310.
134. Kartha VK, Alamoud KA, Sadykov K, Nguyen BC, Laroche F, Feng H, *et al*. Functional and genomic analyses reveal therapeutic potential of targeting β-catenin/CBP activity in head and neck cancer. *Genome Med* **2018**;10:54.
135. Khan N, Mahajan NK, Sinha P, Jayandharan GR. An efficient method to generate xenograft tumor models of acute myeloid leukemia and hepatocellular carcinoma in adult zebrafish. *Blood Cells Mol Dis* **2019**;75:48–55.
136. Ki DH, Oppel F, Durbin AD, Look AT. Mechanisms underlying synergy between DNA topoisomerase I-targeted drugs and mTOR kinase inhibitors in NF1-associated malignant peripheral nerve sheath tumors. *Oncogene* **2019**;38:6585–98.
137. Kiener M, Chen L, Krebs M, Grosjean J, Klima I, Kalogirou C, et al. MiR-221-5p regulates proliferation and migration in human prostate cancer cells and reduces tumor growth in vivo. *BMC Cancer* **2019**;19:627.
138. Kim HK, Bhattarai KR, Junjappa RP, Ahn JH, Pagire SH, Yoo HJ, *et al.* TMBIM6/BI-1 contributes to cancer progression through assembly with mTORC2 and AKT activation. *Nat Commun* **2020**;11(1):4012.
139. Kim EA, Lee JH, Heo SJ, Jeon YJ. Saringosterol acetate isolated from Hizikia fusiforme, an edible brown alga, suppressed hepatocellular carcinoma growth and metastasis in a zebrafish xenograft model. *Chem Biol Interact* **2021**;335:109362.
140. Kong L, Chen Q, Campbell F, Snaar-Jagalska E, Kros A. Light-Triggered Cancer Cell Specific Targeting and Liposomal Drug Delivery in a Zebrafish Xenograft Model. *Adv Healthc Mater* **2020**;9(6):e1901489.
141. Lai YJ, Tsai JC, Tseng YT, Wu MS, Liu WS, Lam HI, *et al*. Small G protein Rac GTPases regulate the maintenance of glioblastoma stem-like cells in vitro and in vivo. *Oncotarget* **2017**;8:18031–49.
142. Lal S, La Du J, Tanguay RL, Greenwood JA. Calpain 2 Is Required for the Invasion of Glioblastoma Cells in the Zebrafish Brain Microenvironment. *J Neurosci Res* **2012**;90:769–81.
143. Lally BE, Geiger GA, Kridel S, Arcury-Quandt AE, Robbins ME, Kock ND, *et al.* Identification and biological evaluation of a novel and potent small molecule radiation sensitizer via an unbiased screen of a chemical library. *Cancer Res*

2007;67:8791–9.
144. Lara R, Mauri FA, Taylor H, Derua R, Shia A, Gray C, *et al*. An siRNA screen identifies RSK1 as a key modulator of lung cancer metastasis. *Oncogene* **2011**;30:3513–21.
145. Latifi A, Abubaker K, Castrechini N, Ward AC, Liongue C, Dobill F, *et al.* Cisplatin treatment of primary and metastatic epithelial ovarian carcinomas generates residual cells with mesenchymal stem cell-like profile. *J Cell Biochem* **2011**;112:2850–64.
146. Lee LMJ, Seftor EA, Bonde G, Cornell RA, Hendrix MJC. The fate of human malignant melanoma cells transplanted into zebrafish embryos: Assessment of migration and cell division in the absence of tumor formation. *Dev Dyn* **2005**;233:1560–70.
147. Lee SLC, Rouhi P, Dahl Jensen L, Zhang D, Ji H, Hauptmann G, *et al*. Hypoxia-induced pathological angiogenesis mediates tumor cell dissemination, invasion, and metastasis in a zebrafish tumor model. *Proc Natl Acad Sci U S A* **2009**;106:19485–90.
148. Lee H-J, Yang YJ, Jeong S, Lee JD, Choi S-Y, Jung D-W, *et al*. Development of a vestibular schwannoma xenograft zebrafish model for in vivo antitumor drug screening. *Laryngoscope* **2016**;126:E409–15.
149. Lee JY, Mazumder A, Diederich M. Preclinical assessment of the bioactivity of the anticancer coumarin OT48 by spheroids, colony formation assays, and zebrafish xenografts. *J Vis Exp* **2018**;2018:57490.
150. Lee JY, Bae H, Yang C, Park S, Youn BS, Kim HS, *et al.* Eupatilin promotes cell death by calcium influx through ER-mitochondria axis with SERPINB11 inhibition in epithelial ovarian cancer. *Cancers (Basel)* **2020**;12:1–20.
151. Leong KH, Mahdzir MA, Din MFM, Awang K, Tanaka Y, Kulkeaw K, *et al.* Induction of intrinsic apoptosis in leukaemia stem cells and in vivo zebrafish model by betulonic acid isolated from Walsura pinnata Hassk (Meliaceae). *Phytomedicine* **2017**;26:11–21.
152. Lernoux M, Schnekenburger M, Losson H, Vermeulen K, Hahn H, Gérard D, *et al*. Novel HDAC inhibitor MAKV-8 and imatinib synergistically kill chronic myeloid leukemia cells via inhibition of BCR-ABL/MYC-signaling: Effect on imatinib resistance and stem cells. *Clin Epigenetics* **2020**;12(1):69.
153. Leung AWY, Veinotte CJ, Melong N, Oh MH, Chen K, Enfield KSS, *et al*. In Vivo Validation of PAPSS1 (3'-phosphoadenosine 5'-phosphosulfate synthase 1) as a Cisplatin-sensitizing Therapeutic Target. *Clin Cancer Res* **2017**;23:6555–66.
154. Leung HW, Ko CH, Yue GGL, Herr I, Lau CBS. The natural agent 4-vinylphenol targets metastasis and stemness features in breast cancer stem-like cells. *Cancer Chemother Pharmacol* **2018**;82:185–97.
155. Li Y, Drabsch Y, Pujuguet P, Ren J, van Laar T, Zhang L, *et al*. Genetic depletion and pharmacological targeting of αv integrin in breast cancer cells impairs metastasis in zebrafish and mouse xenograft models. *Breast Cancer Res* **2015**;17:28.
156. Li L, Chen X, Liu CC, Lee LS, Man C, Cheng SH. Phytoestrogen Bakuchiol Exhibits In Vitro and In Vivo Anti-breast Cancer Effects by Inducing S Phase Arrest and Apoptosis. *Front Pharmacol* **2016**;7:128.
157. Li L, Liu CC, Chen X, Xu S, Cortes-Manno SH, Cheng SH. Mechanistic study of bakuchiol-induced anti-breast cancer stem cell and in vivo anti-metastasis effects. *Front Pharmacol* **2017**;8:746.

158. Li J, Li S, Guo J, Li Q, Long J, Ma C, *et al*. Natural Product Micheliolide (MCL) Irreversibly Activates Pyruvate Kinase M2 and Suppresses Leukemia. *J Med Chem* **2018**;61:4155–64.
159. Li XY, Huang LT, Wu JQ, He MF, Zhu SH, Zhan P, *et al*. Zebrafish Xenograft Model of Human Lung Cancer for Evaluating Osimertinib Resistance. *Biomed Res Int* **2019**;2019:3129748.
160. Li Y, Wu Q, Yu G, Li L, Zhao X, Huang X, *et al*. Polypyridyl Ruthenium(II) complex-induced mitochondrial membrane potential dissipation activates DNA damage-mediated apoptosis to inhibit liver cancer. *Eur J Med Chem* **2019**;164:282–91.
161. Li Y, Liu J, Wang Z, Jin J, Liu Y, Chen C, *et al*. Optimizing Energy Transfer in Nanostructures Enables In Vivo Cancer Lesion Tracking via Near-Infrared Excited Hypoxia Imaging. *Adv Mater* 2020;32(14):e1907718.
162. Liang Y, Zhang Q, Yang X, Li Y, Zhang X, Li Y, *et al.* Diterpenoids from the leaves of Casearia kurzii showing cytotoxic activities. *Bioorg Chem* **2020**;98:103741.
163. Liekens S, Noppen S, Gijsbers S, Sienaert R, Ronca R, Tobia C, *et al*. The broad-spectrum anti-DNA virus agent cidofovir inhibits lung metastasis of virus-independent, FGF2-driven tumors. *Oncotarget* **2015**;6:4633–48.
164. Lin J, Zhang W, Zhao JJ, Kwart AH, Yang C, Ma D, *et al*. A clinically relevant in vivo zebrafish model of human multiple myeloma to study preclinical therapeutic efficacy. *Blood* **2016**;128:249–52.
165. Lin QH, Qu W, Xu J, Feng F, He MF. 1-Methoxycarbony-β-carboline from Picrasma quassioides exerts anti-angiogenic properties in HUVECs in vitro and zebrafish embryos in vivo. *Chin J Nat Med* **2018**;16:599–609.
166. Lin CR, Chu TM, Luo A, Huang SJ, Chou HY, Lu MW, *et al*. Omega-3 polyunsaturated fatty acids suppress metastatic features of human cholangiocarcinoma cells by suppressing twist. *J Nutr Biochem* **2019**;74:108245.
167. Lin HS, Huang YL, Wang YR, Hsiao E, Hsu TA, Shiao HY, *et al*. Identification of novel anti-liver cancer small molecules with better therapeutic index than sorafenib via zebrafish drug screening platform. *Cancers (Basel)* **2019**;11(6):739.
168. Liu Y, Zhu P, Wang Y, Wei Z, Tao L, Zhu Z, *et al*. Antimetastatic therapies of the polysulfide diallyl trisulfide against triple-negative breast cancer (TNBC) via suppressing MMP2/9 by blocking NF-κB and ERK/MAPK signaling pathways. *PLoS One* **2015**;10(4):e0123781.
169. Liu W, Lo YL, Hsu C, Wu YT, Liao ZX, Wu WJ, *et al*. CS-PEI/Beclin-siRNA Downregulate Multidrug Resistance Proteins and Increase Paclitaxel Therapeutic Efficacy against NSCLC. *Mol Ther Nucleic Acids* **2019**;17:477–90.
170. Liu JS, Huo CY, Cao HH, Fan CL, Hu JY, Deng LJ, *et al*. Aloperine induces apoptosis and G2/M cell cycle arrest in hepatocellular carcinoma cells through the PI3K/Akt signaling pathway. *Phytomedicine* **2019**;61:152843.
171. Liu S, González-Prieto R, Zhang M, Geurink PP, Kooij R, Iyengar PV, *et al.* Deubiquitinase activity profiling identifies UCHL1 as a candidate oncoprotein that promotes TGFβ-induced breast cancer metastasis. *Clin Cancer Res* **2020**;26:1460–73.
172. Liu K, Xie F, Zhao T, Zhang R, Gao A, Chen Y, *et al*. Targeting SOX2 Protein with Peptide Aptamers for Therapeutic Gains against Esophageal Squamous Cell Carcinoma. *Mol Ther* **2020**;28:901–13.

173. Liu W, Wu CY, Lu MJ, Chuang YJ, Tsai EM, Leu S, *et al*. The Phenoxyphenol Compound 4-HPPP Selectively Induces Antiproliferation Effects and Apoptosis in Human Lung Cancer Cells through Aneupolyploidization and ATR DNA Repair Signaling. *Oxid Med Cell Longev* **2020**;2020:5167292.
174. Losson H, Gajulapalli SR, Lernoux M, Lee JY, Mazumder A, Gérard D, *et al*. The HDAC6 inhibitor 7b induces BCR-ABL ubiquitination and downregulation and synergizes with imatinib to trigger apoptosis in chronic myeloid leukemia. *Pharmacol Res* **2020**;160:105058.
175. Marques IJ, Weiss FU, Vlecken DH, Nitsche C, Bakkers J, Lagendijk AK, *et al*. Metastatic behaviour of primary human tumours in a zebrafish xenotransplantation model. *BMC Cancer* **2009**;9:1–14.
176. Marranci A, D'Aurizio R, Vencken S, Mero S, Guzzolino E, Rizzo M, *et al.* Systematic evaluation of the microRNAome through miR-CATCHv2.0 identifies positive and negative regulators of BRAF-X1 mRNA. *RNA Biol* **2019**;16:865–78.
177. Martín-Cortázar C, Chiodo Y, Raul Jiménez P, Bernabé M, Cayuela ML, Iglesias T, *et al.* CDCA7 finely tunes cytoskeleton dynamics to promote lymphoma migration and invasion. *Haematologica* **2020**;105:730–40.
178. Martín-Pardillos A, Valls Chiva Á, Bande Vargas G, Hurtado Blanco P, Piñeiro Cid R, Guijarro PJ, *et al.* The role of clonal communication and heterogeneity in breast cancer. *BMC Cancer* **2019**;19(1):666.
179. Martinez-Ordoñez A, Seoane S, Cabezas P, Eiro N, Sendon-Lago J, MacIa M, *et al*. Breast cancer metastasis to liver and lung is facilitated by Pit-1-CXCL12-CXCR4 axis. *Oncogene* **2018**;37:1430–44.
180. Matlapudi M, Moin A, Medishetti R, Rajendra K, Raichur A, Kumar BR. Dual Drug Conjugate Loaded Nanoparticles for the Treatment of Cancer. *Curr Drug Deliv* **2015**;12:782–94.
181. Mazumder A, Lee JY, Talhi O, Cerella C, Chateauvieux S, Gaigneaux A, *et al.* Hydroxycoumarin OT-55 kills CML cells alone or in synergy with imatinib or Synribo: Involvement of ER stress and DAMP release. *Cancer Lett* **2018**;438:197–218.
182. McCall R, Miles M, Lascuna P, Burney B, Patel Z, Sidoran KJ, *et al*. Dual targeting of the cancer antioxidant network with 1,4-naphthoquinone fused Gold(i) N-heterocyclic carbene complexes. *Chem Sci* **2017**;8:5918–29.
183. Mealiea D, Boudreau E, De Silva N, Okamoto L, Ho T, Fish JE, *et al*. Modeling oncolytic virus dynamics in the tumor microenvironment using zebrafish. *Cancer Gene Ther* **2020**; Online ahead of print.
184. Melong N, Steele S, MacDonald M, Holly A, Collins CC, Zoubeidi A, *et al.* Enzalutamide inhibits testosterone-induced growth of human prostate cancer xenografts in zebrafish and can induce bradycardia. *Sci Rep* **2017**;7:1–11.
185. Mercatali L, La Manna F, Groenewoud A, Casadei R, Recine F, Miserocchi G, *et al.* Development of a patient-derived xenograft (PDX) of breast cancer bone metastasis in a Zebrafish model. *Int J Mol Sci* **2016**;17:1–11.
186. Miserocchi G, Cocchi C, De Vita A, Liverani C, Spadazzi C, Calpona S, *et al.* Three-dimensional collagen-based scaffold model to study the microenvironment and drug-resistance mechanisms of oropharyngeal squamous cell carcinomas. *Cancer Biol Med* **2021**;Online ahead of print.
187. Montoro-García S, Alburquerque-González B, Bernabé-García Á, Bernabé-

García M, Rodrigues PC, Den-Haan H, *et al.* Novel anti-invasive properties of a Fascin1 inhibitor on colorectal cancer cells. *J Mol Med* **2020**;98:383–94.
188. Morgan E, Gamble JT, Pearce MC, Elson DJ, Tanguay RL, Kolluri SK, *et al.* Improved in vivo targeting of BCL-2 phenotypic conversion through hollow gold nanoshell delivery. *Apoptosis* **2019**;24(5-6):529-537.
189. Moshal KS, Ferri-Lagneau KF, Haider J, Pardhanani P, Leung T. Discriminating Different Cancer Cells Using a Zebrafish in Vivo Assay. *Cancers (Basel)* **2011**;3:4102–13.
190. Muthukumarasamy KM, Handore KL, Kakade DN, Shinde M V., Ranjan S, Kumar N, *et al*. Identification of noreremophilane-based inhibitors of angiogenesis using zebrafish assays. *Org Biomol Chem* **2016**;14:1569–78.
191. Nadar RA, Asokan N, Degli Esposti L, Curci A, Barbanente A, Schlatt L, *et al*. Preclinical evaluation of platinum-loaded hydroxyapatite nanoparticles in an embryonic zebrafish xenograft model. *Nanoscale* **2020**;12:13582–94.
192. Nakayama J, Lu JW, Makinoshima H, Gong Z. A novel zebrafish model of metastasis identifies the HSD11b1 inhibitor adrenosterone as a suppressor of epithelial-mesenchymal transition and metastatic dissemination. *Mol Cancer Res* **2020**;18:477–87.
193. Narasimhan S, Zulick ES, Novikov O, Parks AJ, Schlezinger JJ, Wang Z, *et al.* Towards resolving the pro-and anti-tumor effects of the aryl hydrocarbon receptor. *Int J Mol Sci* **2018**;19(5):1388.
194. Nešović M, Rankov AD, Podolski-Renić A, Nikolić I, Tasić G, Mancini A, *et al.* Src inhibitors pyrazolo[3,4-d]pyrimidines, si306 and pro-si306, inhibit focal adhesion kinase and suppress human glioblastoma invasion in vitro and in vivo. *Cancers (Basel)* **2020**;12:1–22.
195. Nicoli S, Ribatti D, Cotelli F, Presta M. Mammalian tumor xenograft induce neovascularization in zebrafish embryo. *Cancer Res* **2007**;67:2927–31.
196. Nouri M, Caradec J, Lubik AA, Li N, Hollier BG, Takhar M, *et al*. Therapy-induced developmental reprogramming of prostate cancer cells and acquired therapy resistance. *Oncotarget* **2017**;8:18949–67.
197. Ochoa-Alvarez JA, Krishnan H, Pastorino JG, Nevel E, Kephart D, Lee JJ, *et al*. Antibody and lectin target podoplanin to inhibit oral squamous carcinoma cell migration and viability by distinct mechanisms. *Oncotarget* **2015**;6:9045–60.
198. Oliveira BL, Stenton BJ, Unnikrishnan VB, De Almeida CR, Conde J, Negrão M, *et al*. Platinum-Triggered Bond-Cleavage of Pentynoyl Amide and N-Propargyl Handles for Drug-Activation. *J Am Chem Soc* **2020**;142:10869–80.
199. Olmer R, Engels L, Usman A, Menke S, Malik MNH, Pessler F, *et al*. Differentiation of Human Pluripotent Stem Cells into Functional Endothelial Cells in Scalable Suspension Culture. *Stem Cell Reports* **2018**;10:1657–72.
200. Olszewski MB, Pruszko M, Snaar-Jagalska E, Zylicz A, Zylicz M. Diverse and cancer type-specific roles of the p53 R248Q gain-of-function mutation in cancer migration and invasiveness. *Int J Oncol* **2019**;54:1168–82.
201. Othman N, Nagoor NH. MiR-608 regulates apoptosis in human lung adenocarcinoma via regulation of AKT2. *Int J Oncol* **2017**;51:1757–64.
202. Ouyang L, Zhang L, Liu J, Fu L, Yao D, Zhao Y, *et al*. Discovery of a Small-Molecule Bromodomain-Containing Protein 4 (BRD4) Inhibitor That Induces AMP-Activated Protein Kinase-Modulated Autophagy-Associated Cell Death in Breast Cancer. *J Med Chem* **2017**;60:9990–10012.
203. Pan Y, Zheng M, Zhong L, Yang J, Zhou S, Qin Y, *et al*. A Preclinical Evaluation

of SKLB261, a Multikinase Inhibitor of EGFR/Src/VEGFR2, as a Therapeutic Agent against Pancreatic Cancer. *Mol Cancer Ther* **2015**;14:407–18.
204. Parasido E, Avetian GS, Naeem A, Graham G, Pishvaian M, Glasgow E, *et al.* The sustained induction of c-MYC drives nab-paclitaxel resistance in primary pancreatic ductal carcinoma cells. *Mol Cancer Res* **2019**;17:1815–27.
205. Park J-H, Williams DR, Lee J-H, Lee S-D, Lee J-H, Ko H, *et al*. Potent Suppressive Effects of 1-Piperidinylimidazole Based Novel P2X7 Receptor Antagonists on Cancer Cell Migration and Invasion. *J Med Chem* **2016**;59:7410–30.
206. Pascoal S, Salzer B, Scheuringer E, Wenninger-Weinzierl A, Sturtzel C, Holter W, *et al*. A preclinical embryonic zebrafish xenograft model to investigate CAR T cells in vivo. *Cancers (Basel)* **2020**;12:1–14.
207. Pascoal S, Grissenberger S, Scheuringer E, Fior R, Ferreira MG, Distel M. Using Zebrafish Larvae as a Xenotransplantation Model to Study Ewing Sarcoma. *Methods Mol Biol* **2021**;2226:243-255.
208. Paul CD, Bishop K, Devine A, Paine EL, Staunton JR, Thomas SM, *et al*. Tissue Architectural Cues Drive Organ Targeting of Tumor Cells in Zebrafish. *Cell Syst* **2019**;9:187-206.e16.
209. Jang H, Pearce M, O'Donnell E, Nguyen B, Truong L, Mueller M, *et al.* Identification of a Raloxifene Analog That Promotes AhR-Mediated Apoptosis in Cancer Cells. *Biology (Basel)* **2017**;6(4):41.
210. Peerzade SAMA, Qin X, Laroche FJF, Palantavida S, Dokukin M, Peng B, *et al*. Ultrabright fluorescent silica nanoparticles for: In vivo targeting of xenografted human tumors and cancer cells in zebrafish. *Nanoscale* **2019**;11:22316–27.
211. Pekkonen P, Alve S, Balistreri G, Gramolelli S, Tatti-Bugaeva O, Paatero I, *et al.* Lymphatic endothelium stimulates melanoma metastasis and invasion via MMP14-dependent Notch3 and β1-integrin activation. *Elife* **2018**;7:e32490.
212. Peng B, Almeqdadi M, Laroche F, Palantavida S, Dokukin M, Roper J, *et al.* Data on ultrabright fluorescent cellulose acetate nanoparticles for imaging tumors through systemic and topical applications. *Data Br* **2019**;22:383–91.
213. Lv P, Ma D, Gao S, Zhang Y, Bae YK, Liang G, *et al*. Generation of foxn1/Casper Mutant Zebrafish for Allograft and Xenograft of Normal and Malignant Cells. *Stem Cell Reports* **2020**;15:749–60.
214. Peperstraete E, Lecerf C, Collette J, Vennin C, Raby L, Völkel P, *et al*. Enhancement of breast cancer cell aggressiveness by lncrna h19 and its mir-675 derivative: Insight into shared and different actions. *Cancers (Basel)* **2020**;12:1–20.
215. Petrović J, Glamočlija J, Ilić-Tomić T, Soković M, Robajac D, Nedić O, *et al.* Lectin from Laetiporus sulphureus effectively inhibits angiogenesis and tumor development in the zebrafish xenograft models of colorectal carcinoma and melanoma. *Int J Biol Macromol* **2020**;148:129–39.
216. Porta F, Ehrsam D, Lengerke C, Meyer Zu Schwabedissen HE. Synthesis and Characterization of PDMS-PMOXA-Based Polymersomes Sensitive to MMP-9 for Application in Breast Cancer. *Mol Pharm* **2018**;15:4884–97.
217. Poté N, Cros J, Laouirem S, Raffenne J, Negrão M, Albuquerque M, *et al*. The histone acetyltransferase hMOF promotes vascular invasion in hepatocellular carcinoma. *Liver Int* **2020**;40:956–67.
218. Póvoa V, Rebelo de Almeida C, Maia-Gil M, Sobral D, Domingues M, Martinez-Lopez M, *et al*. Innate immune evasion revealed in a colorectal zebrafish

xenograft model. *Nat Commun* **2021**;12:1–15.

219. Pruvot B, Jacquel A, Droin N, Auberger P, Bouscary D, Tamburini J, *et al.* Leukemic cell xenograft in zebrafish embryo for investigating drug efficacy. *Haematologica* **2011**;96:612–6.
220. Pudelko L, Edwards S, Balan M, Nyqvist D, Al-Saadi J, Dittmer J, *et al*. An orthotopic glioblastoma animal model suitable for high-throughput screenings. *Neuro Oncol* **2018**;20:1475–84.
221. Qin X, Laroche FFJ, Peerzade SAMA, Lam A, Sokolov I, Feng H. In vivo targeting of xenografted human cancer cells with functionalized fluorescent silica nanoparticles in zebrafish. *J Vis Exp* **2020**;(159):10.3791/61187.
222. Raimondi C, Calleja V, Ferro R, Fantin A, Riley AM, Potter BVL, *et al*. A Small Molecule Inhibitor of PDK1/PLCγ1 Interaction Blocks Breast and Melanoma Cancer Cell Invasion. *Sci Rep* **2016**;6:26142.
223. Rajan V, Melong N, Wong WH, King B, Spencer Tong R, Mahajan N, *et al.* Humanized zebrafish enhance human hematopoietic stem cell survival and promote acute myeloid leukemia clonal diversity. *Haematologica* **2020**;105:2391–9.
224. Rampazzo E, Persano L, Pistollato F, Moro E, Frasson C, Porazzi P, *et al*. Wnt activation promotes neuronal differentiation of Glioblastoma. *Cell Death Dis* **2013**;4:e500-14.
225. Ren J, Liu S, Cui C, Ten Dijke P. Invasive behavior of human breast cancer cells in embryonic zebrafish. *J Vis Exp* **2017**;(122):55459.
226. Ren J, Smid M, Iaria J, Salvatori DCF, Van Dam H, Zhu HJ, *et al*. Cancer-associated fibroblast-derived Gremlin 1 promotes breast cancer progression. *Breast Cancer Res* **2019**;21(1):109.
227. Ren J, Wang Y, Ware T, Iaria J, ten Dijke P, Zhu HJ. Reactivation of BMP signaling by suboptimal concentrations of MEK inhibitor and FK506 reduces organ-specific breast cancer metastasis. *Cancer Lett* **2020**;493:41–54.
228. Renziehausen A, Wang H, Rao B, Weir L, Nigro C Lo, Lattanzio L, *et al*. The renin angiotensin system (RAS) mediates bifunctional growth regulation in melanoma and is a novel target for therapeutic intervention. *Oncogene* **2019**;38:2320–36.
229. Roel M, Rubiolo JA, Guerra-Varela J, Silva SBL, Thomas OP, Cabezas-Sainz P, *et al*. Marine guanidine alkaloids crambescidins inhibit tumor growth and activate intrinsic apoptotic signaling inducing tumor regression in a colorectal carcinoma zebrafish xenograft model. *Oncotarget* **2016**;7:83071–87.
230. Ronca R, Ghedini GC, Maccarinelli F, Sacco A, Locatelli SL, Foglio E, *et al*. FGF trapping inhibits multiple myeloma growth through c-Myc degradation–induced mitochondrial oxidative stress. *Cancer Res* **2020**;80:2340–54.
231. Rouhi P, Jensen LD, Cao Z, Hosaka K, Länne T, Wahlberg E, *et al*. Hypoxia-induced metastasis model in embryonic zebrafish. *Nat Protoc* **2010**;5:1911–8.
232. Sacco A, Roccaro AM, Ma D, Shi J, Mishima Y, Moschetta M, *et al*. Cancer Cell Dissemination and Homing to the Bone Marrow in a Zebrafish Model. *Cancer Res* **2016**;76:463–71.
233. Satapathy SR, Sjölander A. Cysteinyl leukotriene receptor 1 promotes 5-fluorouracil resistance and resistance-derived stemness in colon cancer cells. *Cancer Lett* **2020**;488:50–62.

234. Satapathy SR, Topi G, Osman J, Hellman K, Ek F, Olsson R, *et al*. Tumour suppressor 15-hydroxyprostaglandin dehydrogenase induces differentiation in colon cancer via GLI1 inhibition. *Oncogenesis* **2020**;9(8):74.
235. Savio M, Ferraro D, Maccario C, Vaccarone R, Jensen LD, Corana F, *et al.* Resveratrol analogue 4,4′-dihydroxy-trans-stilbene potently inhibits cancer invasion and metastasis. *Sci Rep* **2016**;6:19973.
236. Schaefer T, Wang H, Mir P, Konantz M, Pereboom TC, Paczulla AM, *et al.* Molecular and functional interactions between AKT and SOX2 in breast carcinoma. *Oncotarget* **2015**;6:43540–56.
237. Schneider NFZ, Cerella C, Lee JY, Mazumder A, Kim KR, de Carvalho A, *et al.* Cardiac glycoside glucoevatromonoside induces cancer type-specific cell death. *Front Pharmacol* 2018;9:70.
238. Schnekenburger M, Goffin E, Lee J-Y, Jang JY, Mazumder A, Ji S, *et al*. Discovery and Characterization of R / S - N -3-Cyanophenyl- N ′-(6- tert - butoxycarbonylamino-3,4-dihydro-2,2-dimethyl-2 H -1-benzopyran-4-yl)urea, a New Histone Deacetylase Class III Inhibitor Exerting Antiproliferative Activity against Cancer Cell Lines*. J Med Chem* **2017**;60:4714–33.
239. Selvaraju K, Mofers A, Pellegrini P, Salomonsson J, Ahlner A, Morad V, *et al.* Cytotoxic unsaturated electrophilic compounds commonly target the ubiquitin proteasome system. *Sci Rep* **2019**;9:1–17.
240. Sensi F, D'angelo E, Piccoli M, Pavan P, Mastrotto F, Caliceti P, *et al.* Recellularized colorectal cancer patient-derived scaffolds as in vitro pre-clinical 3D model for drug screening. *Cancers (Basel)* **2020**;12(3):681.
241. Sahgal P, Alanko J, Icha J, Paatero I, Hamidi H, Arjonen A, *et al*. GGA2 and RAB13 promote activity-dependent β1-integrin recycling. *J Cell Sci* **2019**;132(11):jcs233387.
242. Sharif GM, Schmidt MO, Yi C, Hu Z, Haddad BR, Glasgow E, *et al.* Cell growth density modulates cancer cell vascular invasion via Hippo pathway activity and CXCR2 signaling. *Oncogene* **2015**;34:5879–89.
243. Shen J, Najafi S, Stäble S, Fabian J, Koeneke E, Kolbinger FR, *et al*. A kinome-wide RNAi screen identifies ALK as a target to sensitize neuroblastoma cells for HDAC8-inhibitor treatment. *Cell Death Differ* **2018**;25:2053–70.
244. Shen R, Xu P, Wang N, Yi H, Dong L, Fu D, *et al.* Influence of oncogenic mutations and tumor microenvironment alterations on extranodal invasion in diffuse large B-cell lymphoma. *Clin Transl Med* **2020**;10(7):e221.
245. Shen J, Zhai J, You Q, Zhang G, He M, Yao X, *et al.* Cancer-associated fibroblasts-derived VCAM1 induced by H. pylori infection facilitates tumor invasion in gastric cancer. *Oncogene* **2020**;39:2961–74.
246. Shen W, Pu J, Sun J, Tan B, Wang W, Wang L, *et al.* Zebrafish xenograft model of human lung cancer for studying the function of LINC00152 in cell proliferation and invasion. *Cancer Cell Int* **2020**;20:376.
247. Shi X, Zhang T, Lou H, Song H, Li C, Fan P. Anticancer Effects of Honokiol via Mitochondrial Dysfunction Are Strongly Enhanced by the Mitochondria-Targeting Carrier Berberine. *J Med Chem* **2020**;63:11786–800.
248. Shtam T, Naryzhny S, Samsonov R, Karasik D, Mizgirev I, Kopylov A, *et al*. Plasma exosomes stimulate breast cancer metastasis through surface interactions and activation of FAK signaling. *Breast Cancer Res Treat* **2019**;174:129–41.

249. Sittaramane V, Padgett J, Salter P, Williams A, Luke S, McCall R, *et al.* Discovery of Quinoline-Derived Trifluoromethyl Alcohols, Determination of Their in vivo Toxicity and Anticancer Activity in a Zebrafish Embryo Model. *Chem Med Chem* **2015**;10:1802–7.
250. Slater K, Heeran AB, Garcia-Mulero S, Kalirai H, Sanz-Pamplona R, Rahman A, *et al.* High cysteinyl leukotriene receptor 1 expression correlates with poor survival of uveal melanoma patients and cognate antagonist drugs modulate the growth, cancer secretome, and metabolism of uveal melanoma cells. *Cancers (Basel)* **2020**;12:1–27.
251. Smith MP, Ferguson J, Arozarena I, Hayward R, Marais R, Chapman A, *et al.* Effect of SMURF2 Targeting on Susceptibility to MEK Inhibitors in Melanoma. *JNCI J Natl Cancer Inst* **2013**;105:33–46.
252. Smith MP, Rowling EJ, Miskolczi Z, Ferguson J, Spoerri L, Haass NK, *et al.* Targeting endothelin receptor signalling overcomes heterogeneity driven therapy failure. *EMBO Mol Med* **2017**;9:1011–29.
253. Somasagara RR, Huang X, Xu C, Haider J, Serody JS, Armistead PM, *et al.* Targeted therapy of human leukemia xenografts in immunodeficient zebrafish. *Sci Rep* **2021**;11(1):5715.
254. Stoletov K, Montel V, Lester RD, Gonias SL, Klemke R. High-resolution imaging of the dynamic tumor cell vascular interface in transparent zebrafish. *Proc Natl Acad Sci* **2007**;104:17406–11.
255. Stoletov K, Kato H, Zardouzian E, Kelber J, Yang J, Shattil S, *et al.* Visualizing extravasation dynamics of metastatic tumor cells. *J Cell Sci* **2010**;123:2332–41.
256. Sun DY, Wu JQ, He ZH, He MF, Sun H Bin. Cancer-associated fibroblast regulate proliferation and migration of prostate cancer cells through TGF-β signaling pathway. *Life Sci* **2019**;235:116791.
257. Tan DS, Haaland B, Gan J, Tham S, Sinha I, Tan E, *et al.* Bosutinib inhibits migration and invasion via ack1 in kras mutant non-small cell lung cancer. *Mol Cancer* **2014**;13:13.
258. Tan C, Sun W, Xu Z, Zhu S, Hu W, Wang X, *et al.* Small extracellular vesicles deliver TGF-β1 and promote adriamycin resistance in breast cancer cells. *Mol Oncol* **2021**;Online ahead of print.
259. Tandon A, Birkenhagen J, Nagalla D, Kölker S, Sauer SW. ADP-dependent glucokinase as a novel onco-target for haematological malignancies. *Sci Rep* **2020**;10(1):13584.
260. Tang JY, Xu YH, Lin LC, Ou-Yang F, Wu KH, Tsao LY, *et al.* LY303511 displays antiproliferation potential against oral cancer cells in vitro and in vivo. *Environ Toxicol* **2019**;34:958–67.
261. Teng Y, Xie X, Walker S, White DT, Mumm JS, Cowell JK. Evaluating human cancer cell metastasis in zebrafish. *BMC Cancer* **2013**;13:453.
262. Tian L, Xie K, Sheng D, Wan X, Zhu G. Antiangiogenic effects of oridonin. *BMC Complement Altern Med* **2017**;17:192.
263. Tiek DM, Khatib SA, Trepicchio CJ, Heckler MM, Divekar SD, Sarkaria JN, *et al.* Estrogen-related receptor β activation and isoform shifting by cdc2-like kinase inhibition restricts migration and intracranial tumor growth in glioblastoma. *FASEB J* **2019**;33:13476–91.
264. Timmermans-Sprang EPM, Mestemaker HM, Steenlage RR, Mol JA. Dasatinib inhibition of cSRC prevents the migration and metastasis of canine mammary

cancer cells with enhanced Wnt and HER signalling. *Vet Comp Oncol* **2019**;17:413–26.
265. Tomko A, O'Leary L, Trask H, Achenbach JC, Hall SR, Goralski KB, *et al*. Antitumor activity of abnormal cannabidiol and its analog O-1602 in taxol-resistant preclinical models of breast cancer. *Front Pharmacol* **2019**;10:1124.
266. Tonon F, Zennaro C, Dapas B, Carraro M, Mariotti M, Grassi G. Rapid and cost-effective xenograft hepatocellular carcinoma model in Zebrafish for drug testing. *Int J Pharm* **2016**;515:583–91.
267. Topczewska JM, Postovit LM, Margaryan NV, Sam A, Hess AR, Wheaton WW, *et al*. Embryonic and tumorigenic pathways converge via Nodal signaling: Role in melanoma aggressiveness. *Nat Med* **2006**;12:925–32.
268. Topi G, Satapathy SR, Dash P, Fred Mehrabi S, Ehrnström R, Olsson R, *et al.* Tumour-suppressive effect of oestrogen receptor β in colorectal cancer patients, colon cancer cells, and a zebrafish model. *J Pathol* **2020**;251:297–309.
269. Tseng C-H, Chen Y-R, Tzeng C-C, Liu W, Chou C-K, Chiu C-C, *et al*. Discovery of indeno[1,2- b]quinoxaline derivatives as potential anticancer agents. *Eur J Med Chem* **2016**;108:258–73.
270. Tseng LL, Cheng HH, Yeh T Sen, Huang SC, Syu YY, Chuu CP, *et al.* Targeting the histone demethylase PHF8-mediated PKCα-Src-PTEN axis in HER2-negative gastric cancer. *Proc Natl Acad Sci U S A* **2020**;117:24859–66.
271. Tsering J, Hu X. Triphala Suppresses Growth and Migration of Human Gastric Carcinoma Cells in Vitro and in a Zebrafish Xenograft Model. *Biomed Res Int* **2018**;2018:7046927.
272. Tu WM, Huang XC, Chen YL, Luo YL, Liau I, Hsu HY. Longitudinal and quantitative assessment platform for concurrent analysis of anti-tumor efficacy and cardiotoxicity of nano-formulated medication in vivo. *Anal Chim Acta* **2020**;1095:129–37.
273. Tulotta C, Stefanescu C, Beletkaia E, Bussmann J, Tarbashevich K, Schmidt T, *et al*. Inhibition of signaling between human CXCR4 and zebrafish ligands by the small molecule IT1t impairs the formation of triple-negative breast cancer early metastases in a zebrafish xenograft model. *Dis Model Mech* **2016**;9:141–53.
274. Tulotta C, Stefanescu C, Chen Q, Torraca V, Meijer AH, Snaar-Jagalska BE. CXCR4 signaling regulates metastatic onset by controlling neutrophil motility and response to malignant cells. *Sci Rep* **2019**;9(1):2399.
275. Usai A, Di Franco G, Colucci P, Pollina LE, Vasile E, Funel N, *et al*. A model of a zebrafish avatar for co-clinical trials. *Cancers (Basel)* **2020**;12(3):677.
276. Valenti MT, Marchetto G, Perduca M, Tiso N, Mottes M, Carbonare LD. Bel β-trefoil reduces the migration ability of RUNX2 expressing melanoma cells in xenotransplanted zebrafish. *Molecules* **2020**;25(6):1270.
277. van der Ent W, Jochemsen AG, Teunisse AF, Krens SG, Szuhai K, Spaink HP, *et al.* Ewing sarcoma inhibition by disruption of EWSR1-FLI1 transcriptional activity and reactivation of p53. *J Pathol* **2014**;233:415–24.
278. van der Ent W, Burrello C, Teunisse A, Ksander B, van der Velden PA.; Jager M, *et al.* Modeling of human uveal melanoma in zebrafish xenograft embryos. *Investig Opthalmology Vis Sci* **2014**;55:6612–22.
279. Varanda AB, Martins-Logrado A, Ferreira MG, Fior R. Zebrafish xenografts unveil sensitivity to olaparib beyond BRCA status. *Cancers (Basel)* **2020**;12:1–

20.
280. Varas MA, Muñoz-Montecinos C, Kallens V, Simon V, Allende ML, Marcoleta AE, *et al*. Exploiting Zebrafish Xenografts for Testing the in vivo Antitumorigenic Activity of Microcin E492 Against Human Colorectal Cancer Cells. *Front Microbiol* **2020**;11:405.
281. Vargas-Patron LA, Agudelo-Dueñãs N, Madrid-Wolff J, Venegas JA, González JM, Forero-Shelton M, *et al.* Xenotransplantation of human glioblastoma in Zebrafish larvae: In vivo imaging and proliferation assessment. *Biol Open* **2019**;8(5):bio043257.
282. Rodriguez GV, Abrahamsson A, Jensen LDE, Dabrosin C. Estradiol promotes breast cancer cell migration via recruitment and activation of neutrophils. *Cancer Immunol Res* **2017**;5:234–47.
283. Vazquez Rodriguez G, Abrahamsson A, Jensen LDE, Dabrosin C. Adipocytes Promote Early Steps of Breast Cancer Cell Dissemination via Interleukin-8. *Front Immunol* **2018**;9:1767.
284. Vervoort SJ, de Jong OG, Guy Roukens M, Frederiks CL, Vermeulen JF, Lourenço AR, *et al.* Global transcriptional analysis identifies a novel role for SOX4 in tumor-induced angiogenesis. *Elife* **2018**;7:e27706.
285. Verykiou S, Alexander M, Edwards N, Plummer R, Chaudhry B, Lovat PE, *et al*. Harnessing autophagy to overcome mitogen-activated protein kinase kinase inhibitor-induced resistance in metastatic melanoma. Br J Dermatol **2019**;180:346–56.
286. Vicente C, Arriazu E, Martínez-Balsalobre E, Peris I, Marcotegui N, García-Ramírez P, *et al.* A novel FTY720 analogue targets SET-PP2A interaction and inhibits growth of acute myeloid leukemia cells without inducing cardiac toxicity. *Cancer Lett* **2020**;468:1–13.
287. Vittori M, Breznik B, Gredar T, Hrovat K, Bizjak Mali L, Lah TT. Imaging of human glioblastoma cells and their interactions with mesenchymal stem cells in the zebrafish (Danio rerio) embryonic brain. *Radiol Oncol* **2016**;50:159–67.
288. Vittori M, Breznik B, Hrovat K, Kenig S, Lah TT. RECQ1 helicase silencing decreases the tumour growth rate of U87 glioblastoma cell xenografts in zebrafish embryos. *Genes (Basel)* **2017**;8(9):22.
289. Vlecken DH, Bagowski CP. LIMK1 and LIMK2 Are Important for Metastatic Behavior and Tumor Cell-Induced Angiogenesis of Pancreatic Cancer Cells. *Zebrafish* **2009**;6:433–9.
290. von Mässenhausen A, Sanders C, Brägelmann J, Konantz M, Queisser A, Vogel W, et al. Targeting DDR2 in head and neck squamous cell carcinoma with dasatinib. *Int J Cancer* **2016**;139:2359–69.
291. Wagner DS, Delk NA, Lukianova-Hleb EY, Hafner JH, Farach-Carson MC, Lapotko DO. The in vivo performance of plasmonic nanobubbles as cell theranostic agents in zebrafish hosting prostate cancer xenografts. *Biomaterials* **2010**;31:7567–74.
292. Wang Y-C, Wu Y-N, Wang S-L, Lin Q-H, He M-F, Liu Q, *et al.* Docosahexaenoic Acid Modulates Invasion and Metastasis of Human Ovarian Cancer via Multiple Molecular Pathways. *Int J Gynecol Cancer* **2016**;26:994–1003.
293. Wang J, Kuang B, Guo X, Liu J, Ding Y, Li J, *et al*. Total Syntheses and Biological Activities of Vinylamycin Analogues. *J Med Chem* **2017**;60:1189–209.
294. Wang G, Zhou P, Chen X, Zhao L, Tan J, Yang Y, *et al.* The novel autophagy inhibitor elaiophylin exerts antitumor activity against multiple myeloma with

mutant TP53 in part through endoplasmic reticulum stress-induced apoptosis. *Cancer Biol Ther* **2017**;18:584–95.
295. Wang H, Schaefer T, Konantz M, Braun M, Varga Z, Paczulla AM, *et al.* Prominent oncogenic roles of EVI1 in breast carcinoma. *Cancer Res* **2017**;77:2148–60.
296. Wang CH, Wang PJ, Hsieh YC, Lo S, Lee YC, Chen YC, *et al*. Resistin facilitates breast cancer progression via TLR4- mediated induction of mesenchymal phenotypes and stemness properties. *Oncogene* **2018**;37:589–600.
297. Wang L, Chen H, Fei F, He X, Sun S, Lv K, *et al*. Patient-derived Heterogeneous Xenograft Model of Pancreatic Cancer Using Zebrafish Larvae as Hosts for Comparative Drug Assessment. *J Vis Exp* **2019**;146.
298. Wang S, Hwang EE, Guha R, O'Neill AF, Melong N, Veinotte CJ, *et al*. High-throughput chemical screening identifies focal adhesion Kinase and Aurora Kinase B inhibition as a synergistic treatment combination in ewing sarcoma. *Clin Cancer Res* **2019**;25:4552–66.
299. Wang S, Wang N, Huang X, Yang B, Zheng Y, Zhang J, *et al*. Baohuoside i suppresses breast cancer metastasis by downregulating the tumor-associated macrophages/C-X-C motif chemokine ligand 1 pathway. *Phytomedicine* **2020**;78.
300. Wang Y, Ma Q, Zhang S, Liu H, Zhao B, Du B, et al. Digoxin Enhances the Anticancer Effect on Non-Small Cell Lung Cancer While Reducing the Cardiotoxicity of Adriamycin. *Front Pharmacol* **2020**;78:153331.
301. Wang N, Muhetaer G, Zhang X, Yang B, Wang C, Zhang Y, *et al*. Sanguisorba officinalis L. Suppresses Triple-Negative Breast Cancer Metastasis by Inhibiting Late-Phase Autophagy via Hif-1α/Caveolin-1 Signaling. *Front Pharmacol* **2020**;11:591400.
302. Wang SW, Lee CH, Lin MS, Chi CW, Chen YJ, Wang GS, *et al.* ZnO nanoparticles induced caspase-dependent apoptosis in gingival squamous cell carcinoma through mitochondrial dysfunction and p70s6K signaling pathway. *Int J Mol Sci* **2020**;21(5):1612.
303. Wang L, Liu Y, Liu H, Tian H, Wang Y, Zhang G, *et al*. The therapeutic significance of the novel photodynamic material TPE-IQ-2O in tumors. *Aging (Albany NY)* **2021**;13:1383–409.
304. Wang L, Long J, Chen H, Sun S, Lv K, Li Q, *et al.* Manipulation of focal Wnt activity via synthetic cells in a double-humanized zebrafish model of tumorigenesis. *Int J Cancer* **2021**;148:2815–24.
305. Wang S, Han C, Liu T, Ma Z, Qiu M, Wang J, *et al.* FAM83H-AS1 is a noncoding oncogenic driver and therapeutic target of lung adenocarcinoma. *Clin Transl* **2021**;11(2):e316.
306. Wäster P, Orfanidis K, Eriksson I, Rosdahl I, Seifert O, Öllinger K. UV radiation promotes melanoma dissemination mediated by the sequential reaction axis of cathepsins-TGF-β1-FAP-α. *Br J Cancer* **2017**;117:535–44.
307. Wehmas LC, Tanguay RL, Punnoose A, Greenwood JA. Developing a Novel Embryo–Larval Zebrafish Xenograft Assay to Prioritize Human Glioblastoma Therapeutics. *Zebrafish* **2016**;13:317–29.
308. Wei Z, Shan Y, Tao L, Liu Y, Zhu Z, Liu Z, *et al.* Diallyl trisulfides, a natural histone deacetylase inhibitor, attenuate HIF-1α synthesis, and decreases breast cancer metastasis. *Mol Carcinog* **2017**;56:2317–31.
309. Wei PL, Huang CY, Chang YJ. Propyl gallate inhibits hepatocellular carcinoma

cell growth through the induction of ROS and the activation of autophagy. *PLoS One* **2019**;14(1):e0210513.
310. Weijts BGMW, Westendorp B, Hien BT, Martínez-López LM, Zijp M, Thurlings I, *et al.* Atypical E2Fs inhibit tumor angiogenesis. *Oncogene* **2018**;37:271–6.
311. Weiss FU, Marques IJ, Woltering JM, Vlecken DH, Aghdassi A, Partecke LI, **et al**. Retinoic Acid Receptor Antagonists Inhibit miR-10a Expression and Block Metastatic Behavior of Pancreatic Cancer. *Gastroenterology* **2009**;137:2136-2145.e7.
312. Welker AM, Jaros BD, Puduvalli VK, Imitola J, Kaur B, Beattie CE. Standardized orthotopic xenografts in zebrafish reveal glioma cell-line-specific characteristics and tumor cell heterogeneity. *DMM Dis Model Mech* **2016**;9:199–210.
313. Welker AM, Jaros BD, An M, Beattie CE. Changes in tumor cell heterogeneity after chemotherapy treatment in a xenograft model of glioblastoma. *Neuroscience* **2017**;356:35–43.
314. Wen J, Yin P, Li L, Kang G, Ning G, Cao Y, *et al.* Knockdown of Matrix Metallopeptidase 9 Inhibits Metastasis of Oral Squamous Cell Carcinoma Cells in a Zebrafish Xenograft Model. *Biomed Res Int* **2020**;2020:4350783.
315. Wenger A, Larsson S, Danielsson A, Elbæk KJ, Kettunen P, Tisell M, *et al*. Stem cell cultures derived from pediatric brain tumors accurately model the originating tumors. *Oncotarget* **2017**;8:18626–39.
316. Wong CW, Han HW, Tien YW, Hsu S hui. Biomaterial substrate-derived compact cellular spheroids mimicking the behavior of pancreatic cancer and microenvironment. *Biomaterials* **2019**;213:119202.
317. Wong CW, Zhilenkov A V., Kraevaya OA, Mischenko D V., Troshin PA, Hsu SH. Toward understanding the antitumor effects of water-soluble fullerene derivatives on lung cancer cells: apoptosis or autophagy pathways? *J Med Chem* **2019**;62:7111–25.
318. Wrobel JK, Najafi S, Ayhan S, Gatzweiler C, Krunic D, Ridinger J, *et al*. Rapid in vivo validation of hdac inhibitor-based treatments in neuroblastoma zebrafish xenografts. *Pharmaceuticals* **2020**;13:1–20.
319. Wu Q, Deng S, Li L, Sun L, Yang X, Liu X, *et al.* Biodegradable polymeric micelle-encapsulated quercetin suppresses tumor growth and metastasis in both transgenic zebrafish and mouse models. *Nanoscale* **2013**;5:12480.
320. Wu JQ, Zhai J, Li CY, Tan AM, Wei P, Shen LZ, *et al.* Patient-derived xenograft in zebrafish embryos: A new platform for translational research in gastric cancer. *J Exp Clin Cancer Res* **2017**;36:160.
321. Wu Q, Zheng K, Huang X, Li L, Mei W. Tanshinone-IIA-Based Analogues of Imidazole Alkaloid Act as Potent Inhibitors to Block Breast Cancer Invasion and Metastasis in Vivo. *J Med Chem* **2018**;61:10488–501.
322. Wu XX, Yue GGL, Dong JR, Lam CWK, Wong CK, Qiu MH, *et al.* Actein Inhibits the Proliferation and Adhesion of Human Breast Cancer Cells and Suppresses Migration in vivo. *Front Pharmacol* **2018**;9:1–13.
323. Wu L, Chen P, Ying J, Zhang Q, Liu F, Lv B, *et al*. MAT2B mediates invasion and metastasis by regulating EGFR signaling pathway in hepatocellular carcinoma. *Clin Exp Med* **2019**;19:535–46.
324. Wu Q, Song Y, Liu R, Wang R, Mei W, Chen W, *et al.* Synthesis, docking studies and antitumor activity of phenanthroimidazole derivatives as promising c-myc G-quadruplex DNA stabilizers. *Bioorg Chem* **2020**;102:104074.

325. Wu JQ, Fan RY, Zhang SR, Li CY, Shen LZ, Wei P, *et al.* A systematical comparison of anti-angiogenesis and anti-cancer efficacy of ramucirumab, apatinib, regorafenib and cabozantinib in zebrafish model. *Life Sci* **2020**;247:117402.
326. Xie Z, Guo W, Guo N, Huangfu M, Liu H, Lin M, *et al.* Targeting tumor hypoxia with stimulus-responsive nanocarriers in overcoming drug resistance and monitoring anticancer efficacy. *Acta Biomater* **2018**;71:351–62.
327. Xu J, Zhu X, Qiu L. Polyphosphazene vesicles for co-delivery of doxorubicin and chloroquine with enhanced anticancer efficacy by drug resistance reversal. *Int J Pharm* **2016**;498:70–81.
328. Xu W, Foster BA, Richards M, Bondioli KR, Shah G, Green CC. Characterization of prostate cancer cell progression in zebrafish xenograft model. *Int J Oncol* **2018**;52:252–60.
329. Xu Z, Hu C, Chen S, Zhang C, Yu J, Wang X, *et al.* Apatinib enhances chemosensitivity of gastric cancer to paclitaxel and 5-fluorouracil. *Cancer Manag Res* **2019**; 11:4905–15.
330. Xu J, Yan B, Zhang L, Zhou L, Zhang J, Yu W, *et al.* Theabrownin induces apoptosis and tumor inhibition of hepatocellular carcinoma HUH7 cells through ask1-jnk-c-jun pathway. *Onco Targets Ther* **2020**;13:8977–87.
331. Yagdi Efe E, Mazumder A, Lee J-Y, Gaigneaux A, Radogna F, Nasim MJ, *et al.* Tubulin-binding anticancer polysulfides induce cell death via mitotic arrest and autophagic interference in colorectal cancer. *Cancer Lett* **2017**;410:139–57.
332. Yamamoto D, Sato D, Nakayama H, Nakagawa Y, Shimada Y. ZF-Mapper: Simple and Complete Freeware for Fluorescence Quantification in Zebrafish Images. *Zebrafish* **2019**;16:233–9.
333. Yan C, Do D, Yang Q, Brunson DC, Rawls JF, Langenau DM. Single-cell imaging of human cancer xenografts using adult immunodeficient zebrafish. *Nat Protoc* **2020**;15:3105–28.
334. Yang X, Chen G, Yu S, Xu C, Xin Y, Li T, *et al.* TGF-β1 enhances tumor-induced angiogenesis via JNK pathway and macrophage infiltration in an improved zebrafish embryo/xenograft glioma model. *Int Immunopharmacol* **2013**;15:191–8.
335. Yang X-J, Cui W, Gu A, Xu C, Yu S-C, Li T-T, *et al.* A Novel Zebrafish Xenotransplantation Model for Study of Glioma Stem Cell Invasion. *PLoS One* **2013**;8:1–9.
336. Yang X, Cui W, Yu S, Xu C, Chen G, Gu A, *et al.* A Synthetic dl-Nordihydroguaiaretic acid (Nordy), Inhibits Angiogenesis, Invasion and Proliferation of Glioma Stem Cells within a Zebrafish Xenotransplantation Model. *PLoS One* **2014**;9:e85759.
337. Yang J, Shimada Y, Olsthoorn RCL, Snaar-Jagalska BE, Spaink HP, Kros A. Application of Coiled Coil Peptides in Liposomal Anticancer Drug Delivery Using a Zebrafish Xenograft Model. *ACS Nano* **2016**;10:7428–35.
338. Yang J, Pei H, Luo H, Fu A, Yang H, Hu J, *et al.* Non-toxic dose of liposomal honokiol suppresses metastasis of hepatocellular carcinoma through destabilizing EGFR and inhibiting the downstream pathways. *Oncotarget* **2017**;8:915–32.
339. Yang B, Wang N, Wang S, Li X, Zheng Y, Li M, *et al.* Network-pharmacology-based identification of caveolin-1 as a key target of Oldenlandia diffusa to

suppress breast cancer metastasis. *Biomed Pharmacother* **2019**;112:108607.
340. Yang Y, Hao E, Pan X, Tan D, Du Z, Xie J, *et al.* Gomisin M2 from Baizuan suppresses breast cancer stem cell proliferation in a zebrafish xenograft model. *Aging (Albany NY)* **2019**;11:8347–61.
341. Yang K, Yang JQ, Luo SH, Mei WJ, Lin JY, Zhan JQ, *et al.* Synthesis of N-2(5H)-furanonyl sulfonyl hydrazone derivatives and their biological evaluation in vitro and in vivo activity against MCF-7 breast cancer cells. *Bioorg Chem* **2021**;107:104518.
342. Yao D, Li C, Jiang J, Huang J, Wang J, He Z, *et al.* Design, synthesis and biological evaluation of novel HDAC inhibitors with improved pharmacokinetic profile in breast cancer. *Eur J Med Chem* **2020**;205:112648.
343. Ye Y, Zhang T, Yuan H, Li D, Lou H, Fan P. Mitochondria-Targeted Lupane Triterpenoid Derivatives and Their Selective Apoptosis-Inducing Anticancer Mechanisms. *J Med Chem* **2017**;60:6353–63.
344. Ye S, Liu Y, Fuller AM, Katti R, Ciotti GE, Chor S, *et al.* TGFβ and hippo pathways cooperate to enhance sarcomagenesis and metastasis through the hyaluronan-mediated motility receptor (HMMR). *Mol Cancer Res* **2020**;18:560–73.
345. Yee NS, Kazi AA, Yee RK. Translating Discovery in Zebrafish Pancreatic Development to Human Pancreatic Cancer: Biomarkers, Targets, Pathogenesis, and Therapeutics. *Zebrafish* **2013**;10:132–46.
346. Yu CI, Chen CY, Liu W, Chang PC, Huang CW, Han KF, *et al.* Sandensolide induces oxidative stress-mediated apoptosis in oral cancer cells and in zebrafish xenograft model. *Mar Drugs* **2018**;16(10):387.
347. Yu J, Zhong B, Jin L, Hou Y, Ai N, Ge W, *et al.* 2-Methoxy-6-acetyl-7-methyljuglone (MAM) induced programmed necrosis in glioblastoma by targeting NAD(P)H: Quinone oxidoreductase 1 (NQO1). *Free Radic Biol Med* **2020**;152:336–47.
348. Yuan M, White D, Resar L, Bar E, Groves M, Cohen A, *et al.* Conditional reprogramming culture conditions facilitate growth of lower-grade glioma models. *Neuro Oncol* **2020**;noaa263.
349. Yue Z, Wang A, Zhu Z, Tao L, Li Y, Zhou L, *et al.* Holothurian glycosaminoglycan inhibits metastasis via inhibition of P-selectin in B16F10 melanoma cells. *Mol Cell Biochem* **2015**;410:143–54.
350. Zanin R, Pegoraro S, Ros G, Ciani Y, Piazza S, Bossi F, *et al.* HMGA1 promotes breast cancer angiogenesis supporting the stability, nuclear localization and transcriptional activity of FOXM1. *J Exp Clin Cancer Res* **2019**;38(1):313.
351. Zeng A, Ye T, Cao D, Huang X, Yang Y, Chen X, *et al.* Identify a Blood-Brain Barrier Penetrating Drug-TNB using Zebrafish Orthotopic Glioblastoma Xenograft Model. *Sci Rep* **2017**;7(1):13472.
352. Zeng Q, Liu Y min, Liu J, Han J, Guo J xin, Lu S, *et al.* Inhibition of ZIP4 reverses epithelial-to-mesenchymal transition and enhances the radiosensitivity in human nasopharyngeal carcinoma cells. *Cell Death Dis* **2019**;10(8):588.
353. Zhang S, Cao Z, Tian H, Shen G, Ma Y, Xie H, *et al.* SKLB1002, a novel potent inhibitor of VEGF receptor 2 signaling, inhibits angiogenesis and tumor growth in vivo. *Clin Cancer Res* **2011**;17:4439–50.
354. Zhang B, Shimada Y, Kuroyanagi J, Nishimura Y, Umemoto N, Nomoto T, *et al.* Zebrafish xenotransplantation model for cancer stem-like cell study and high-throughput screening of inhibitors. *Tumor Biol* **2014**;35:11861–9.

355. Zhang B, Shimada Y, Kuroyanagi J, Umemoto N, Nishimura Y, Tanaka T. Quantitative Phenotyping-Based In Vivo Chemical Screening in a Zebrafish Model of Leukemia Stem Cell Xenotransplantation. *PLoS One* **2014**;9:e85439.
356. Zhang B, Shimada Y, Kuroyanagi J, Ariyoshi M, Nomoto T, Shintou T, *et al.* In vivo selective imaging and inhibition of leukemia stem-like cells using the fluorescent carbocyanine derivative, DiOC5(3). *Biomaterials* **2015**;52:14–25.
357. Zhang Q, Hou D, Luo Z, Chen P, Lv B, Wu L, *et al.* The novel protective role of P27 in MLN4924-treated gastric cancer cells. *Cell Death Dis* **2015**;6:e1867.
358. Zhang L, Lu J, Qiu L. Synergistic effects of A-B-C-type amphiphilic copolymer on reversal of drug resistance in MCF-7/ADR breast carcinoma. Int J *Nanomedicine* **2016**;11:5205–20.
359. Zhang Y, Wang P, Zhang Q, Yao X, Zhao L, Liu Y, *et al.* eIF3i activity is critical for endothelial cells in tumor induced angiogenesis through regulating VEGFR and ERK translation. *Oncotarget* **2017**;8:19968–79.
360. Zhang HZ, Li CY, Wu JQ, Wang RX, Wei P, Liu MH, *et al.* Anti-angiogenic activity of para-coumaric acid methyl ester on HUVECs in vitro and zebrafish in vivo. *Phytomedicine* **2018**;48:10–20.
361. Zhang Y, Hou Q, Li X, Zhu J, Wang W, Li B, *et al.* Enrichment of novel quinazoline derivatives with high antitumor activity in mitochondria tracked by its self-fluorescence. *Eur J Med Chem* **2019**;178:417–32.
362. Zhang L, Yan B, Meng S, Zhou L, Xu Y, Du W, *et al.* Theaflavin Induces Apoptosis of A375 Human Melanoma Cells and Inhibits Tumor Growth in Xenograft Zebrafishes Through P53- and JNK-Related Mechanism. *Front Pharmacol* **2020**;11:1–10.
363. Zhang H, Tian F, Jiang P, Qian S, Dai X, Ma B, *et al.* Solasonine Suppresses the Proliferation of Acute Monocytic Leukemia Through the Activation of the AMPK/FOXO3A Axis. *Front Oncol* **2021**;10:614067.
364. Zhao H, Tang C, Cui K, Ang B-T, Wong STC. A screening platform for glioma growth and invasion using bioluminescence imaging. *J Neurosur*g **2009**;111:238–46.
365. Zhao C, Wang X, Zhao Y, Li Z, Lin S, Wei Y, *et al.* A Novel Xenograft Model in Zebrafish for High-Resolution Investigating Dynamics of Neovascularization in Tumors. Black KL, editor. *PLoS One* **2011**;6:e21768.
366. Zhao C, Qiao Y, Jonsson P, Wang J, Xu L, Rouhi P, *et al.* Genome-wide profiling of AP-1-regulated transcription provides insights into the invasiveness of triple-negative breast cancer. *Cancer Res* **2014**;74:3983–94.
367. Zhao C, Zhang W, Zhao Y, Yang Y, Luo H, Ji G, *et al.* Endothelial Cords Promote Tumor Initial Growth prior to Vascular Function through a Paracrine Mechanism. *Sci Rep* **2016**;6:1–13.
368. Zhao X, Li L, Yu G, Zhang S, Li Y, Wu Q, *et al.* Nucleus-enriched Ruthenium Polypyridine Complex Acts as a Potent Inhibitor to Suppress Triple-negative Breast Cancer Metastasis In vivo. *Comput Struct Biotechnol J* **2019**;17:21–30.
369. Zhao LJ, Zhao HY, Wei XL, Guo FF, Wei JY, Wang HJ, *et al.* The lipid homeostasis regulation study of arenobufagin in zebrafish HepG2 xenograft model and HepG2 cells using integrated lipidomics-proteomics approach. J Ethnopharmacol **2020**;260:112943.
370. Pan Y, Zheng M, Zhong L, Yang J, Zhou S, Qin Y, *et al.* A Preclinical Evaluation of SKLB261, a Multikinase Inhibitor of EGFR/Src/VEGFR2, as a Therapeutic Agent against Pancreatic Cancer. *Mol Cancer Ther* **2015**;14:407–18.

371. Zhou R, Curry JM, Roy LD, Grover P, Haider J, Moore LJ, *et al.* A novel association of neuropilin-1 and MUC1 in pancreatic ductal adenocarcinoma: role in induction of VEGF signaling and angiogenesis. *Oncogene* **2016**;35:5608–18.
372. Zhou B, Li H, Xing C, Ye H, Feng J, Wu J, *et al.* Honokiol induces proteasomal degradation of AML1-ETO oncoprotein via increasing ubiquitin conjugase UbcH8 expression in leukemia. *Biochem Pharmacol* **2017**;128:12–25.
373. Zhu Z, Zhao Y, Li J, Tao L, Shi P, Wei Z, *et al*. Cryptotanshinone, a novel tumor angiogenesis inhibitor, destabilizes tumor necrosis factor-α mRNA via decreasing nuclear-cytoplasmic translocation of RNA-binding protein HuR. *Mol Carcinog* **2016**;55:1399–410.
374. Zhu XY, Guo DW, Lao QC, Xu YQ, Meng ZK, Xia B, *et al.* Sensitization and synergistic anti-cancer effects of Furanodiene identified in zebrafish models. *Sci Rep* **2019**;9:1–12.
